# Systematic examination of publicly-available information reveals the diverse and extensive corporate political activity of the food industry in Australia

**DOI:** 10.1186/s12889-016-2955-7

**Published:** 2016-03-22

**Authors:** Melissa Mialon, Boyd Swinburn, Steven Allender, Gary Sacks

**Affiliations:** World Health Organization Collaborating Centre for Obesity Prevention, Deakin University, Burwood, Victoria Australia; School of Population Health, University of Auckland, Auckland, New Zealand

**Keywords:** Food industry, Corporate political activity, Non-communicable diseases

## Abstract

**Background:**

The political influence of the food industry, referred to as corporate political activity (CPA), represents a potential barrier to the development and implementation of effective public health policies for non-communicable diseases prevention. This paper reports on the feasibility and limitations of using publicly-available information to identify and monitor the CPA of the food industry in Australia.

**Methods:**

A systematic search was conducted for information from food industry, government and other publicly-available data sources in Australia. Data was collected in relation to five key food industry actors: the Australian Food and Grocery Council; Coca Cola; McDonald’s; Nestle; and Woolworths, for the period January 2012 to February 2015. Data analysis was guided by an existing framework for classifying CPA strategies of the food industry.

**Results:**

The selected food industry actors used multiple CPA strategies, with ‘information and messaging’ and ‘constituency building’ strategies most prominent.

**Conclusions:**

The systematic analysis of publicly-available information over a limited period was able to identify diverse and extensive CPA strategies of the food industry in Australia. This approach can contribute to accountability mechanisms for NCD prevention.

**Electronic supplementary material:**

The online version of this article (doi:10.1186/s12889-016-2955-7) contains supplementary material, which is available to authorized users.

## Background

In Australia, as in many countries, unhealthy diets are one of the main risk factors for disability and deaths [1]. The food industry, represented by a diverse range of actors, is recognised as having a major influence on the diet of the population through their products, their marketing and their efforts to shape, directly or indirectly, government policies in its favour (referred to as “corporate political activity”, CPA, defined as “corporate attempts to shape government policy in ways favourable to the firm” [2]) [3, 4]. The CPA of the food industry has previously been categorised into six strategies, based on well-established classifications of the CPA of the tobacco industry: information and messaging; financial incentives; constituency building; legal strategies; policy substitution; opposition fragmentation and destabilisation [5] (see Additional file 1 for a description of each strategy). There is emerging evidence and wide-spread concern in the public health community that the CPA of the food industry could pose a risk to the development and implementation of effective policies and programs for non-communicable disease (NCD) prevention and control [6–10]. This is of particular concern for companies who sell products that contribute to the NCD epidemic [4]. The CPA of the food industry is not routinely monitored; however, some instances of unfavourable food industry influence (from a public health perspective) have been described in the literature. As an example, in Australia, a recent report suggested that industry self-regulation (policy substitution strategy) has failed to protect children from marketing of unhealthy food products [11]. In addition, in 2014, it was revealed that close relationships between Australian policy makers, including a former Assistant Health Minister, and the food industry (constituency building strategy), led to delays in the implementation of a public education resource designed to support a new government food labelling initiative [12].

In order to protect public health policies and outcomes from vested interests in the food industry, some public health advocates have called for a strengthening of accountability mechanisms and for more transparency from the food industry [13, 14]. Identifying and monitoring the CPA of major food industry actors has the potential to contribute to these objectives, and this paper reports on the implementation of an approach for doing this using publicly-available information, based on methods used to monitor CPA strategies of the tobacco industry [5].

This study examined the feasibility and limitations of implementing the proposed approach in Australia.

## Methods

A systematic approach was implemented in Australia to identify and monitor the CPA of the food industry, consisting of a document analysis of publicly-available information, based on methods previously used to monitor the CPA of the tobacco industry [5].

Data collection focused on five of the most prominent food industry actors in the country. The actors were selected based on methods previously developed for monitoring the policies and practices of the food industry [15], with the aim of selecting one actor from each of the main sectors within the food industry as well as a major trade association. Euromonitor was used for the identification and selection of these key food industry actors, based on their market shares in 2013 [16]. Where the market leader in a particular sector of the industry did not have an Australian-specific website, the next most dominant company in that sector was selected. The selected food industry actors included: the Australian Food and Grocery Council (AFGC) (a major trade association); Coca Cola (including The Coca Cola Company and Coca Cola Amatil) (a sweetened beverages company); McDonald’s (a fast-food restaurant); Nestle (a processed food products company); and Woolworths (a supermarket).

Systematic searches were conducted across various sources of information [5]. Food industry materials included country-specific website and Twitter accounts of the selected industry actors. Government material included websites of departments and other agencies responsible for health; websites of the Parliament and Senate; register of lobbyists; websites of the three major political parties; websites of commissions in charge of elections; and official requests for information regarding government interactions with the selected food industry actors. All searches were conducted at the national level. Additional sources of information included the websites of: ten major universities with a school/department of nutrition/dietetics/exercise or physical activity; five major professional bodies working on diet-, public health- or physical activity-related issues and related annual conferences. The selection of relevant sources was informed by a pilot study conducted in December 2013, and made in consultation with public health experts in Australia. Media materials, such as Google News and media releases from the selected food industry actors, were also analysed. Details about specific sources of information included in this study are provided in Additional file 2. Data collection was performed between September 2014 and February 2015. For annual or occasional events, such as submissions to public consultations, elections, or conferences, the study included the most recent data available (up to two years retrospectively), as detailed in Additional file 2.

A qualitative thematic analysis was performed by MM. GS re-analysed all data for the selected food industry actors. As themes were not necessarily mutually exclusive, any differences in coding were resolved by mutual agreement. Choice of themes was inductive and based on an existing framework for classifying the CPA of the food industry [5]. Researchers followed an iterative process, where the framework would be adapted to include any new practices identified. However, no new practices were observed as part of this project.

Illustrative examples of CPA strategies used by each food industry actor are presented here, using the framework as a guiding thread. A critical social science approach guided the conduct of this research, with the food industry considered as a potential determinant of health. In this context, the critical social science approach seeks to critique current social conditions as part of efforts to improve population health [17]. Specifically, this research is part of efforts to increase the transparency and accountability of the food industry.

## Results

In total, 310 pieces of information were collected. All references and data collected are included in Additional file 3 (S3), with each piece of information allocated a unique code (starting with the letter A). Table 1 presents a summary of all practices and strategies identified during the period of data collection in Australia.

**Table 1:** Summary of CPA practices identified in Australia

Strategy	Practice	Australian Food and Grocery Council	Coca Cola	McDonald’s	Nestle	Woolworths	Total (occurrences)	
Information and messaging	Lobbying	0	1	1	1	1	4	148
	Stress the economic importance of the industry	4	1	1	1	2	9	
	Promote de-regulation	5	0	0	2	1	8	
	Frame the debate on diet- and public health-related issues	12	19	4	13	3	51	
	Shape the evidence base on diet and public health-related issues	14	9	2	51	0	76	
Financial incentives	Financial incentives	2	5	0	0	5	12	12
Constituency building	Establish relationships with key opinion leaders and health organisations	0	1	0	9	3	13	127
	Seek involvement in the community	2	46	26	10	12	96	
	Establish relationships with policymakers	11	0	1	3	3	18	
	Establish relationships with the media	0	0	0	0	0	0	
Legal strategies	Use legal action (or the threat of) against public policies or opponents	0	0	0	0	0	0	1
	Influence the development of trade and investment agreements	1	0	0	0	0	1	
Policy substitution	Policy substitution	6	1	3	10	2	22	22
Opposition fragmentation and destabilisation	Opposition fragmentation and destabilisation	0	0	0	0	0	0	0
Total number of CPA practices identified		57	83	38	100	31	310	310

Five of the six CPA strategies were identified through publicly-available information: information and messaging; financial incentives; constituency building; legal strategies; and policy substitution. The constituency fragmentation and destabilisation strategy was not identified in the study.

Data related to the AFGC and Nestle were predominantly related to the information strategy; whereas for Coca Cola, McDonald’s and Woolworths, it mainly related to the constituency building strategy.

### Information and messaging strategy

The most frequently observed strategy during data collection was the ‘information and messaging’ strategy. This strategy mainly consists of sharing information and framing messages that depict the industry in a positive way.

As part of the information and messaging strategy, evidence was found that all five food industry actors highlighted their economic importance in efforts to convey a positive image for their industry (A21-4, A110, A168, A202, A302-3). For example, in 2012, in its response to a consultation on nutrition, health and related claims, the AFGC noted that it “makes a substantial contribution to the Australian economy and is vital to the nation’s future prosperity” (A21). There was evidence that the AFGC, Nestle and Woolworths promoted deregulation when discussing diet- or public health-related issues (A16-20, A200-1, A308). They used different arguments against proposed regulation of claims, such that it would discourage innovation (A18, A308); that it would be costly to the industry (the AFGC suggested that it would “cost millions of dollars” (A16)) and be resource intensive (A201); and that it would undermine the competitiveness of businesses (A19, A201). Apart from this consultation, the AFGC also suggested that “regulation should be imposed only where necessary to correct market failure and that it should be sufficiently flexible to encourage innovation” (A18).

Data illustrated that all five food industry actors framed the debate on diet- and public health-related issues in ways that: shift the blame away from the food industry in the NCDs epidemic (the food industry actors rather focused on personal responsibility and on the lack of physical activity); promote the good intentions and stress the good traits of the food industry (for example, the fact that the food industry ensures food safety); emphasise the food industry’s actions to address public health-related issues (including the fact that the food industry promotes healthier lifestyles; stress that the food industry is an important part of the solution, is an expert on diet- and public health- related issues, and provides healthy/healthier versions of its products) (Table 2). The AFGC, Coca Cola and Nestle were found to use this practice most actively of the selected food industry actors.

**Table 2:** Mechanisms and arguments used by the sample of food industry actors to frame the debate on diet- and public health-related issues in Australia

		Australian Food and Grocery Council	Coca Cola	McDonald’s	Nestle	Woolworths
Mechanisms	Arguments	Example of arguments identified during data collection				
Shift the blame away from the food industry	Personal responsibility – people need to have a balanced diet and there are no bad food products, only bad diets	“[There is a] well established paradigm that an individual’s good health is dependent upon a balanced diet” (A26)	“Coke can be consumed as part of a sensible, balanced diet” (A113)	“[When kids eat a] mix of foods – (…) does it balance out with (…) energy requirements?” (A175)	“The basic principle in nutrition [is] that there are no ‘good’ and ‘bad’ foods but rather ‘good’ and ‘bad’ diets” (A203)	“The importance of a balanced diet” (A304)
	Personal responsibility – people need to be more active and balance kilojoules in and out (focus on obesity rather than NCDs)	“The health risks associated with obesity are largely controlled if a person is physically active and physically fit” (A29)	“We know that you’ve got to balance kilojoules in with kilojoules out” (A111)	“If your school or club (…) requires equipment, uniforms or something else that encourages participation in sport, we are happy to help” (A171)	The Nestle Healthy Kids Program contains a lot of information on physical activity (A205)	Not identified
Promote the good intentions and stress the good traits of the food industry	Industry provides safe products	“AFGC advocates a positive role for the food, beverage and grocery industry in providing safe products to consumers” (A32)	Not identified	“Providing customers with safe food is our first priority and our most critical responsibility” (A172)	“As an industry, we’re showing we’re credible partners, going beyond using our scientific knowhow to put micronutrients safely in a product and ensure they’re preserved until the end of its shelf life” (A194)	Not identified
Emphasise the food industry’s actions to address public health-related issues	Industry promotes healthy lifestyles	“The food industry [is] already actioning plan to (…) encourage healthy lifestyle choices” (A35)	Not identified	“Mac Pack: a sporting movement for kids promoting healthy living through fun and play” (A169)	“The Nestle Good Life Program is a group of community initiatives (…) promoting active lifestyles” (A206)	Not identified
	Industry is part of the solution	“Obesity and overweight is a major issue globally. (…) AFGC and the entire food and grocery manufacturing industry are committed to being part of the solution to this critical issue” (A 32)	“We just want to be part of the solution” (A111)	Not identified	“We believe that we have a shared responsibility” (A207)	“We have an important role to play in promoting balanced and healthy eating habits that support a healthy lifestyle” (A304)
	Industry is an expert in diet- and public health-related issues	Not identified	Not identified	Not identified	“To have the greatest possible impact [with our Nestle Good Life Program], we focus on areas where we believe we can add the most value: food, nutrition, and health and wellness. These are areas where we can best contribute our expertise, scientific insight and decades of experience” (A206)	Not identified
	Industry provides healthy/healthier versions of its products	“To help people achieve this balance, industry provides a range of nutritious products, in a variety of portion sizes with low-joule, low-fat, low-sugar and low-salt foods available” (A32)	“We continue to make positive changes. Here’s just a taste of what we’ve achieved. 1. Increasing the availability of our smaller portion sizes. 2. Offering more low kilojoule options” (A115)	Not identified	Not identified	“We have already made significant steps to promote healthy diets to Australian shoppers” (A304)
	Personal responsibility – Industry provides information	“We aim to empower people and communities to make informed choices to improve the health of their families” (A31)	“More information equals more informed consumers, and we believe informed consumers are the ones that make the best decisions for themselves and their families” (A121)	“Happy Meal Choices menu (…) enables parents and children to select meal components to suit individual tastes and dietary requirements” (A170, A178)	“Nestle aims to help parents and children make healthier choices, running cookery schools and educational programmes around the world” (A213)	“We strongly believe our customers should have access to a full suite of nutritional information to enable them to make informed decisions when selecting groceries” (A306)

The data showed that four of the five food industry actors shaped the evidence base on diet- and public health-related issues so that it would favour the industry, as illustrated in Table 3. Publicly-available information revealed that the AFGC and Nestle have been funding their own research, and have been actively promoting it to the public. For example, the AFGC retweeted a message saying that “regularly eating #cereal4brekkie is assoc. w lower BMI & lower risk of being overweight or obese in adults & kids” (A46). The study mentioned was funded by the AFGC itself, through a third party, the Australian Breakfast Cereal Manufacturers Forum (#cereal4brekkie). The AFGC, Coca Cola and Nestle also promoted research that was funded by the food industry (directly or indirectly, through third parties) or where authors had ties with the industry, as well as non-peer reviewed or unpublished evidence (such as information extracted from poster presentations from conferences) (A40, A42, A44, A46, A135, A231, A234-8, A249, A256-9, A261, A268). For example, in its response to a public consultation on the draft for the Australian Dietary Guidelines and for the Australian Guide to Healthy Eating, the AFGC referred to research that had ties with Dairy Australia, the Australian Beverages Council, Coca Cola or Meat and Livestock Australia (A39). All food industry actors, except Woolworths, were found to have sponsored or presented their work in major scientific events or conferences on diet- or public health- related issues. For example, Coca Cola sponsored a session on weight loss maintenance during the 2014 Nutrition Society of Australia Annual Scientific Meeting (A133). The AFGC, McDonald’s and Nestle provided education materials to schools, parents and the public more generally (A49, A158, A160, A175, A206, A210, A224, A227, A229-30, A233-8). Nestle, on its “Nestle Healthy Active Kids” website, provided detailed information on physical activity (A204, A225), while its “Healthy Active Kids Booklet” contained recipes promoting the company’s products (A224). Nestle also organised events in supermarkets during school holidays in which people could receive advice from dietitians, as well as free diabetes testing performed by the Australian Diabetes Council (A228). They could also “learn how Nestlé products are the ideal partners to help you invite more fresh food into your diet” (A228).

**Table 3:** Mechanisms used by the sample of food industry actors to shape the evidence base on diet- and public health- related issues in Australia

	Australian Food and Grocery Council	Coca Cola	McDonald’s	Nestle	Woolworths
Mechanisms	Examples identified during data collection				
Fund research, including through academics, ghost writers, own research institutions and front groups	Promotion (industry website, Twitter, etc.) of research from a front group: “This review was commissioned and paid for by the Australian Breakfast Cereal Manufacturers Forum of the Australian Food and Grocery Council.” (A40)	Not identified	Not identified	“[The] Nestle Research Center (NRC) (…) 250 scientists publish some 200 peer-reviewed scientific publications each year across areas including nutrition and health, public nutrition and food consumer interaction.”(A257)	Not identified
Pay scientists as advisers, consultants or spokespersons	Not identified	“Coca-Cola Australia has an advisory council of experts in the area of obesity, public health and nutrition, who provide advice and counsel to the Company” (A139)	Not identified	Not identified	Not identified
Cite research that has been funded (directly or indirectly, through third parties) by the industry	AFGC submission to the draft Australian Dietary Guidelines and Australian Guide to Healthy Eating: references research funded by the food industry (or with authors that have declared interests with the food industry): Dairy Australia, Australian Beverages Council, Coca Cola, Meat and Livestock Australia. (A39)	“While they contribute minimal kilojoules to the diet, people question the role of diet soft drinks when managing their weight. […] A new study funded by the American Beverage Association and published in the journal Obesity may just have provided evidence to suggest otherwise.” (A134)	Not identified	“[A] recent study carried out by Zurich’s ETH University and Nestle (…) showed that serving school-age children a greater variety of vegetables increased the quantity they chose to consume.” (A257)	Not identified
Disseminate and use non-peer reviewed or unpublished evidence	Not identified	'Infographics on sweeteners on industry websites contain evidence that has not been peer reviewed (e.g., Calorie Control Council) (A135)	Not identified	Nestle Australia Response to Australian Dietary Guidelines - Incorporating the Australian Guide to Healthy Eating - Draft for Public Consultation (2012) includes information drawn from a poster presentation (A215, A268)	Not identified
Participate in and host scientific events	Dietitians Association of Australia 31st National Conference - Sponsored Breakfast Seminars: Healthier Australia Commitment (A38)	2014 Nutrition Society of Australia Annual Scientific Meeting - session sponsored by Coca Cola - “Do small changes make a big difference? Insights into weight loss maintenance research.” – Presented by: Professor James Hill, Denver University, USA (A133)	Dietitians Association of Australia 31st National Conference - Exhibitors: McDonald’s Australia	Dietitians Association of Australia 31st National Conference - Sponsored Breakfast Seminars: Nestle Corporate: “Unlocking the facts on kid’s snack habits” (A217)	Not identified
Provide industry-sponsored education materials	“Details of planned activities for the Dietary Guidelines Work Program - Communication and Implementation Plan 2012: AFGC (…) indicated that they will have some of their own educations initiatives developed by May 2012” (A49)	Not identified	McDonald’s junior development basketball programs in partnership with Basketball Victoria: School resources - lessons plan (A175)	Nestle Healthy Active Kids “with resources for teachers […]. As part of the program [Nestle] distributed 80,000 Kids Nutrition Plates, 50,000 Healthy Active Kids booklets and as a result was able to reach 5,000 teachers and 250,000 school children.” (A224)	Not identified

The only evidence that the selected sample of food industry actors were formally lobbying policy makers was contained in the Australian public Register of Lobbyists. All companies, except the AFGC, were registered as clients of lobbying businesses (A130, A173, A216, A307). However, the nature of the register did not allow any examination of the extent, timing or nature of lobbying activities.

### Financial incentives strategy

The financial incentives strategy, through which the industry provide funds (and other incentives) to policy makers, was identified in Australia through the companies’ annual reports, the annual returns of political parties, the Register of Members of the Parliament’s Interests and Freedom of Information logs. These documents revealed that the AFGC, Coca Cola and Woolworths regularly donated funds to Australian political parties (A14-5, A105-9, A297-301). Coca Cola, for example, donated AUD 55,000 to the Australian Labour Party and to the Liberal Party of Australia for the financial year 2013-14 (A108-9). Woolworths’ political contributions exceeded AUD 35,000 in 2014 (A297).

### Constituency building strategy

There was evidence that all food industry actors included in the sample have established relationships with various health organisations, community groups, and policy makers.

Publicly-available information showed that Coca Cola, Nestle and Woolworths have established relationships with health organisations. Partners included the Sports Dietitians of Australia (A104); the Dietitians Association of Australia (A188-9, A195); the Heart Foundation (A192); the Glycemic Index Foundation (A188, A191, A196); and Nutrition Australia (A292-3).

The food industry actors also sought involvement in the community, and examples identified in Australia during data collection are presented in Table 4. Coca Cola developed an initiative to promote physical activity for kids, called “The Happiness Cycle”, in partnership with an Australian charity (A111). This could also be classified as a “framing” practice, since Coca Cola affirmed that the initiative was part of a program to “help curb obesity” and that the company wanted “to be part of the solution” (A111). In October 2014, McDonald’s promoted its annual “Mc Happy Day”, during which AUD 2 were donated to its charity (Ronald McDonald House) for every purchase of a specific burger (A141). McDonald’s also supported a number of physical activity initiatives, and there was evidence that some of these initiatives helped the industry to promote its brand. For example, children wear red and yellow equipment with the McDonald’s logo and played with the Ronald McDonald mascot as part of the Mac Pack Basketball Super Clinic (A144) and the Swimming Queensland partnership (A149).

**Table 4:** Community initiatives supported by the sample of food industry actors in Australia

		Australian Food and Grocery Council	Coca Cola	McDonald’s	Nestle	Woolworths
Type of activity supported	Population targeted	List of community initiatives identified during data collection				
Physical activity	Under 18s	Not identified	1.Sport Camps Australia (A59) 2.Get Involved (Australian Paralympic Committee) (A74) 3.Happiness Cycle (A111) 4.University of South Australia (SA)’s Football United Program (A103)	1.Macca’s Grassroots Western Australia (WA) (A143) 2.Mac Pack Basketball Super Clinic (A144) 3.Little Athletics WA (A148) 4.Macca’s Cup – Under 18s SA National Football League competition (A150)	1.Cricket Australia (A179) 2.Swim Kids Operation 10,000 (A181)	Not identified
	All ages	Not identified	1.Ride2Work Day (A76)	1.Swimming Queensland (A149)	Not identified	1.Tennis Australia (A289)
Other health- related initiatives	Under 18s	Not identified	1.Youth Focus (A61) 2.Ronald McDonald House Charities (A80)	1.Ronald McDonald House Charities (RMHC) (A141)	1.School Canteen Association (A186)	1.Countdown Kids Hospital Appeal (A280) 2.Variety, the children’s charity, New South Wales (NSW) and Australian Capital Territory (A280) 3.Children’s Hospital Foundation, Queensland (A280) 4.Royal Children’s Hospital Foundation, Victoria and Tasmania (A280)
	All ages	Not identified	1.Red project (HIV) (A73)	1.Association for the Blind of WA (A151) 2.Lifeline WA (A157) 3.Telethon, WA (A152) 4.Cystic Fibrosis fundraising event “Great Strides” (A156)	Not identified	1.Avner Nahmani Pancreatic Cancer Foundation (A280) 2.Royal Flying Doctor Service, SA and Northern Territory (A280) 3.Telethon, WA and Queensland (A280, A285) 4.Bundaberg Health Services Foundation (A290)
Education	Under 18s	Not identified	1.Top Blokes Foundation (A65) 2.Wirrpanda Foundation (A66) 3.AIME (A77) 4.Street University (A70) 5.Galilee School (A75) 6.Australian Indigenous Mentoring Experience (A83) 7.Stacy’s New School (A90)	Not identified	Not identified	1.Earn & Learn (A280)
	All ages	Not identified	1.Mum’s School (A64) 2.Enactus program (A78) 3.Charitable Foundation for Books (A67)	1.The Charlie Bell Scholarship for Future Leaders (A147)	Not identified	Not identified
Other	All ages	1.Foodbank Australia (hunger relief) (A9) 2.Arnott’s Foundation Gala Ball & Charity Auction (A2)	1.Red Shield Appeal, Salvation Army (poverty relief) (A58) 2.Mission Australia (poverty relief) (A79) 3.Keep Australia Beautiful (environment) (A60) 4.Landcare Australia (environment) (A88) 5.Bushfires in Victoria (natural disaster) (A88) 6.Cana Farm, NSW (social) (A63) 7.Brotherhood of St. Laurence African Australian Community Centre, Victoria (social) (A68) 8.Graffiti artists (art) (A62) 9.Beacon Foundation (youths) (A83) 10.Clontard Foundation (youths) (A83) 11.Jack’s House program (youths) (A72) 12.Marist Youth Care Centre, Victoria (youths) (A69, A98) 13.Fitted for Work (employment) (A86, A99)	1.Salvation Army Youth Camp, WA (poverty relief) (A153) 2.McDonald’s Community Cinemas (social) (A145) 3.Earth Hour, WA (environment) (A155) 4.Clean up Australia, WA (environment) (A154)	1.Foodbank Australia (hunger relief) (A182, A184) 2.World Wild Fund Pakistan (environment) (A183) 3.Cyclone Marcia, Queensland (natural disaster) (A184) 4.Nestle Golden Chef’s Hat Award (cooking) (A180) 5.Tamworth Local Aboriginal Land Council (aboriginals) (A185)	1.Foodbank Australia (hunger relief) (A281) 2.Salvation Army (poverty relief) (A280-1) 3.Love Food Hate Waste, NSW (environment) (A279) 4.Bushfires and drought (natural disaster) (A280) 5.Fundraising BBQs (social) (A282)

There was evidence that all food industry actors, except Coca Cola, developed relationships with policy makers in Australia. The “Australian Food and Health Dialogue” is an example of a public-private initiative in which food industry actors have the opportunity to interact with government officials (A5-6, A140, A167, A176, A274, A294-5). In parallel, in October 2014, a Senator and two Members of Parliament participated in the “AFGC Annual Industry Leaders Forum”, held in Parliament House in Canberra (A9). Discussions included topics such as “industry engagement in the political process”. Another forum was planned for October 2015, with the aim to “engage with senior Federal Ministers to better understand government policy direction and how business and government can work in partnership” and to “promote the interests of [the food] industry in the development of policy [and] to showcase the Australian food, beverage and grocery industry to high level stakeholders” (A11). Policy makers themselves also proactively engaged with the food industry in Australia. The then Prime Minister, Tony Abbott, in his speech during the 2014 AFGC Annual Industry Leaders Forum, declared ““I (…) promised that we would cut red tape – and that indeed is happening” (A13). Some Members of Parliament also declared shareholdings in Coca Cola and Woolworths.

### Legal strategies

This study did not find evidence that the selected food companies used legal strategies in this area, through challenging public policies or its opponents in court. However, there was an indication that the food industry attempts to influence the development of trade and investments agreements. For example, on the AFGC’s website, one of the media releases revealed that “the AFGC has, and continues to, engage on the range of trade negotiations underway calling for improved outcomes on processed and semi-processed agri-food products.” (A51).

### Policy substitution strategy

During data collection, all food industry actors were found to use the policy substitution strategy, which consists of proposing voluntary initiatives and self-regulation as part of efforts to avoid the introduction of mandatory regulation. The “Food and Health Dialogue” (A5-6, A140, A167, A176, A274, A294-5), the “Responsible Children’s Marketing Initiative” (A54) and the “Quick Service Restaurant Initiative for Responsible Advertising and Marketing to Children” (A54, A176-7) are examples of voluntary initiatives involving the food industry. The AFGC also welcomed the fact that the “Health Star Rating System” for food products labelling was proposed as a “voluntary scheme with an extended, five year, implementation period, [that could] coexist with the industry supported Daily Intake Guide and other existing front of pack labelling schemes” (A56). The AFGC, McDonald’s and Nestle promoted their efforts to reduce the amount of salt, sugar or fat in their food products (which could also be considered as a “framing” practice) (A178, A269, A271-2, A276-7, A310).

### Opposition fragmentation and destabilisation strategy

In Australia, the opposition destabilisation and fragmentation strategy was not observed during data collection.

## Discussion

This study found evidence that major Australian food industry actors engage in diverse and extensive practices which can have an influence on public health policies and programs. The evidence, identified from publicly-available information only, related mostly to the ‘information and messaging’ and ‘constituency building’ CPA strategies.

This study revealed that different actors employed different practices during the data collection period. The AFGC and Nestle made extensive use of the ‘information and messaging strategy’, particularly by framing the debate and shaping the evidence base on diet- and public health-related NCDs. In contrast, Coca-Cola, McDonald’s and Woolworths focused more on their involvement in the community. It is not clear whether these observed differences are ‘accidental’ or if they reflect fundamental differences between these companies and the environments in which they operate. While differences in management philosophies, competitive pressures in the different sub-sectors of the food industry, and public perceptions are likely to influence the activities of each food industry actor, the reasons that they adopt particular strategies warrant detailed investigation.

This study is, to the authors’ knowledge, the first attempt to use a systematic approach to identify and monitor CPA strategies of the food industry in Australia and other high-income countries (HICs). The study was able to identify a seemingly large and diverse number of activities that can be classified as CPA. It can be expected that this systematic approach could be similarly employed in other countries with similar political systems to monitor food industry CPA in those countries.

Most of the strategies and practices identified by this study were similar to strategies previously identified as being used by the food industry in other countries [6–9]. The findings also reflect strategies used by actors in other industries, such as the alcohol and tobacco industries [4, 18–20].

This study has a number of limitations.

Most notably, the study only collected publicly-available information. Due to the nature of CPA, publicly-available information is likely to give an incomplete picture of the full range of practices adopted by the food industry. Lobbying of policy makers, for example through personal connections, gifts and other private interactions are not readily documented in the public domain. Interviews with key stakeholders in the food system and/or a focus on specific case studies, such as specific public health policies or specific periods of time, could help identifying CPA in a more comprehensive way. Additional sources of information, such as social media platforms, crowdsourcing of information, or whistle-blowers forums, could also be added. Moreover, the timing of data collection may have had an impact on the information identified by this study. The data provides only a snapshot of activities employed, but does not provide an indication of how these activities vary over time. It could be expected that food companies adopt different strategies according to different political climates. For example, the ‘policy substitution’ strategy may be more likely to be employed when major public health- or diet-related policies are proposed or developed in a given country, and the ‘financial incentives’ strategy may be more prevalent in the lead up to an election. Longer periods of monitoring over multiple time periods, supplemented by specific detailed case studies, are likely to improve our understanding of CPA strategies employed by food companies over time.

Future investigations could focus on a broader range and a larger sample of food companies, or could focus on the international practices of the food industry actors selected for this study, using their global websites. The activities of third parties that have direct financial or legal associations with the five food industry actors included in this study were not monitored per se, although a list of ‘front groups’ that have direct ties to the industry were identified during the study (Additional file 4) and their activities monitored to some extent. Other third parties, such as public relations firms paid by food companies and that might lobby politicians on behalf of the company, for example, were not included in this study.

There are also a number of limitations with the data sources used. For example, in Australia, declarations of donations to political parties only include amounts “above the disclosure threshold for the financial year ($12,400 for 2013-14)” [21]. Moreover, the Australian Register of Lobbyists only includes names of lobbyists and of their employers, but, unlike the lobby register in the United States, it does not provide additional details [22], such as the number of meetings between lobbyists and politicians, the amount spent on lobbying activities, or the nature of issues being lobbied. For these sources of information, more detailed reporting would improve transparency and enable more informed monitoring. While Freedom of Information (FOI) requests are recommended to be used as part of the methods for systematic identification and monitoring of CPA strategies [5], in Australia, the use of FOI requests to obtain information about government interactions with industry stakeholders only revealed very limited relevant information. This is likely to have been due to the requested information being considered by government officials as commercially sensitive and, therefore, to be redacted in documents obtained through FOI requests.

For other sources of information, such as industry websites, a large amount of relevant data was available, and time constraints forced the researchers to collect only illustrative examples for each practice and for each actor. There is, therefore, scope for conducting detailed case studies for specific industry actor, specific food products, or specific diet- or public health-related issues, which could complement the proposed systematic identification and monitoring of the CPA of the food industry.

Importantly, the information identified in this study only indicates that the food industry actors have employed the practices identified. The study is not able to assess the intentions behind the use of these practices. Indeed, it is common in the business literature to discuss these activities using terms such as ‘corporate social responsibility’, ‘shared value’, partnerships, and public relations [23–25]. In conducting our analysis, our focus is on the potential risk to public health from these activities.

The study is not able to indicate the influence of the identified practices on the community, on public health advocates and researchers, on policy makers, and, ultimately, on the policy process. The specific details of the way in which different industry practices have an influence needs to be the subject of further investigations. For example, researchers could investigate the way in which Coca-Cola’s involvement in the community affects policy makers’ positions on specific policy issues, such as restricting marketing to children.

This research contributes to INFORMAS (International Network for Food and Obesity/NCD Research, Monitoring and Action Support) – an initiative that aims to monitor and benchmark public and private sector actions to create healthy food environments and reduce obesity and NCDs [26]. Data collected as part of this study adds to the growing literature on corporations and public health [3, 4, 18, 27–32], supplements projects that monitor the CPA of other industries in relation to public health [27, 33–35] and can inform public health advocates and policy makers about the practices employed by food industry actors. Increased awareness of the potential risks to public health from these practices can lead to measures to increase transparency in this area (for example, better disclosure of financial contributions to political parties) or to limit industry involvement in policy development processes. This type of research could also contribute to existing efforts to monitor corporate behaviour, such as the work undertaken by the Center for Responsive Politics, Corporate Accountability International and Corporate Europe Observatory [36–39]. While the implementation of this monitoring approach is relatively low cost, close links with established civil society organisations would improve the sustainability of ongoing monitoring.

## Conclusions

This study was able to reveal important details on the CPA strategies used by major food industry actors in Australia, even though data was collected for a limited period of time, and results presented are unlikely to be comprehensive.

Implementing the systematic approach to identify and monitor the CPA of the food industry could help strengthen accountability mechanisms by highlighting the strategies of the food industry and its relationships with governments and other civil society groups. Ultimately, improved disclosure and accountability could help to protect public health policies and outcomes from conflicted commercial interests in the food industry.

## Consent

This project was approved by the Human Ethics Advisory Groups of the Faculty of Health at Deakin University, Australia (project number HEAG-H 145_2014).

## Additional files

Additional file 1: Description of CPA strategies, from Mialon et al. [7]. (DOCX 18 kb) Additional file 2: Sources of information and searches conducted in Australia, based on methods developed by Mialon et al. [7]. (DOCX 43 kb) Additional file 3: Data retrieved during data collection in Australia. (DOCX 175 kb) Additional file 4: Examples of third parties funded or affiliated with the sample of food industry actors in Australia. (DOCX 19 kb)
