# Correction to: Systematic examination of publicly -available information reveals the diverse and extensive corporate political activity of the food industry in Australia

**DOI:** 10.1186/s12889-021-10997-1

**Published:** 2021-06-18

**Authors:** Melissa Mialon, Boyd Swinburn, Steven Allender, Gary Sacks

**Affiliations:** 1 World Health Organization Collaborating Centre for Obesity Prevention, Deakin University, Burwood, Victoria Australia; 2 School of Population Health, University of Auckland, Auckland, New Zealand

**Correction to: BMC Public Health 16, 283 (2016)**

**http://orcid.org/10.1186/s12889-016-2955-7**

It was highlighted that in the original article [1] the in-text citations of Reference [5] and Reference [7] were interchanged. The original article has been updated.
