# Supplementary material for: Systematic examination of publicly-available information reveals the diverse and extensive corporate political activity of the food industry in Australia
Source: BMC Public Health. 2016 Mar 22;16:283. doi: 10.1186/s12889-016-2955-7 (PMC4804618; doi:10.1186/s12889-016-2955-7)
Supplement: Additional file 1: — Description of CPA strategies, from Mialon et al. [7]. (DOCX 18 kb) [file 12889_2016_2955_MOESM1_ESM.docx]

# Additional file 1 - Description of CPA strategies, from Mialon et al. [7]

The information strategy includes practices through which the industry disseminates information that is beneficial to its activities in order to influence public health policies and outcomes in a way that would favour corporations.

Through the financial incentives strategy, the industry provides funds, gifts and other incentives to politicians, political parties and other decision makers.

The aim of the constituency building strategy is to gain the favour of public opinion as well as other stakeholders such as the media and the public health community.

When threatened by regulation, the industry proposes alternatives such as voluntary initiatives or self-regulation.

The industry also sues its opponents and challenges public policies in courts as part of a legal strategy.

Finally, the constituency fragmentation and destabilization strategy refers to the practices employed by the industry to prevent and counteract criticism of a company’s products or practices.

1. Mialon M, Swinburn B, Sacks G: **A proposed approach to systematically identify and monitor the corporate political activity of the food industry with respect to public health using publicly available information**. *Obesity Reviews* 2015, **16**(7):519-530.
