# Supplementary material for: Systematic examination of publicly-available information reveals the diverse and extensive corporate political activity of the food industry in Australia
Source: BMC Public Health. 2016 Mar 22;16:283. doi: 10.1186/s12889-016-2955-7 (PMC4804618; doi:10.1186/s12889-016-2955-7)
Supplement: Additional file 2: — Sources of information and searches conducted in Australia, based on methods developed by Mialon et al. [7]. (DOCX 43 kb) [file 12889_2016_2955_MOESM2_ESM.docx]

Additional file 2: Sources of information and searches conducted in Australia, based on methods developed by Mialon et al. [1]

| Nature of the source of information | Category | Source of information | Specific data to be collected |
| --- | --- | --- | --- |
| Food industry material | Country-specific website of the industry actor | Nestle Australia: http://www.nestle.com.au   https://www.nestlebaby.com.au/  https://www.nestleprofessional.com/australia/en/Insights/Pages/NUTRIPRO.aspx  http://www.nestlechoosewellness.com.au  http://www.nestlehealthscience.com.au/ Coca Cola Australia: https://www.coca-cola.com.au  http://www.coca-colajourney.com.au/  http://ccamatil.com/Pages/default.aspx  http://www.cokecareers.com.au/Pages/default.aspx  http://cokegrads.com.au/ McDonald's Australia: https://mcdonalds.com.au Woolworths: http://www.woolworths.com.au Australian Food and Grocery Council:  http://afgc.org.au  http://www.mydailyintake.net/ | • Composition of diet-related committee • Webpages, reports related to diet-related issues • Voluntary initiatives, commitments and policies related to diet-related issues • Awards to researchers • Research units or groups on diet-related issues • Submissions to public consultations  • Education material about diet-related issues  • Qualitative analysis for information relevant to the conceptual framework [1] |
|  | Country-specific webpages or report or information in annual reports of a company's philanthropic activities | Nestle Australia: http://www.nestle.com.au/creating-shared-value Coca Cola Australia: http://www.coca-colajourney.com.au/ (same as above) McDonald's Australia: https://www.rmhc.org.au/ Woolworths: http://www.woolworths.com.au/wps/wcm/connect/Website/Woolworths/About+Us/Community/ http://www.woolworthslimited.com.au/page/A_Trusted_Company/Corporate_Responsibility/ AFGC: http://www.afgc.org.au/publications/ | Qualitative analysis for information relevant to the conceptual framework [1] |
|  | Country-specific social media accounts of the industry actor | Twitter:  Nestle: https://twitter.com/nestleaunews Coca Cola Australia: https://twitter.com/CocaColaAU  https://twitter.com/CocaColaAmatil McDonald's: https://twitter.com/McDonaldsAu  Woolworths: N/A in Australia AFGC: https://twitter.com/AusFoodGrocery | Qualitative analysis for information relevant to the conceptual framework [1]  Twitter accounts monitored from September 2014 to February 2015. Facebook accounts were monitored from September 2014 to December 2014 but limited CPA was revealed and so monitoring was stopped |
| Government material: departments (and related agencies) responsible for diet- related issues | Websites of departments and related agencies in charge of health (National level) | http://australia.gov.au/news-and-media/public-consultations  Australian government - Public consultations: http://www.australia.gov.au/news-and-media/public-consultations  National Health and Medical Research Council (NHMRC): http://consultations.nhmrc.gov.au/public_consultations/submissions/all  http://consultations.nhmrc.gov.au/public_consultations/submissions/dietary_guidelines http://consultations.nhmrc.gov.au/public_consultations/submissions/obesity department of Health (DoH): http://www.health.gov.au/internet/main/publishing.nsf/Content/health_consulations.htm  Food Standards of Australia and New Zealand (FSANZ): http://www.foodstandards.gov.au/code/proposals/  http://www.foodstandards.gov.au/code/proposals/Pages/proposalp293nutritionhealthandrelatedclaims/Default.aspx | From January 2012 to December 2014: submissions to public consultations from the food industry and its allies (including third parties) on diet- and public health-related issues - Consultations include: nutrition, health and related claims, dietary guidelines and obesity  Qualitative analysis for information relevant to the conceptual framework [1] |
|  |  | DoH   Front of Pack: http://www.health.gov.au/internet/main/publishing.nsf/Content/frontofpackcommittee   NHMRC   Aus dietary guidelines:  http://www.nhmrc.gov.au/your-health/nutrition/dietary-guidelines-working-committee/declarations-conflict-interest-dietary  FSANZ: http://www.foodstandards.gov.au/about/board/Pages/default.aspx  Food and Health Dialogue: http://www.foodhealthdialogue.gov.au/internet/foodandhealth/publishing.nsf/Content/industry-engagement  Australian Research Council Linkage projects: http://www.arc.gov.au/ncgp/lp/lp_outcomes.htm http://www.arc.gov.au/ncgp/itrp/hubs_outcomes.htm | Working groups on diet-related issues and conflicts of interest Public private initiatives |
|  |  | Australian Health Ministers’ Conference media releases and communiques http://www.health.gov.au/internet/main/publishing.nsf/Content/Media+Releases+Communiques-1 | Qualitative analysis for information relevant to the conceptual framework [1] |
|  |  | NHMRC: http://www.nhmrc.gov.au/about/freedom-information DoH: http://www.health.gov.au/internet/main/publishing.nsf/Content/foi-about FSANZ: http://www.foodstandards.gov.au/about/ips/foilog/Pages/default.aspx | Informally asked for: number and dates of meetings between the selected food industry actors and the department, topic of meeting   If no adequate response, Freedom of Information (FOI) requests from January 2012 to December 2014 |
|  |  | FOI log NHMRC: https://www.nhmrc.gov.au/about/freedom-information/freedom-information-disclosure-log DoH: http://www.health.gov.au/internet/main/publishing.nsf/Content/foi-disc-log FSANZ: http://www.foodstandards.gov.au/about/ips/foilog/Pages/default.aspx | FOI disclosure log with information related to the food industry on diet- and public health-related issues, from January 2012 to December 2014 |
|  | Websites of the Parliament and Senate (National level) | http://www.aph.gov.au/Parliamentary_Business/Committees/House/Current_Inquiries http://www.aph.gov.au/Parliamentary_Business/Committees/Senate/Current_Inquiries http://www.aph.gov.au/Parliamentary_Business/Committees/Joint/Current_Inquiries | From January 2012 to December 2014: Submissions to public consultations from the food industry and its allies (including third parties such as front groups) on diet- and public health-related issues - Qualitative analysis for information relevant to the conceptual framework [1] |
|  |  | http://www.aph.gov.au/About_Parliament/Parliamentary_Departments/Information_Requests | Ask for: number and dates of meetings between the industry and the Parliament or Senate, topic of meeting, industry actors If no answer, Freedom of Information requests from January 2012 to December 2014 |
|  |  |  | FOI disclosure log from January 2012 to December 2014 |
|  |  | http://www.aph.gov.au/Senators_and_Members/Members/Register | Declarations of interests of all members |
|  | Register of lobbyists (National level) | http://lobbyists.pmc.gov.au | Lobbyists on behalf of the food industry |
|  | Websites of 3 major political parties and websites of commissions in charge of elections (National Level) | Candidate Election Return - Australian Greens, Australian Labor Party and Liberal Party of Australia -http://electiondisclosures.aec.gov.au/CandidateSearch.aspx?SubmissionId=17496  Political Party Annual Return - Australian Greens, Australian Labor Party and Liberal Party of Australia - http://periodicdisclosures.aec.gov.au/Party.aspx | From January 2012 to December 2014: Annual returns for donations from the food industry (donations for elections and donations to political parties, does not include Public Relations agencies, which are not exclusively working on behalf of the food industry) |
| Other material | 10 major universities with a school/department of nutrition/dietetics/exercise or physical activity | Monash University - department of Nutrition and Dietetics http://www.med.monash.edu.au/ http://www.med.monash.edu.au/scs/nutrition-dietetics/  The University of Sydney -  School of Molecular Bioscience, the Faculty of Science Center for overweight and obesity (ended 2008) Cluster for Public Health Nutrition http://sydney.edu.au/ http://sydney.edu.au/science/molecular_bioscience/ http://sydney.edu.au/medicine/public-health/coo/ (ended 2008)  Deakin University - School of Exercice and Nutrition Science http://www.deakin.edu.au/ http://www.deakin.edu.au/health/ens/  The University of Adelaide - Centre of Research Excellence in Translating Nutritional Science to Good Health http://www.adelaide.edu.au/ http://www.adelaide.edu.au/cre-nutrition/  University of South Australia - Nutritional Physiology Research Centre SANSOM http://www.unisa.edu.au/ http://www.unisa.edu.au/Research/Sansom-Institute-for-Health-Research/Research-at-the-Sansom/Research-Concentrations/Nutritional-Physiology/  Curtin University - School of Public Health Nutrition www.curtin.edu.au/  University of Newcastle - School of Health Science: The Centre for Physical Activity and Nutrition  http://www.newcastle.edu.au/research-and-innovation/centre/cpan/  Queensland University of Technology - Faculty of Health, School - Exercise and Nutrition Sciences https://www.qut.edu.au/ https://www.qut.edu.au/health/about/schools/school-of-exercise-and-nutrition-sciences  University of Tasmania - School of Human Life Science: Nutrition and dietetics http://www.utas.edu.au/ http://www.utas.edu.au/human-life-sciences/future-students/career-opportunities/Health-Science/nutrition-and-dietetics  Flinders University - School of Health Science: Nutrition and dietetics http://www.flinders.edu.au/ http://www.flinders.edu.au/sohs/sites/nutrition-and-dietetics/student-prizes.cfm | Searches on the websites and asked (to vice chancellor, deputy vice chancellor (research) and research services) for   - Funds received or sponsor from the food industry - Research projects, fellowships or grants funded by the selected food industry actors - Prizes to students   If no answer, Freedom of Information requests from January 2012 to December 2014 http://www.adm.monash.edu.au/execserv/foi/ http://sydney.edu.au/legal/regulations/foi.shtml http://www.deakin.edu.au/about-deakin/administrative-divisions/university-solicitors-office/freedom-of-information  http://www.adelaide.edu.au/policies/3/  http://w3.unisa.edu.au/cha/staffinfo/foi/default.asp  http://legal.curtin.edu.au/foi/  http://www.newcastle.edu.au/about-uon/governance-and-leadership/access-to-information/request-information  http://www.mopp.qut.edu.au/F/F_06_03.jsp  http://www.utas.edu.au/legal-services/right-to-information-act  http://www.flinders.edu.au/policyandsecretariat/freedom-of-information/ |
|  | 5 major conferences on diet-, public health- or physical activity-related issues (National level) | Public Health Association of Australia (PHAA) 43rd Annual Conference http://www.phaa.net.au/43rd_Annual_Conference.php  2014 Australian and New Zealand Obesity Society (ANZOS) Annual Scientific Meeting  Dieticians Association of Australia (DAA) National Conference 2014 http://arinex.com.au/dietitians2014/  Nutrition Society of Australia (NSA) 2014 Annual Scientific Meeting http://www.nsa.asn.au/index.php/2014_ASM/  Exercise & Sports Science Australia Conference 2014 www.essa.org.au/2014conference/ | Booths of the food industry |
|  |  |  | Presentations from or supported by the food industry (poster presentation, oral presentation) |
|  |  |  | Sponsors from the food industry |
|  |  |  | Awards from the food industry |
|  | 5 major professional bodies in diet-, public health- or physical activity related health issues (National level) | Public Health Association of Australia: http://www.phaa.net.au/  Australia and New Zealand Obesity Society: https://anzos.com/index.php?option=com_content&view=featured&Itemid=14  Dietitians Association of Australia (DAA): http://daa.asn.au/  Nutrition Society of Australia (NSA): http://www.nsa.asn.au/  Exercise & Sports Science Australia: https://www.essa.org.au/ | Funds received or sponsors from the food industry (if no info, ask phaa@phaa.net.au , https://www.anzos.com/index.php?option=com_contact&view=contact&id=1&Itemid=4 , nationaloffice@daa.asn.au, nsa@theassociationspecialists.com.au, rachel.collins@essa.org.au) |
|  |  |  | Awards from the food industry |
|  |  |  | Qualitative analysis for information relevant to the conceptual framework [1] |
| Media | News and media releases | Search on Google News with the name of the company and “health” Nestle Australia: http://www.nestle.com.au/media/newsandfeatures Coca Cola Australia: http://www.coca-colajourney.com.au McDonald's: https://mcdonalds.com.au/news  Woolworths: http://www.woolworths.com.au/wps/wcm/connect/Website/Woolworths/About+Us/Woolworths-News/  AFGC: http://www.afgc.org.au/media-centre/ http://www.afgc.org.au/category/news/ | Monthly monitoring from September 2014 to February 2015  Qualitative analysis for information relevant to the conceptual framework [1] |

1. Mialon M, Swinburn B, Sacks G: **A proposed approach to systematically identify and monitor the corporate political activity of the food industry with respect to public health using publicly available information**. *Obesity Reviews* 2015, **16**(7):519-530.
