# Supplementary material for: Systematic examination of publicly-available information reveals the diverse and extensive corporate political activity of the food industry in Australia
Source: BMC Public Health. 2016 Mar 22;16:283. doi: 10.1186/s12889-016-2955-7 (PMC4804618; doi:10.1186/s12889-016-2955-7)
Supplement: Additional file 3: — Data retrieved during data collection in Australia. (DOCX 175 kb) [file 12889_2016_2955_MOESM3_ESM.docx]

# Additional file 3: Documents retrieved during data collection in Australia

## Australian Food and Grocery Council

| **Reference in manuscript** | **Source** | **Strategy** | **Practice (code used for analysis)** | **Data coded** | **Notes** | **Website URL** |
| --- | --- | --- | --- | --- | --- | --- |
| A1 | Twitter | Constituency building | Seek involvement in the community | Jason Hincks ‏@JayFoodBank Incredible support last night by @AusFoodGrocery at the annual @FoodbankAus awards dinner, incl. a passionate address by @kevinandrewsmp |  | https://twitter.com/JayFoodBank/status/517513496506810370 |
| A2 | Twitter | Constituency building | Seek involvement in the community | AFGC retweeted Campbell Arnott's @CampbellArnotts · Jan 22 Coming soon - The Annual Arnott’s Foundation Gala Ball & Charity Auction! Go to the website for more details http://bit.ly/14VFBSU |  | https://twitter.com/CampbellArnotts/status/558466769506287616 |
| A3 | Government | Constituency building | Establish relationships with policy makers | 2013/2014 FSANZ Board Appointment round and Update of Register of Interests - July 2014: - Mr Roberts: was the deputy CEO and scientific and technical Director of the Australian Food and Grocery Council for 5 years until October 2007 |  | http://www.foodstandards.gov.au/about/board/Documents/Update%20of%20Register%20of%20Interests%20-%20July%202014.pdf |
| A4 | Government | Constituency building | Establish relationships with policy makers | FSANZ Update of Register of Interests - July 2014: - Dr David Roberts: Made submissions in capacity as Scientific and Technical Director of Australian Food and Grocery Council (retired Oct 2007) in relation to: P235 – Review of Food-type Dietary Supplements P236 – Sports Foods P242 – Food for Special Medical Purposes P274 – Labelling Minimum Age for Infant Foods P293 – Nutrition, Health & Related Claims |  | http://www.foodstandards.gov.au/about/board/Documents/Update%20of%20Register%20of%20Interests%20-%20July%202014.pdf |
| A5 | Government | Constituency building | Establish relationships with policy makers | Food and Health Dialogue - meeting May 2013 - Attendeed: Member from food industry- Mr Gary Dawson, CEO, AFGC  Observer from food industry: Dr Geoffrey Annison, Deputy CEO, Health, Nutrition and Scientific Affairs, AFGC | 8 members and 4 observers |  |
| A6 | Government | Constituency building | Establish relationships with policy makers | The following organisations form membership of the Dialogue: (Food and Health Dialogue) Australian Food and Grocery Council Membership of the Reformulation Working Group comprises: Australian Food and Grocery Council |  | http://www.foodhealthdialogue.gov.au/internet/foodandhealth/publishing.nsf/Content/about-us |
| A7 | Government | Constituency building | Establish relationships with policy makers | Front-of-Pack Labelling Committee and Working Group meetings Technical Design Working Group (TDWG) Meeting 1 - 20 April 2012 The TDWG members have been selected on the basis of their expertise and experience in the following fields and do not represent their organisations as working group members (8 members total): Geoffrey Annison (co-Chair) - Industry (AFGC) Greg Gambrill - Industry (Sanitarium) Rebecca Boustead - Industry/Nutrition (Kellogg) Neil Smith - Industry (Kraft) |  | http://www.health.gov.au/internet/main/publishing.nsf/Content/frontofpackcommittee |
| A8 | Government | Constituency building | Establish relationships with policy makers | Dietary Guidelines Working Committee - Conflict of interest information: Dr Geoffrey Annison is one of the members (11 members) |  | http://www.nhmrc.gov.au/your-health/nutrition/dietary-guidelines-working-committee/declarations-conflict-interest-dietary |
| A9 | Industry | Constituency building | Establish relationships with policy makers | The AFGC Annual Industry Leaders Forum presents a leading opportunity for members to actively engage with key federal Government Ministers, decision-makers and public policy influencers – and help Shape the evidence base on diet- and public health-related issues the food and grocery industry policy agenda.  Industry Leaders Forum Afternoon Session Wednesday 1 October 2014- Parliament House Theatre 3.45pm Senator The Hon Fiona Nash, Assistant Minister for Health 4.15pm Panel discussion – Industry engagement in the political process - Mr Dan Tehan MP, Member for Wannon - Ms Clare O’Neil MP, Member for Hotham, Deputy Chair Standing Committee on Agriculture and Industry (invited)  Evening: Industry Leaders Forum Dinner Venue: Gandel Hall, National Gallery of Australia 8.00pm Industry Leaders Forum Dinner including Presentation of Annual Foodbank Awards by The Hon. Kevin Andrews MP, Minister for Social Services. |  | http://www.afgc.org.au/component/content/article/4-events-sponsorship/1953-2014-industry-leaders-forum.html |
| A10 | Industry | Constituency building | Establish relationships with policy makers | "The AFGC’s Legal and Regulatory Division  advocates best regulatory practice standards of minimum effective regulation and rigorous regulatory impact assessment through submissions to regulators and governments on specific regulatory consultations, enforcement policies and wider economic and regulatory policy inquiries  engages with regulators through formal and informal mechanisms to develop respectful relationships; develops industry compliance and best practice tools" |  | http://www.afgc.org.au/our-expertise/legal-and-regulatory-affairs/ |
| A11 | Industry | Constituency building | Establish relationships with policy makers | Parliament House Canberra, Australia 21 October 2015  The Industry Leaders Forum brings together the senior executives from the top food, grocery and beverage companies. It provides our members with a once-a-year opportunity to actively engage with key government ministers, decision makers and public policy influencers and to hear first- hand about the pressures and federal issues shaping the food and grocery industry policy agenda.  "The Annual Industry Leaders Forum is designed to encourage active discussion between key food, beverage and grocery personnel, retailers, Commonwealth and State politicians, senior government officials and academics. The Industry Leaders Forum focuses on a range of issues of vital importance to industry and provides a platform for industry and stakeholders to examine issues affecting public policy and company operations. The seminar offers delegates an opportunity to **engage with senior Federal Ministers to better understand government policy direction and how business and government can work in partnership** to examine and comment on the major issues facing our industry." "AFGC engages directly with Federal, State and Territory governments to **promote the interests of our industry in the development of policy**. The **Annual Industry Leaders Dinner is designed to showcase the Australian food, beverage and grocery industry to high level stakeholders**. AFGC aims to attract the highest level of attendance from a distinguished guest list for this event. The Industry Leaders Dinner, attracting approximately **150-200 guests**, has built a reputation as **one of Canberra’s premier events** and will continue to develop into a leading function in the calendar year. The guest list for this event includes key personnel from corporate management within the food, beverage and grocery sector, major players in Commonwealth and State parliaments, senior government and international officials, academics and media representatives." | Sponsorship can include "**Preferential dinner seating at a prominent table** (at discretion of AFGC)" | http://www.afgc.org.au/2014/12/industry-leaders-forum/ http://www.afgc.org.au/events/sponsorship-opportunities/ |
| A12 | Industry | Constituency building | Establish relationships with policy makers | "Equally important has been our ability to **push back on various strident demands from pressure groups and legislators** by presenting strong evidence-based policy backed by effective media and political advocacy. (…) The **new Federal Government has set a pro-business policy agenda** with a commitment to reduce cost, remove complexity and facilitate productivity and economic activity. But it is taking time to implement this agenda particularly due to a gridlocked legislative process. (...) The AFGC continues to work with policy makers, government departments, industry players, retailers and non-government stakeholders to **advance the industry's interests.** AFGC has also been at the **forefront of the Government s drive into Asia** and has partnered with **Austrade to bring AFGC members Market Insights on China, Thailand and Malaysia**.(...) There is no doubt that 2014-15 will once again be a challenge for the food and grocery sector and the **AFGC will continue to engage vigorously to shape the regulatory and trading environment**" |  | http://www.afgc.org.au/download/617/ |
| A13 | Industry | Constituency building | Establish relationships with policy makers | Transcript of T.Abbott's speech at the Food Industry Forum 2014: "It is good to be back here at the Food and Grocery Council and I want to say that I've always appreciated the welcome that I've had from you, both in this forum and in your businesses right around our country. I’ve been a regular speaker at this forum for many years since I was the Minister for Workplace Relations back in the middle years of the Howard government" "I also promised that we would cut red tape – and that indeed is happening." "We’re doing everything we can to encourage more and freer trade. We’ve have the free trade agreements finalised with Japan and with Korea and we hope to do so soon with China" "Food and agriculture is not just an important part of our economic past, it is an absolutely vital part of our economic future." |  |  |
| A14 | Political party | Financial incentive | Financial incentives | Political Party Disclosure Return FINANCIAL YEAR 2011-12 Australian Labor Party Name: Australian Food & Grocery Council $8,250 |  | http://periodicdisclosures.aec.gov.au/Returns/49/PNGX9.pdf |
| A15 | Political party | Financial incentive | Financial incentives | Political Party Disclosure Return FINANCIAL YEAR 2013-14 Australian Labor Party Name: Australian Food and Grocery Council $1,500 |  | http://periodicdisclosures.aec.gov.au/Returns/55/SLDJ8.pdf |
| A16 | Government | Information and messaging | Promote deregulation | FSANZ Proposal 293 Nutrition Health and Related Claims Consultation Submission by AFGC: "**millions of dollars in costs that will be imposed on industry** to implement the standard" [page 4] "the costs associated with implementing the standard will **impact the Australian industry heavily, adding further constraints to the hard economic times the industry is currently facing**" [page 10] "With no evidence that any claims currently being used by industry are incorrect or misleading this cost is completely unjustified" [page 15] "sugar is also identified as a nutrient criterion in the NPSC, despite there not being any evidence to support a link between sugar intake, obesity and NCDs " [page 15] "in direct contrast to advice provided by the Commonwealth following the review of food labelling" [page 10] "in direct contrast to the fifth policy principle to be „cost effective overall, not more trade restrictive than necessary and comply with Australia’s and New Zealand’s obligations under the WTO agreements.‟ [page 10] | 32 Pages – Only illustrative examples presented here | http://www.foodstandards.gov.au/code/proposals/Documents/P293_consultation_2012.zip |
| A17 | Government | Information and messaging | Promote deregulation | FSANZ Proposal P1030 - AFGC submission "The AFGC foresees a significant enforcement issue if regulators must prove that a specific function was the basis for the formulation of the drink." [page 1] "the positioning does not require specific regulatory intervention" "unnecessary barriers to trade" [page 2] |  | http://www.foodstandards.gov.au/code/proposals/Pages/P1030HealthclaimsSportsfoods.aspx |
| A18 | Industry | Information and messaging | Promote deregulation | "AFGC supports the Competition Policy Principles of **minimum effective regulation** adopted by the Council of Australian Governments (COAG). The AFGC considers that regulation should be imposed **only where necessary to correct market failure and that it should be sufficiently flexible to encourage innovation**." |  | http://www.afgc.org.au/our-expertise/health-nutrition-and-scientific-affairs/ |
| A19 | Industry | Information and messaging | Promote deregulation | "The Food and Grocery Regulatory Reform Project involving an extensive review and analysis of **excessive regulations** that are impacting the competitiveness of the sector" |  | http://www.afgc.org.au/download/617/ |
| A20 | FOI request | Information and messaging | Promote deregulation | Media release AFGC: Physical activity, diet and health linkage strengthened in new Ministry  [...] The appointments of both Peter Dutton and Senator Nash are a very positive sign that the government will take a balanced approach to food regulation [...]" |  |  |
| A21 | Government | Information and messaging | Stress the economic importance of the industry | FSANZ Proposal 293 Nutrition Health and Related Claims Consultation Submission by AFGC: "With an annual turnover of $108 billion, Australia’s food and grocery manufacturing industry makes a **substantial contribution to the Australian economy and is vital to the nation’s future prosperity**.” [page 2] | 32 Pages - Only illustrative examples presented here | http://www.foodstandards.gov.au/code/proposals/Documents/P293_consultation_2012.zip |
| A22 | Government | Information and messaging | Stress the economic importance of the industry | Australian Food and Grocery Council SUBMISSION TO: NATIONAL HEALTH AND MEDICAL RESEARCH COUNCIL IN RESPONSE TO: THE DRAFT AUSTRALIAN DIETARY GUIDELINES AND AUSTRALIAN GUIDE TO HEALTHY EATING "With an annual turnover of $108 billion, Australia’s food and grocery manufacturing industry makes a substantial contribution to the Australian economy and is vital to the nation’s future prosperity." [page 2] "The growing and sustainable industry is made up of over 30,100 businesses and accounts for $46 billion of the nation’s international trade. The industry spends $368 million a year on research and development. " [page 2] | 45 pages - Only illustrative examples presented here | http://consultations.nhmrc.gov.au/files/consultations/_written_submissions/dietary_guidelines/_id_174_christel_leemhuis_sub.pdf |
| A23 | Industry | Information and messaging | Stress the economic importance of the industry | "Australia’s $114 billion food and grocery processing sector is a vital contributor to the wealth and health of our nation. The industry produces safe nutritious food and other essentials of life for every Australian every day. It’s also sustaining Australia through the provision of jobs and economic opportunities. In 2013-14, the food, beverage and grocery processing industry directly employed almost 300,000 people, with 45% of those jobs located in regional areas. One out of every three jobs in manufacturing in Australia are in the food and grocery sector, with 220,500 people employed in food and beverage processing; 30,295 employed in grocery manufacturing and 48,936 people employed in the fresh produce sector." |  | http://www.afgc.org.au/about-afgc/our-industry/ |
| A24 | Twitter | Information and messaging | Stress the economic importance of the industry | AFGC ‏@AusFoodGrocery  AFGC Welcomes Start of Japan Australia Free Trade Agreement: http://www.afgc.org.au/2015/01/afgc-welcomes-start-of-japan-australia-free-trade-agreement/ … | Media release on their website: http://www.afgc.org.au/2015/01/afgc-welcomes-start-of-japan-australia-free-trade-agreement/ | https://twitter.com/AusFoodGrocery/status/555861826496241664 http://www.afgc.org.au/2015/01/afgc-welcomes-start-of-japan-australia-free-trade-agreement/ |
| A25 | Industry | Information and messaging | Frame the debate on diet- and public health-related issues the debate on diet- and public health-related issues | "We promote the valuable role industry plays in health and nutrition through a range of partnerships with key stakeholders, programs and engagement in public debate." |  | http://www.afgc.org.au/our-expertise/health-nutrition-and-scientific-affairs/ |
| A26 | Government | Information and messaging | Frame the debate on diet- and public health-related issues the debate on diet- and public health-related issues | FSANZ Proposal 293 Nutrition Health and Related Claims Consultation Submission by AFGC: "The use of NPSC imposes nutrient qualifying and disqualifying criteria derived from population dietary advice onto individual food products. This is scientifically flawed. It creates arbitrary boundaries between products and ignores the well-established paradigm that an individual’s good health is dependent upon a balanced diet, containing a variety of foods balanced with adequate physical activity. " [page 20] | 32 Pages - Only illustrative examples presented here | http://www.foodstandards.gov.au/code/proposals/Documents/P293_consultation_2012.zip |
| A27 | Government | Information and messaging | Frame the debate on diet- and public health-related issues the debate on diet- and public health-related issues | Australian Food and Grocery Council SUBMISSION TO: NATIONAL HEALTH AND MEDICAL RESEARCH COUNCIL IN RESPONSE TO: THE DRAFT AUSTRALIAN DIETARY GUIDELINES AND AUSTRALIAN GUIDE TO HEALTHY EATING "overall energy balance (energy in via food consumptions and energy out via activity) is the most important issue and that a focus on sugar within the guidelines require further consideration" [page 21] "AFGC is extremely supportive of Guideline 3, and the importance of physical activity as an essential part of a healthy lifestyle and energy balance. It is essential that the Dietary Guidelines reflect the linkages between food consumption, physical activity and inactivity as critical elements to a healthy lifestyle. Indeed physical activity itself is positively associated with health outcomes, including mitigating the risk of non-communicable diseases, independent of body weight associations." [page 22] | 45 pages - Only illustrative examples presented here | http://consultations.nhmrc.gov.au/files/consultations/_written_submissions/dietary_guidelines/_id_174_christel_leemhuis_sub.pdf |
| A28 | Industry | Information and messaging | Frame the debate on diet- and public health-related issues the debate on diet- and public health-related issues | Together count website:  _Together Counts™ is a nationwide program inspiring **active** and healthy living. The principle behind our program is **energy balance**, which means **balancing the kilojoules we eat and drink with the kilojoules we burn off through activity**. _Achieving **energy balance isn’t just about eating the ‘right’ food; it’s also about being active every day** – it’s the energy ‘in’ versus energy ‘out’ equation | Focused on physical activity  Message promoted on Coca Cola's website: http://www.coca-colajourney.com.au/towards-a-healthier-australia-working-together | http://www.togethercounts.com.au/ http://www.togethercounts.com.au/pledge/ http://www.togethercounts.com.au/energy-balance-in-detail/ http://www.togethercounts.com.au/out-and-about/ http://www.togethercounts.com.au/wp-content/themes/togethercounts2013/img/Infographic.jpg |
| A29 | Industry | Information and messaging | Frame the debate on diet- and public health-related issues the debate on diet- and public health-related issues | Healthier Australia Commitment:  **_In 2007/8, 62% of adults did not meet the recommended physical activity guidelines.** _**78% of adults spend between two and six hours a day sitting at leisure.** _the workplace as the ideal setting to **promote physical activity, producing multiple benefits.** _Within the workplace members will **promote energy balance including developing materials focussed on a healthy lifestyle encompassing the quality and quantity of foods consumed and getting active. _Energy Balance:** For optimal health it is important to **balance our energy,** that is balance the amount of food and beverages consumed (including the quality and quantity) with physical activity. **Being healthy and active** is essential to ensure quality of life and reduce the burden of chronic preventable diet-related diseases. **The health risks associated with obesity are largely controlled if a person is physically active and physically fit.** | No statistics on foods produced by the partners companies (only fruits and vegetables) No resources for statistics Focused on physical activity | http://www.togethercounts.com.au/healthier-australia-commitment/ |
| A30 | Industry | Information and messaging | Frame the debate on diet- and public health-related issues the debate on diet- and public health-related issues | "The long-term success of the food and grocery sector is essential to meeting the everyday needs of the nation’s growing population and is core to the wellbeing of all 22 million Australians, now and many more consumers overseas – their health and quality of life depend on both its existence and excellence." |  | http://www.afgc.org.au/about-afgc/our-policies/ |
| A31 | Industry | Information and messaging | Frame the debate on diet- and public health-related issues the debate on diet- and public health-related issues | "The Healthier Australia Commitment will work to improve the health of Australian’s by working in three key areas: **Community**  The HAC will work with others to **assist in providing consumers with information about the importance of energy balance**, that is, that is, **balancing** the amount and quality of food and beverages consumed with physical activity.  We aim to empower **people and communities to make informed choices** to improve the health of their families. Workplace  The World Health Organisation and the Australian Government have identified the workplace as the ideal setting to **promote physical activity**, producing multiple benefits.  Within the workplace members will promote **energy balance** including developing materials focussed on a healthy lifestyle encompassing the quality and quantity of foods consumed and **getting active**.  The HAC will work with their members and other businesses, to provide and share **resources to promote healthy active workplaces**. Market Place  Australia’s food supply is wondrous and full of **variety for consumers**.  HAC members have committed to optimise the nutritional profile of foods and beverages to help **reduce the intake** of sodium, saturated fat and energy in products." | "reduce the intake" | http://www.afgc.org.au/key-projects/healthy-australia-commitment/ |
| A32 | Industry | Information and messaging | Frame the debate on diet- and public health-related issues | "AFGC advocates a **positive role for the food, beverage and grocery industry in providing safe products to consumers** and helping **Australians make informed diet and lifestyle choices**, leading to health and wellness. " "Obesity and overweight is a major issue globally. (...) AFGC and the entire food and grocery manufacturing industry are **committed to being part of the solution** to this critical issue. We **encourage all Australians to balance a nutritious diet with plenty of physical activity**. To help people achieve this **balance**, industry **provides a range** of nutritious products, in a **variety of portion sizes** with low-joule, low-fat, low-sugar and low-salt foods available." |  | http://www.afgc.org.au/our-expertise/health-nutrition-and-scientific-affairs/ |
| A33 | Industry | Information and messaging | Frame the debate on diet- and public health-related issues | "The AFGC'Healthier Australia Commitment hosted the **Move for Health conference** in partnership with **beyondblue, the Australian Diabetes Council, Osteoporosis Australia and Exercise and Sport Science Australia**. The conference bought together experts in the **use of exercise and physical activity to treat medical conditions**." "The Together Counts campaign featuring **Olympian Susie O'Neill** continues to **promote the energy in/ energy out health messag**e through providing healthy diet and lifestyle tips to consumers. |  | http://www.afgc.org.au/download/617/ |
| A34 | FOI request | Information and messaging | Frame the debate on diet- and public health-related issues | FOI 2014-15 010 request - Combined Release Documents NHMRC Discussion with the Australian Food and Grocery Council on the Dietary Guidelines:  AFGC have also had a long-standing position that ‘there are no bad foods, only poor diets’. AFGC therefore has always been highly critical of any government activity that identifies particular foods or food components as having a negative effect on health. |  |  |
| A35 | FOI request | Information and messaging | Frame the debate on diet- and public health-related issues | Media Release AFGC: Food industry already actioning plan to reduce salt and encourage healthy lifestyle choices |  |  |
| A36 | FOI request | Information and messaging | Frame the debate on diet- and public health-related issues | Media release AFGC: Physical activity, diet and health linkage strengthened in new Ministry  […] "Physical inactivity is a major public health issue, gaining increasing recognition as an area needing urgent action," Mr Dawson said.  [...] The AFGC has long advocated for a broader consideration of energy balance - the relationship between energy consumed and energy expended through physical activity - in the development of health policy, particularly in relation to obesity and non-communicable disease. (see document for more) |  |  |
| A37 | Government | Information and messaging | Shape the evidence base on diet- and public health-related issues evidence | Australian Food and Grocery Council SUBMISSION TO: NATIONAL HEALTH AND MEDICAL RESEARCH COUNCIL IN RESPONSE TO: THE DRAFT AUSTRALIAN DIETARY GUIDELINES AND AUSTRALIAN GUIDE TO HEALTHY EATING "there was no systematic literature review undertaken on the link between energy density and obesity. Thus there appears to be no justification for the use of the term ‘energy dense’ in the Dietary Guidelines text." [page 18] On saturated fat: “this recommendation would not be actionable by consumers and will eliminate many nutritious foods from the diet" [page 19] "As noted under discussion on the Dietary Guidelines, the exclusion of discussion on discretionary foods from the Dietary Guidelines and the AGHE is a significant omission, and contrary to ensuring the AGHE is actionable." [page 27] | 45 pages - Only illustrative examples presented here | http://consultations.nhmrc.gov.au/files/consultations/_written_submissions/dietary_guidelines/_id_174_christel_leemhuis_sub.pdf |
| A38 | Conference | Information and messaging | Shape the evidence base on diet- and public health-related issues the evidence base on diet- and public health-related issues evidence | Dietitians Association of Australia 31st National Conference - Sponsored Breakfast Seminars: Australian Food & Grocery Council: Healthier Australia Commitment |  | http://arinex.com.au/dietitians2014/wp-content/uploads/2014/05/DAA-Conference-2014-Program-Only-PR-Version.pdf http://arinex.com.au/dietitians2014/sponsored-breakfast-seminars/ |
| A39 | Government | Information and messaging | Shape the evidence base on diet- and public health-related issues evidence | Australian Food and Grocery Council SUBMISSION TO: NATIONAL HEALTH AND MEDICAL RESEARCH COUNCIL IN RESPONSE TO: THE DRAFT AUSTRALIAN DIETARY GUIDELINES AND AUSTRALIAN GUIDE TO HEALTHY EATING  References to research funded by the food industry (or with authors that have conflicts of interest with the food industry) | 45 pages - Only illustrative examples presented here   - “The role of reducing intakes of saturated fat in the prevention of cardiovascular disease: where does the evidence stand in 2010?”: “unrestricted grants were received from […] Dairy Australia […]” http://www.ncbi.nlm.nih.gov/pmc/articles/PMC3138219/ - “Beverage intake and obesity in Australian children”: “Flinders University was paid to do the secondary analysis of the Children's Nutrition Survey by the Beverage Council of Australia and Peter Clifton was paid to write the manuscript by Coca Cola South Pacific” http://www.nutritionandmetabolism.com/content/8/1/87 | http://consultations.nhmrc.gov.au/files/consultations/_written_submissions/dietary_guidelines/_id_174_christel_leemhuis_sub.pdf |
| A40 | Industry | Information and messaging | Shape the evidence base on diet- and public health-related issues the evidence base on diet- and public health-related issues evidence | "On 16 September 2014, a systematic literature review spanning more than 230 papers over 30 years was published in Advances in Nutrition, an international peer-reviewed journal of the American Society of Nutrition1. The review, The Benefits of Breakfast Cereal Consumption: A Systematic Review of the Evidence Base is the first scientific review commissioned by the Australian Breakfast Cereal Manufacturers Forum, a member-funded forum of the Australian Food and Grocery Council." | Reference to a website - a front group: http://www.cereal4brekkie.org.au/abcmf/  "The Australian Breakfast Cereal Manufacturers Forum (ABCMF) " Reference to a paper: "This review was commissioned and paid for by the Australian Breakfast Cereal Manufacturers Forum of the Australian Food and Grocery Council." http://advances.nutrition.org/content/5/5/636S.full | http://www.afgc.org.au/2014/09/breakfast-cereals-and-health-a-systematic-review-of-the-evidence/ |
| A41 | Industry | Information and messaging | Shape the evidence base on diet- and public health-related issues the evidence base on diet- and public health-related issues evidence | "AFGC promotes the **scientific facts about food and good nutrition**. We advocate **sound nutrition** principles based on a **whole-of-diet approach rather than focusing on particular types of food**. This includes correcting misinformation about food, nutrition and health." |  | http://www.afgc.org.au/our-expertise/health-nutrition-and-scientific-affairs/ |
| A42 | Industry | Information and messaging | Shape the evidence base on diet- and public health-related issues the evidence base on diet- and public health-related issues evidence | “The ABCMF provides evidence-based, practical information so Australian can have a better understanding of the true value of breakfast cereals and breakfast as part of a healthy lifestyle, by emphasising the benefits of breakfast cereals, correcting misinformation and engaging in a positive dialogue with Australian media influencers and consumers. Highlights for 2013-2014 include:  ABCMF contributed to an increase in positive share of voice about breakfast cereals across print and broadcast media.  A successful **public relations campaign "To Eat or Tweet"** plus provision of regular content to media and commentators via articles/news on the website.  Launch of **new Twitter account @cereal4brekkie**.  Increase in website hits of nearly 300per cent.  A significant increase in budget enabled expansion of programs and additional staffing.  A **systematic review of the scientific evidence on breakfast cereals, nutrition and health** was completed and accepted for publication in a scientific journal.  Annual consumer tracking research and new consumer research to support communications strategy.  Current projects include: Industry serve sizes; Nutritional profile of the category; reanalysis of the Australian Health Survey data on the breakfast cereal category. | Funded and supported by the AFGC | http://www.afgc.org.au/download/617/ |
| A43 | News | Information and messaging | Shape the evidence base on diet- and public health-related issues the evidence base on diet- and public health-related issues evidence | An industry-backed centre dedicated to creating healthier food choices for Australian and Asian consumers opened at UQ this week.[…] Led by The University of Queensland, the centre will combine the expertise of principal partner the Australian Food and Grocery Council, as well as collaborating partners the International Rice Research Institute, Rural Industries Research and Development Corporation, Wuhan University and Huazhong University of Science |  | http://www.uq.edu.au/news/article/2014/10/industry-backs-training-healthier-food |
| A44 | Twitter | Information and messaging | Shape the evidence base on diet- and public health-related issues the evidence base on diet- and public health-related issues evidence | AFGC retweeted cereal4brekkie @cereal4brekkie · Jan 19 A #delicious breakfast choice AND it’s high in #protein! For more high protein brekkie http://bit.ly/1xqeibp | And then link to the front group's website: http://www.cereal4brekkie.org.au/cereal-solutions-higher-protein-brekkies/ Promotion of a study funded by the industry: "If managing your weight is one of your goals for 2015, starting the day right is key to success. A recent review of 30 years of research found regularly eating breakfast cereal is associated with a lower BMI and a reduced risk of being overweight, compared to people who skip breakfast or chose other brekkie foods. But with all the hype around higher protein diets for weight loss, what are the higher protein options when it comes to breakfast cereal? Accredited Practising Dietitian and Australian Breakfast Cereal Manufacturers’ Forum Director Leigh Reeve says breakfast cereals offer something for everyone.  “There’s a tasty range of higher protein breakfast cereals on the market or you can simply boost the protein of your favourite cereal with some quick and easy food pairings that don’t require expensive ingredients or supplements,” said Ms Reeve.  “Brekkies providing around 20g of protein may be recommended to help regulate appetite and increase satiety.2 This can be particularly important at breakfast time as it may help you avoid the mid-morning munchies.”" | https://twitter.com/cereal4brekkie/status/557292399299272704 |
| A45 | Twitter | Information and messaging | Shape the evidence base on diet- and public health-related issues the evidence base on diet- and public health-related issues evidence | AFGC retweeted Together Counts AU @TogetherAU · Feb 2 What tips do you have for boosting the veggie content of the school lunchbox? Go! http://on.fb.me/1zOlYe6 | Then link to the Facebook page of "Together counts" | https://twitter.com/TogetherAU/status/562467514404175873 |
| A46 | Twitter | Information and messaging | Shape the evidence base on diet- and public health-related issues the evidence base on diet- and public health-related issues evidence | AFGC retweeted cereal4brekkie @cereal4brekkie · Feb 5 Regularly eating #cereal4brekkie is assoc. w lower BMI & lower risk of being overweight or obese in adults & kids http://bit.ly/1A1r58d | Already twitted many times - link to a study funded by the industry: http://www.cereal4brekkie.org.au/new-science/ | https://twitter.com/cereal4brekkie/status/563453509610127362 |
| A47 | Uni | Information and messaging | Shape the evidence base on diet- and public health-related issues the evidence base on diet- and public health-related issues evidence | University of South Australia - Annual Food Industry Forum for Nutrition Research The forum is an annual event aimed at fostering dialogue between nutrition researchers and food manufacturers to exploit recent advances in nutritional science for the benefit of all stakeholders. [...] The 3rd Annual Food Industry Forum for Nutrition Research Healthy Food for a Healthier Australia was held on Monday 3 September 2012 [...]. Major sponsors of the Forum were the Australian Food and Grocery Council and Nestle Australia. Supporting sponsors were Simplot Australia, Grains & Legumes Nutrition Council, Unilever Australia, Mannatech Australasia, the Nutrition Society of Australia, Kellogg's, Meat & Lifestock Australia and Sanitarium.  Public Private Partnerships in Preventative Health:  "Australians unable to select a balanced diet" "Evidence Based Consider all options Regulate last" "Best Practice Regulation Market failure (and Government failure) corrected" "Food and food supply has a role to play" "Challenge for industry – to be part of the solution, not the part of the problem" | Use of a picture from the Center for Consumer Freedom (front group from the USA): https://www.consumerfreedom.com/2007/06/somethings-just-not-right/ | http://www.unisa.edu.au/Research/Sansom-Institute-for-Health-Research/Research-at-the-Sansom/Research-Concentrations/Nutritional-Physiology/Annual-Food-Industry-Forum-for-Nutrition-Research/  http://www.unisa.edu.au/Global/Health/Sansom/Documents/FIF/2012/geoffrey_annison_2012.pdf |
| A48 | Uni | Information and messaging | Shape the evidence base on diet- and public health-related issues the evidence base on diet- and public health-related issues evidence | University of Newcastle - CRC for Evidence-Based Health Food and Supplements Participants: Australian Food and Grocery Council |  | http://www.newcastle.edu.au/research-and-innovation/innovation/crc-bids/ebhfs#participants http://www.newcastle.edu.au/__data/assets/pdf_file/0005/115295/CRC-propspectusweb.pdf |
| A49 | FOI request | Information and messaging | Shape the evidence base on diet- and public health-related issues the evidence base on diet- and public health-related issues evidence | FOI 2014-15 010 request - Combined Release Documents NHMRC: Details of planned activities for the Dietary Guidelines Work Program - Communication and Implementation Plan 2012: AFGC have indicated that they will have some of their own educations initiatives developed by May 2012 This may be an opportunity to engage the industry more broadly in promotional activities for the launch of the Dietary Guidelines  [...] AFGC would like to be involved in promotional activities for the Dietary Guidelines post-launch. Interested in developing consistent education messages. |  |  |
| A50 | FOI request | Information and messaging | Shape the evidence base on diet- and public health-related issues the evidence base on diet- and public health-related issues evidence | FOI 2014-15 010 request - Combined Release Documents NHMRC: Discussions on the draft Australian Dietary Guidelines with the Australian Food & Grocery Council: the AFCG disagreed with many recommendations made for the Dietary Guidelines (see document for examples) | Criticise evidence |  |
| A51 | Industry | Legal | Influence the development of trade and investment agreements | The AFGC has, and continues to engage on the range of trade negotiations underway calling for improved outcomes on processed and semi-processed agri-food products. The AFGC and Agribusiness Forum provided submissions to the Government, and appeared before Parliamentary Committees on the Japan and Korea trade agreements, and continue to engage on other negotiations including with China, and Trans Pacific Partnership countries. |  | http://www.afgc.org.au/2014/11/international-trade-newsletter/ |
| A52 | Government | Policy substitution | Policy substitution | FSANZ Proposal 293 Nutrition Health and Related Claims Consultation Submission by AFGC: "AFGC recommends that Australia’s regulatory system for nutrition and health claims comprise a c**ombination of full regulation for High level claims complemented by an industry Code of Practice for General level claims** " [page 16] | 32 Pages - Only illustrative examples presented here | http://www.foodstandards.gov.au/code/proposals/Documents/P293_consultation_2012.zip |
| A53 | Industry | Policy substitution | Policy substitution | "AFGC is committed to ensuring that **consumers are provided** with high quality, safe and appropriately labelled products. This should be done through **minimum effective regulation so that companies can continue to produce affordable products and be cost competitive**. (...) The **Daily Intake Guide helps consumers make easy, smart choices about the food they need to include in their diet** and ultimately helps consumers see the relationship between a serve of food and their daily requirements. (...) The Daily Intake Guide is a **voluntary scheme**, which has been developed by AFGC in consultation with a range of stakeholders including the Dietitians Association of Australia." |  | http://www.afgc.org.au/key-projects/daily-intake-labelling/ |
| S3 – 54 | Industry | Policy substitution | Policy substitution | "The Australian food industry is aware that **the community is concerned** about the advertising of discretionary foods to children and have implemented codes of practice to **demonstrate industry responsiveness to consumer issues** such as these. There are two **self-regulatory initiatives** managed by AFGC that specifically address food and beverage advertising to children, namely the Responsible Children’s Marketing Initiative (RCMI), which covers products found in retail outlets and the Quick Service Restaurant Initiative for Responsible Advertising and Marketing to Children (QSRI), which covers food sold in quick service restaurants." | Companies that have signed up to the initiatives commit to:  Only advertising **healthier choices** to children and encouraging a healthy lifestyle through good diet and **physical activity**.  Not paying for or seeking product placement television programs, editorial content or interactive games aimed at children, **unless the product is a healthier choice**.  Not advertising and marketing to children in Australian schools **unless they are asked to by those schools.** http://www.afgc.org.au/our-expertise/industry-codes/advertising-to-children/ See Sack et al 2014, Critical PH, for analysis | http://www.afgc.org.au/our-expertise/industry-codes/ |
| S3 – 55 | Industry | Policy substitution | Policy substitution | "The Code of Practice for Food Labelling and Promotion (the Code) sets out provisions that guide the way the food industry communicates with consumers through labels on food packages, through labels associated with unpackaged foods, and in advertising, promotional and point-of-sale material. The Code has been developed as a mechanism for the food industry to **aid consumers in their decision-making** through the provision of consistent and accurate information about food products." | http://www.afgc.org.au/our-expertise/industry-codes/code-of-practice-for-food-labelling-and-promotion/ See Sacks et al 2014, Critical PH, for analysis | http://www.afgc.org.au/our-expertise/industry-codes/ |
| S3 – 56 | Industry | Policy substitution | Policy substitution | "In June 2014, **Food Regulation Forum Ministers endorsed the AFGCs preferred position** for the Health Star Rating (HSR) scheme to be a **voluntary** scheme with an **extended, five year, implementation period**, and allow for it to **coexist with the industry supported** Daily Intake Guide and other existing front of pack labelling schemes. The AFGC confirmed its continuing support for the Daily Intake Guide" |  | http://www.afgc.org.au/download/617/ |
| S3 – 57 | FOI request | Policy substitution | Policy substitution | Media Release AFGC: Food industry already actioning plan to reduce salt and encourage healthy lifestyle choices (see document for more information) |  |  |

## Coca Cola

| **Reference in manuscript** | **Source** | **Strategy** | **Practice (code used for analysis)** | **Data coded** | **Notes** | **Website URL** |
| --- | --- | --- | --- | --- | --- | --- |
| A58 | Charity | Constituency building | Seek involvement in the community | The Salvation Army today launched its annual Red Shield Appeal at The Westin Sydney […]. The event, proudly sponsored by Jones Lang LaSalle, [...] joined by the Director of Media Coca-Cola Amatil, Sally Loane. |  | http://salvos.org.au/about-us/latest-news/media-newsroom/20140501-rsa-sydney-launch/ |
| A59 | Charity | Constituency building | Seek involvement in the community | Sports Camps Australia Commercial Partners: SPC Ardmonia |  | http://www.sportscampsaustralia.com.au/partners/ |
| A60 | Charity | Constituency building | Seek involvement in the community | Keep Australia Beautiful - Sponsors: The Coca Cola Foundation and Coca Cola South Pacific |  | http://kab.org.au/sponsors/ |
| A61 | Industry | Constituency building | Seek involvement in the community | Youth Focus provides a crucial suicide-prevention service through early intervention for young people suffering depression, yet it largely depends on the generosity of donors and corporate partners. A recent grant from Coca-Cola means the organisation can continue offering free counselling services to young people and their families. |  | http://www.coca-colajourney.com.au/coke-in-the- community/choose-life-free-program-focuses-on-suicide-prevention |
| A62 | Industry | Constituency building | Seek involvement in the community | Together with some of the finest graffiti artists in the country, Paul takes groups of kids to walls around town on which it is legal for them to paint, so the artists can teach them the finer details of street art. A grant from Coca-Cola Australia Foundation enabled him to invest in materials for the program. |  | http://www.coca-colajourney.com.au/coke-in-the- community/paint-the-town-street-art-enlivens-lismore |
| A63 | Industry | Constituency building | Seek involvement in the community | Cana Farm, an oasis offering opportunities to marginalised people in western Sydney and supported by the Coca-Cola Australia Foundation. |  | http://www.coca-colajourney.com.au/stories/spring-in-the-garden-tips-from-cana-farm |
| A64 | Industry | Constituency building | Seek involvement in the community | Mum’s School is the only one of its type in the Northern Territory. The YWCA of Darwin depends on the support of donors such as the Coca-Cola Australia Foundation to fund the initiative. |  | http://www.coca-colajourney.com.au/coke-in-the- community/mums-the-word-school-support-for-young-mothers |
| A65 | Industry | Constituency building | Seek involvement in the community | A grant from Coca-Cola has allowed the Top Blokes Foundation to extend its mentorship program to more high schools throughout the country. |  | http://www.coca-colajourney.com.au/coke-in-the- community/boys-will-be-men-fostering-top-blokes |
| A66 | Industry | Constituency building | Seek involvement in the community | A grant from Coca-Cola assisted the Wirrpanda Foundation to employ a mentor four days a week at Doonside Technology High School. |  | http://www.coca-colajourney.com.au/coke-in-the-community/deadly-sister-girlz-why-young-women-are-having-a-yarn-with-the-wirrpanda-foundation |
| A67 | Industry | Constituency building | Seek involvement in the community | The Charitable Foundation for Books in Homes Australia is run in communities throughout Australia, from Sydney on the east coast to the Kimberley region on the west. The program allows budding readers to select books for themselves and take them home. [...] A recent grant from the Coca-Cola Australia Foundation enabled the organisation to continue a program in Queensland. |  | http://www.coca-colajourney.com.au/coke-in-the-community/read-all-about-it-a-unique-program-gets-books-into-the-homes-and-hearts-of-children |
| A68 | Industry | Constituency building | Seek involvement in the community | [...]In their new home in Western Melbourne, the Brotherhood of St. Laurence African Australian community Centre is working to make life a little easier. [...] Through a grant from Coca-Cola Australia Foundation, the centre is establishing an African Social Club to target the area’s young people. |  | http://www.coca-colajourney.com.au/stories/making-life-a-little-easier-building-communities-in-melbournes-west |
| A69 | Industry | Constituency building | Seek involvement in the community | "[…] The Marist Youth Care Centre in Brunswick, however, helps young people through the basics of finding and holding down a job. […]A recent grant from Coca-Cola Australia Foundation allows the centre to provide healthy lunches." |  | http://www.coca-colajourney.com.au/coke-in-the-community/on-the-job-centre-prepares-youth-for-work |
| A70 | Industry | Constituency building | Seek involvement in the community | “Big issues that we deal with are young people and a lot of drugs and alcohol. Health and homelessness are also big issues in the community,” explained Roueida. Recognising this, the Coca-Cola Australia Foundation recently provided a grant to the Street University to educate young people about the importance of physical activity and healthy eating. |  | http://www.coca-colajourney.com.au/coke-in-the-community/new-school-of-thought-ted-noffs-street-university-is-training-entrepreneurs |
| A71 | Industry | Constituency building | Seek involvement in the community | [Duplicate] Cana Farm has been supported by the Coca-Cola Australia Foundation. Click here for more information on grant applications, or check out our Coke in the community stories. | 2 articles for the same program - the other was on 23 11 2014 | http://www.coca-colajourney.com.au/stories/beginners-guide-to-gardening |
| A72 | Industry | Constituency building | Seek involvement in the community | The Jack’s House program, which is supported by the Coca-Cola Australia Foundation, is run in concert with initiatives that help develop life-skills. Transition to Independent Living, for example, helps young people gain - and maintain - their own rental tenancies. Another program, Branch Out, encourages kids to go back to school or find employment. |  | http://www.coca-colajourney.com.au/coke-in-the-community/finding-shelter-at-jacks-house-how-a-gladstone-group-is-rewriting-better-endings |
| A73 | Industry | Constituency building | Seek involvement in the community | [Duplicate]  Many Australians believe the threat of AIDS is behind us. But, in reality, 650 babies are born HIV positive every day. The good news, however, is that a world without AIDS is just within reach. To help fight the spread of this incurable disease, Coca-Cola is partnering with (RED) to raise funds to end mother-to-child transmission of HIV. Ahead of World AIDS Day on December 1, Coca-Cola and (RED)’s Share The Sound Of An AIDS-Free Generation campaign uses new music to raise money for life-saving medication. Each week, an exclusive new track from artists including Queen, Aloe Blacc and Avicii will be released via iTunes.  [...]Coca-Cola is a long-time partner in the fight against AIDS, supporting (RED) with $5 million since 2011. | Another article on 18 Nov 2014 - pictures look like an ad for Coca Cola (red, logo, etc.) | http://www.coca-colajourney.com.au/coke-in-the-community/red-alert-coca-cola-invites-music-fans-to-share-the-sound-of-an-aids-free-generation http://www.coca-colajourney.com.au/coke-in-the-community/wyclef-jean-raps-about-his-new-track-for-the-coca-cola-red-campaign-featuring-avicii |
| A74 | Industry | Constituency building | Seek involvement in the community | 18 Nov 2014: Up and coming athletes like Ethan now have a better chance than ever to participate in sport via Get Involved, an initiative developed by the Australian Paralympic Committee through funding from the Coca-Cola Australia Foundation. The program aims to get youths with a disability participating in sport.  2 Nov 2014: The program, created after the Australian Paralympic Committee received new funding from the Coca-Cola Australia Foundation, is taking place across Australia, showing off the Para-sport disciplines of swimming, boccia, table tennis, basketball, cycling, athletics and goalball. Check out the Australian Paralympic website for details. | Two articles on the same subject | http://www.coca-colajourney.com.au/coke-in-the-community/breaking-down-the-barriers http://www.coca-colajourney.com.au/coke-in-the-community/two-years-to-rio-paralympians-are-getting-involved-ahead-of-2016-paralympic-games |
| A75 | Industry | Constituency building | Seek involvement in the community | Along with curricular support, the Galilee School takes a ‘holistic approach’ to a young person’s education. With the support of a Coca-Cola Australia Foundation grant, the school picks the kids up in the morning, provides all their daily meals and drops them off at the end of the day. |  | http://www.coca-colajourney.com.au/coke-in-the-community/doors-opening-at-galilee-short-film-shows-off-school |
| A76 | Industry | Constituency building | Seek involvement in the community | Ride2Work Day is only one of many initiatives Bicycle Network employs to get people onto their bikes. The Happiness Cycle, a joint project with Coca-Cola, encourages kids to get moving by donating thousands of bikes in towns across the country. |  | http://www.coca-colajourney.com.au/stories/bikes-for-breakfast-why-riding-to-work-is-a-great-start-to-the-day |
| A77 | Industry | Constituency building | Seek involvement in the community | With the help of supporters like the Coca-Cola Australia Foundation, by 2018, AIME will annually engage 10,000 Indigenous high school students across Australia. Our goal is to have Indigenous students transitioning into university, employment or further training at the same rate as every Australian child. And, with the amount of young Indigenous talent out there, it’s well within our reach. |  | http://www.coca-colajourney.com.au/coke-in-the-community/finding-the-talent-you-never-knew-you-had-aime-gets-students-juggling |
| A78 | Industry | Constituency building | Seek involvement in the community | Before Nishant joined Coca-Cola South Pacific, he took part in the Enactus program at the University of Sydney. Since graduating, he’s worked as a mentor for current students. This year, the group he’s advising have been selected to represent Australia at the Enactus World Cup in Beijing - the first time a local university has ever been through to the finals. [...] “Especially since the financial crisis **a lot of governments have a lot less money to spend on community outreach**, social enterprises have been a really good alternative. So I think that it’s a really effective avenue that they can still continue to do the work that they do.” [...] Nishant agrees that there’s an opportunity there for business to solve entrenched social problems. “**There are a lot of social needs out there but they’re not being solved by government**,” he said. “We can wait and lobby, but t**here’s a business opportunity there to tackle that need, make money out of it** - and essentially do good in the community.” |  | http://www.coca-colajourney.com.au/stories/doing-good-is-good-for-business-enactus-profits-disadvantaged |
| A79 | Industry | Constituency building | Seek involvement in the community | “We really want to help the young people to develop confidence for their future,” explained Mission Australia CEO, Catherine Yeomans.  “We’ve been working with the community on Mornington Island for a number of years now, but it’s an exciting new initiative we’re working on with the Coca-Cola Australia Foundation.” |  | http://www.coca-colajourney.com.au/coke-in-the-community/the-tree-of-life-is-growing-on-mornington-island-mission-australia-gives-kids-confidence |
| A80 | Industry | Constituency building | Seek involvement in the community | Ronald McDonald House Charities Australia - our supporters: Coca Cola |  | https://www.rmhc.org.au/about-us/our-supporters |
| A81 | Industry | Constituency building | Seek involvement in the community | "CCAF, an independent charitable trust funded by the Coca-Cola System in Australia, supports organisations working to improve the lives of marginalised young Australians. Each year through its grant program, the Foundation distributes $1.1 million to programs assisting disadvantaged young people. "" | Coca Amatil | http://ccamatil.com/AboutCCA/Pages/Coca-ColaFoundation.aspx |
| A82 | Industry | Constituency building | Seek involvement in the community | "Every year, the Coca-Cola Australia Foundation awards a series of community Grants to local organisations throughout Australia. Up to 40 grants are awarded that vary in size from $1,000 to $10,000." "Since our inception in 1984, The Coca-Cola Foundation has given back more than $650 million to enhance the sustainability of local communities worldwide.  The Coca-Cola Foundation is our Company's primary international philanthropic arm. (...) The Coca-Cola Company is committed to giving back 1 percent of its prior year’s operating income annually. This commitment is made through The Coca-Cola Foundation and Company donations. In 2013, The Coca-Cola Company and The Coca-Cola Foundation invested more than $143 million in communities worldwide. " |  | http://www.coca-colajourney.com.au/coca-cola-australia-foundation/community-grants http://www.coca-colacompany.com/our-company/the-coca-cola-foundation |
| A83 | Industry | Constituency building | Seek involvement in the community | "The Coca-Cola Australia Foundation is proud to be working with the following national partners providing support and inspiration for marginalised young Australians:  Australian Indigenous Mentoring Experience   The Beacon Foundation  The Clontarf Foundation " |  | http://www.coca-colajourney.com.au/coca-cola-australia-foundation/our-partners |
| A84 | Industry | Constituency building | Seek involvement in the community | "Coca-Cola in Australia and its employees donated generously to the fire ravaged state of Victoria in the wake of the devastating bush fires, providing cash donations, beverage and food products. The Coca-Cola Australia Foundation also offered a community grant to the one of the worst affected areas to help with the rebuilding. " |  | http://www.coca-colajourney.com.au/coca-cola-australia-foundation/disaster-relief-and-recovery |
| A85 | Industry | Constituency building | Seek involvement in the community | "Coca-Cola Australia Foundation's mission is to make a tangible difference in the lives of disadvantaged young Australians.” |  | http://www.coca-colajourney.com.au/coke-in-the-community/ccaf-annual-report |
| A86 | News | Constituency building | Seek involvement in the community | "A program which began in 2005 to help provide corporate clothing, and later resume and interview training, Fitted for Work, is now booming and last month celebrated having helped 16,000 women enter the jobs market. [...]The Coca Cola Australia Foundation has been a major supporter of Fitted for Work over the past two years, donating $115,000 to two of its programs, which have included teaching presentation skills." |  | http://www.theaustralian.com.au/business/work-venture-for-marginalised-women-a-roaring-success/story-fn717l4s-1227079194064?nk=795c3e22ee8382de5706ae80c6db036b |
| A87 | News | Constituency building | Seek involvement in the community | A SOFT drink maker giving away 300 bicycles to teenagers in Orange sounds a bit like a corporation trying to ease its conscience, but other communities which have already experienced the Coca Cola-sponsored Happiness Cycle have seen some positives come out of the program. |  | http://www.centralwesterndaily.com.au/story/2630453/our-say-bid-to-shift-focus-towards-fitness/ |
| A88 | News | Constituency building | Seek involvement in the community | "As part of its global commitment to improving water quality, the Coca-Cola Foundation working with environmental not for profit, Landcare Australia, offered to help fund and support the distribution of rice straw to those affected by bushfire. Sonia admitted she was a little surprised by the show of support which came from Landcare Australia and backed by Coca Cola Foundation." |  | http://www.coca-colajourney.com.au/coke-in-the-community/straw-and-more-how-landcare-is-saving-rivers |
| A89 | News | Constituency building | Seek involvement in the community | "Four thousand kilometres might seem like a long way to go for lunch, but the ten boys who just made the journey from the southern most tip of Western Australia to Sydney to share a meal at Coca-Cola Place, will go much further than anyone ever imagined. The boys are all participants of the Clontarf Foundation, an organisation which launched in 2000 with the stated goal of improving the academic outcomes of boys from indigenous backgrounds. From humble beginnings the Clontarf Academy now operates in 59 schools around Australia, and is assisting more than 3000 boys, thanks to its many supporters, including the Coca-Cola Australia Foundation. " | Kids wearing red T-shirts - but might not be Coca Cola | http://www.coca-colajourney.com.au/coke-in-the-community/clontarf-students-are-rewriting-their-future |
| A90 | News | Constituency building | Seek involvement in the community | Stacy’s New School “Our dream and our goal is that each child graduate from Giant Steps and move to a mainstream school,” Michelle explained. “They’ll find it challenging to go to a class, so inclusion is one of our big goals. Just because a child has autism doesn’t mean they don’t deserve the same opportunities as any other child.” The program, **which is supported by the Coca-Cola Foundation,** has been an unqualified success. This year, nine students are moving from Giant Steps into mainstream schools. And Stacy is one of them. “I spoke to her mum the other day, and she’s doing really, really well,” said Michelle. “She goes to assembly with the entire school, goes to some classes with the other students, and spends her break with the children. It’s a great outcome.” |  | http://www.coca-colajourney.com.au/coke-in-the-community/autism-how-one-program-is-taking-giant-steps |
| A91 | News | Constituency building | Seek involvement in the community | The Coca-Cola Australia Foundation (CCAF) is calling on applications for its 2015 Community Grants Program – closing Friday March 27 – from local Community groups and programs with DGR status, that aim to make a positive difference in the lives of marginalised young Australians. The CCAF awards up to 40 grants a year ranging from $1,000 to $10,000 each for successful applicants. Since its inception, over $10 million has been awarded to hundreds of programs across Australia. Julie White, CCAF chairwoman, encourages all Community groups to apply for a grant, explaining that: “The Coca-Cola Australia Foundation Grants are a great opportunity for local Community organisations to receive financial assistance to continue the positive impact they make on marginalised youth in the Community.” “Since 2002,” adds White, “these grants have provided valuable support to Community organisations assisting marginalised youth. We are looking forward to supporting the next round of grantees and encourage all organisations that support marginalised youth to apply.” |  | http://www.fpmagazine.com.au/2-4-million-in-grants-up-for-grabs-340654/ |
| A92 | Twitter | Constituency building | Seek involvement in the community | [Duplicate] The Salvation Army ‏@salvos  Special guests Cate Blanchett & David Gonski AC speaking with @sallyloane at the Sydney #RedShieldAppeal launch. |  | https://twitter.com/salvos/status/461709607669227521/photo/1 |
| A93 | Twitter | Constituency building | Seek involvement in the community | Coca-Cola Australia retweeted Bicycle Network @bicycle_network · Sep 16 Teens are working together, problem solving and sharing tools to get their #HappinessCycle bikes ready to roll |  | https://twitter.com/CocaColaAU |
| A94 | Twitter | Constituency building | Seek involvement in the community | [Duplicate] Coca-Cola Australia ‏@CocaColaAU Our street art program helps to inspire creativity among Australian youths: http://CokeURL.com/gcy6j |  | https://twitter.com/CocaColaAU/status/516354011663179777 |
| A95 | Twitter | Constituency building | Seek involvement in the community | Coca-Cola Australia ‏@CocaColaAU From breaky to a better life - Opening up a conversation on homelessness in Australia: http://CokeURL.com/38utx |  | https://twitter.com/CocaColaAU/status/517569489811275776 |
| A96 | Twitter | Constituency building | Seek involvement in the community | Coca-Cola Australia ‏@CocaColaAU Teenagers learn about leadership with the #HappinessCycle http://CokeURL.com/u2pvp | Happiness Cycle | https://twitter.com/CocaColaAU/status/521412451112603648 |
| A97 | Twitter | Constituency building | Seek involvement in the community | Coca-Cola Australia ‏@CocaColaAU Tour de Cure has helped raise over $14m for cancer research, support and prevention programs since 2007! http://CokeURL.com/abvyk | Story about Coca's employees (picture of one employee with a Coke jersey during the Tour), but no evidence of Coca involvement Retweeted on 19 Oct 2014 | https://twitter.com/CocaColaAU/status/520446108162863104 |
| A98 | Twitter | Constituency building | Seek involvement in the community | [Duplicate] Coca-Cola Australia ‏@CocaColaAU Marist Youth Care Centre helps young people through the basics of finding a job: http://CokeURL.com/vckhc |  | https://twitter.com/CocaColaAU/status/519600323107622912 |
| A99 | Twitter | Constituency building | Seek involvement in the community | [Duplicate] Coca-Cola Amatil ‏@CocaColaAmatil The Coca-Cola Australia Foundation is a major supporter of 'Fitted for Work' and the fantastic work they do. |  | https://twitter.com/CocaColaAmatil/status/519613966612299779 |
| A100 | Twitter | Constituency building | Seek involvement in the community | Coca-Cola Australia ‏@CocaColaAU We're joining @RED to #ShareTheSound of an #AIDSFREEGEN. Help us get the message out & #RT this vid: http://CokeURL.com/z5ggw  [duplicate other weeks, same campaign] Coca-Cola Australia ‏@CocaColaAU Join @OneRepublic on the road & get VIP access at their concert by helping fight AIDS with @RED #ShareTheSound - 20 Nov 2014 Hear how you could have a chance to live the ultimate year in music with @avicii thanks to @RED! #ShareTheSound - 27 Nov 2014 Join us to support an AIDS free generation this #WorldAIDSDay - 30 Nov | Lot of negative comments from the public Link to different videos for the RED project (HIV) on Youtube with plenty of advertising for Coke https://www.youtube.com/watch?v=foc0WiujVKk&index=5&list=UU2rioA4YYH4kv2PQOXnpW7Q https://www.youtube.com/watch?v=foc0WiujVKk&index=5&list=UU2rioA4YYH4kv2PQOXnpW7Q http://www.omaze.com/experiences/bono | https://twitter.com/CocaColaAU/status/529772080212877313 |
| A101 | Twitter | Constituency building | Seek involvement in the community | Coca-Cola Australia @CocaColaAU · 4h 4 hours ago  Cana Communities, supported by the Coca-Cola Foundation, share top gardening tips: http://CokeURL.com/adal7 | Then link to Coca's website - already captured in 2014 | https://twitter.com/CocaColaAU/status/564929206648586240 |
| A102 | Twitter | Constituency building | Seek involvement in the community | Nicki Drinkwater @NickiDrinkwater · Feb 27  Great to see @CocaColaAU @CocaColaAmatil supporting country rugby 7s. #Kiama7s @WINTV1 @illawarramerc @wavefmnewsroom | The person is employed by Coca Cola Amatil | https://twitter.com/NickiDrinkwater/status/571564401393729536 |
| A103 | Uni | Constituency building | Seek involvement in the community | Information about the project provided from the University of South Australia (response to our request) - Here, information from Coca's website: Dr Edoardo Rosso is Research Fellow and head of the University of South Australia’s Football United Program – a project aimed at bringing communities together through soccer. Football United is a wider national program developed by Dr Anne Bunde Birouste from the University of NSW. In South Australia, Dr Rosso saw an opportunity to get kids further involved in their community. All he needed was some financial support and that’s where Coca-Cola Australia stepped in. | Information provided by University of South Australia | http://www.coca-colajourney.com.au/kicking-goals-how-a-new-soccer-program-is-helping-refugee-kids |
| A104 | PH | Constituency building | Establish relationships with key opinion leaders and health organisations | Sports Dietitians of Australia - Our Partners: Gatorade | And then have Fact sheets on sport drinks  Also at least one dietitian works for Coca Cola http://www.sportsdietitians.com.au/findasportsdietitian/1467/BobbieCrothers/287 | http://www.sportsdietitians.com.au/partners/ |
| A105 | Political party | Financial incentive | Financial incentives | Political Party Disclosure Return FINANCIALYEAR 2011-12 Australian Labor Party Name: Coca-Cola Amatil Limited $27,500 |  | http://periodicdisclosures.aec.gov.au/Returns/49/PNGX9.pdf |
| A106 | Political party | Financial incentive | Financial incentives | Political Party Disclosure Return FINANCIAL YEAR 2011-12 Australian Liberal Party Name: Coca-Cola Amatil Limited $27,500 |  | http://periodicdisclosures.aec.gov.au/Returns/49/PQDN1.pdf |
| A107 | Political party | Financial incentive | Financial incentives | Political Party Disclosure Return FINANCIAL YEAR 2012-13 Australian Labor Party Name: Coca-Cola Amatil Limited $27,500 |  | http://periodicdisclosures.aec.gov.au/Returns/51/RANF1.pdf |
| A108 | Political party | Financial incentive | Financial incentives | Political Party Disclosure Return FINANCIAL YEAR 2013-14 Australian Labor Party Name: Coca Cola Amatil (Sally Loane) $55,000 |  | http://periodicdisclosures.aec.gov.au/Returns/55/SLDJ8.pdf |
| A109 | Political party | Financial incentive | Financial incentives | Political Party Disclosure Return FINANCIAL YEAR 2013-14 Liberal Party of Australia Name: Coca Cola Amatil $55,000 |  | http://periodicdisclosures.aec.gov.au/Returns/55/SLCE8.pdf |
| A110 | Industry | Information and messaging | Stress the economic importance of the industry | "(…) CCA: Employs more than 15,000 people and has access to more than 265 million consumers through over 700,000 active customers" | Coca Amatil | http://cokegrads.com.au/about-cca |
| A111 | Charity | Information and messaging | Frame the debate on diet- and public health-related issues | The Happiness Cycle is a new program from Coca-Cola in partnership with Bicycle Network "The nation-wide program saw Coca-Cola South Pacific partner with Bicycle Network to encourage teenagers to get active for an hour a day. (…)  **The Happiness Cycle initiative is part of a wider program to help curb obesity**. “We absolutely recognise that there is an issue globally with obesity,” said Amy.  “We know that **you’ve got to balance kilojoules in with kilojoules out**. We’ll do everything we can to manage kilojoules in by offering a range of products in our portfolio, but we realised we weren’t doing enough to manage kilojoules out. **We just want to be part of the solution**.”" "“Adolescents need sixty minutes of physical activity most days to be healthy and happy, and bike riding is a great way of achieving this,” explained Bicycle Network’s General Manager for Behaviour Change, Tess Allaway." | Colors of the Happiness Cycle website: red and white | https://happinesscycle.com.au/about https://happinesscycle.com.au/contact http://www.coca-colajourney.com.au/happiness-cycle/its-about-freedom-what-weve-learned-from-happiness-cycle-2014 http://www.coca-colajourney.com.au/pedal-power-bicycle-network-and-coca-cola-invite-australians-to-get-on-their-bikes |
| A112 | Charity | Information and messaging | Frame the debate on diet- and public health-related issues | [Stopped in Sept 2014?]  Exercise is Medicine - Major Sponsor: Coca-Cola South Pacific |  | http://exerciseismedicine.org.au/ |
| A113 | Facebook | Information and messaging | Frame the debate on diet- and public health-related issues | "Do you want a Coke?"(Picture) Coca-Cola Australia - "Is this even a question COKE fans? #decisionsdecisions" Brianna Yaxley - "Full of sugar" 16 septembre, 05:09  Coca-Cola Australia - "Hey Brianna, COKE can be consumed as part of **a sensible, balanced diet. All of the nutritional information is clearly labelled** on our packs." |  | https://www.facebook.com/CocaColaAustralia/photos/a.10150195239600153.441271.119565995152/10154570481135153/?type=1&relevant_count=1 |
| A114 | Facebook | Information and messaging | Frame the debate on diet- and public health-related issues | Coca-Cola Australia - "Which comes first? The COKE, or the ice?" Rohin Chhabra - "diabetes" 5 septembre, 01:46  Coca-Cola Australia - "That is not accurate Rohin. **Diabetes occurs when the body can 1) no longer make insulin, or 2) make enough insulin, or 3) properly use insulin, a hormone produced by the pancreas**."  1 · 9 septembre, 22:24 Nataleigh Cordier - "Goes ice, coke, diabetes." 3 septembre, 22:37  Coca-Cola Australia - "HI Nataleigh, **Diabetes actually occurs when the body can 1) no longer make insulin, or 2) make enough insulin, or 3) properly use insulin, a hormone produced by the pancreas."**  11 septembre, 16:14 |  | https://www.facebook.com/CocaColaAustralia/photos/a.10150195239600153.441271.119565995152/10154532932105153/?type=1&relevant_count=1 |
| A115 | Industry | Information and messaging | Frame the debate on diet- and public health-related issues | Coca Cola Journey:  Last year we started a conversation around one of the biggest health issues to affect our society, **obesity**. As part of this we announced a number of commitments within our business to offer consumers **more choice, information and innovation, as well as encouraging Australians to get active**. We continue to make **positive changes**. Here’s just a taste of what we’ve achieved. 1. Increasing the availability of our **smaller portion sizes**. 2. Offering **more low kilojoule options** 3. Provide **transparent nutrition information in more places**. 4. Help get people moving by **supporting physical activity programs** |  | http://www.coca-colajourney.com.au/our-commitments-to-help-address-obesity/our-ongoing-commitments-to-help-address-obesity |
| A116 | Industry | Information and messaging | Frame the debate on diet- and public health-related issues | Recently, Coca-Cola attracted criticism from The Parent’s Jury for an incentive that provides sporting equipment to amateur sports clubs. Called the **Powerade Sports Loyalty Program**, there were concerns the program was designed to reach kids - but we want to set the record straight. We are always striving to be **responsible marketers**. [...] To cater for that demand, we offer beverages to all amateur sporting clubs with **members of all ages**. Part of our service is the Powerade Sports Loyalty Program, where clubs can collect points which they can exchange for equipment such as cricket balls, kicking tees, bikes and BBQs - the **kind of stuff that can make a real difference to community sport**. While it’s called the Powerade Sports Loyalty Program, clubs can earn points from any of our non-alcoholic portfolio, including juice, still and sparkling water and diet and low kilojoule options. Meanwhile, **it’s the club that’s rewarded, not the consumer**. [...] It's important to us to be **part of, and responsible to, the communities in which we operate**, and so we're always keen to hear from those communities if you have concerns. |  | http://www.coca-colajourney.com.au/stories/being-a-good-sport-our-policy-on-responsible-marketing |
| A117 | Industry | Information and messaging | Frame the debate on diet- and public health-related issues | What Exactly is a Dietitian? […] “**Recommending radical diets that cut out whole food groups can certainly be dangerous**,” said Maree. “Whether that’s dairy, bread, or cereals – we need all the different five food groups to make sure that we’re getting a **balanced diet** and covering all the nutrients that we require for healthy living.” |  | http://www.coca-colajourney.com.au/stories/what-exactly-is-a-dietitian |
| A118 | Industry | Information and messaging | Frame the debate on diet- and public health-related issues | Run for Your Life: How Zombies can Make You Fitter and Faster […] That’s the premise behind Zombies, Run!, an immersive fitness app created by London-based entertainment company Six to Start and writer Naomi Alderman. |  | http://www.coca-colajourney.com.au/stories/run-for-your-life-how-zombies-can-make-you-fitter-and-faster |
| A119 | Industry | Information and messaging | Frame the debate on diet- and public health-related issues | "Low and no-sugar beverages are a high growth part of the CCA portfolio growing at more than three times the rate of sugar-sweetened beverages in 2012." | Coca Amatil | http://ccamatil.com/AboutCCA/Pages/CompanyOverview.aspx |
| A120 | Industry | Information and messaging | Frame the debate on diet- and public health-related issues | "In 2007, the Foundation broadened its support to include global water stewardship programs, **fitness and nutrition efforts** and community recycling programs.” |  | http://www.coca-colacompany.com/our-company/the-coca-cola-foundation |
| A121 | Industry | Information and messaging | Frame the debate on diet- and public health-related issues | "Knowing the energy that foods and beverages provide can **help you to balance the energy you take in with the energy you expend** throughout the day via basic bodily functions, normal daily activities and physical activity. " "When it comes to managing your weight, it's **important to balance the kilojoules you take in with the kilojoules you burn each day**. You can do this by eating a well-balanced diet and enjoying regular physical activity. (...)  Some key things to remember when it comes to balancing energy in with energy out:  All foods and beverages have the potential to contribute excess kilojoules, including those from our beverages, so be mindful of the total quantity of kilojoules you consume  Even if your diet is nutritionally well balanced, consuming too much of any food or beverage can contribute to excess kilojoule intake and weight gain  Manage **portion sizes** and how often you consume those portions  **Moving** burns kilojoules so move more! Even simple activities like gardening, walking and doing the housework can help to burn kilojoules" "**More Information equals more informed consumers, and we believe informed consumers are the ones that make the best decisions for themselves and their families**" |  | http://www.coca-colajourney.com.au/how-to-read-the-labels-on-our-products http://www.coca-colajourney.com.au/our-products/energy-balance http://www.coca-colajourney.com.au/clear-on-kilojoules |
| A122 | News | Information and messaging | Frame the debate on diet- and public health-related issues | Happiness Cycle Hits Hobart (…) Together the local council, campaign partner Bicycle Network and Coca-Cola Australia Foundation put on two vocational skills training sessions in advance of the Happiness Cycle event day |  | http://www.coca-colajourney.com.au/happiness-cycle/happiness-cycle-hits-hobart |
| A123 | News | Information and messaging | Frame the debate on diet- and public health-related issues | On Coca Cola's website: By:  **Zoe Wilson, Accredited Practicing Dietitian** (...)  McDonalds is giving the fast food world a gourmet, high-tech facelift with their new ‘Create Your Taste’ menu. (...) However the fact that you can create your own meal or burger and you have a more a gourmet style menu to choose from doesn’t necessarily mean a healthier meal, so with my dietitian’s hat on here’s a few tips for creating your own meal at Macca’s:   Don’t go overboard, the more ingredients you add the higher you bump up the overall kilojoule count, salt and fat content.  Be sure to add salad, this will add flavour and taste without too many more kilojoules.  If you opt for a sauce keep it to one or maybe even ask for a half serve of sauce.  While you can choose the number of beef patties that make up your gourmet burger, stick with just the one it will still taste great and again help to keep kilojoules down.  And if you are managing your weight I’d suggest going to a **low kilojoule beverage like a diet soft drink or water to top it off**. McDonalds have come up with the future of fast food. It has the potential to be **an easy option for those wishing to make more health-conscious decisions**, or for those with dietary restrictions" | Coca Cola promoting McDonald's | http://www.coca-colajourney.com.au/stories/the-future-of-fast-food-creating-your-own-menu-at-mcdonalds |
| A124 | News | Information and messaging | Frame the debate on diet- and public health-related issues | The open road is calling. So are the back tracks, mountain passes and bike trails. Victoria’s got a strong claim on being the nation’s cycling capital, with beautiful rides throughout the state. Whether you’re up for some heavy-duty bush bashing or a more relaxed ride, there are routes for cyclists of every stripe. Here are a few of our favourite bicycle tours – both day-long and overnight – across the garden state. | In the "Health and Wellbeing" section | http://www.coca-colajourney.com.au/stories/happy-trails-summer-cycling-in-victoria |
| A125 | News | Information and messaging | Frame the debate on diet- and public health-related issues | A video featuring employees practising sports or playing instruments - video is called "being balanced" |  | http://www.coca-colajourney.com.au/videos/being-balanced-bc4026681158001 |
| A126 | News | Information and messaging | Frame the debate on diet- and public health-related issues | Teenagers are sophisticated, opinionated and digitally savvy. So **when it comes to encouraging teens to get active, you have to think a little outside the square** to get them on board. The Happiness Cycle is anything but square. The initiative by Coca-Cola and Bicycle Network saw over 5000 new bicycles delivered to teens across the country. Not only were high school students given their own bicycles, they were also trained to build it themselves and given tools to track their rides. The point of the Happiness Cycle was simple: inspire teens to get moving for an hour a day. |  | http://www.coca-colajourney.com.au/happiness-cycle/happiness-is-5-400-new-cyclists-how-bicycle-network-got-the-happiness-cycle-up-and-running |
| A127 | News | Information and messaging | Frame the debate on diet- and public health-related issues | Video: 5 small changes to help manage your help - eating versus physical activity "According to Jim Hill, Professor of Paediatrics and Medicine at the University of Colorado" Get a pedometer Eat breakfast "when we eat breakfast, we manage our calories" video not working |  | http://www.coca-colajourney.com.au/videos/prof-jim-hill-5-small-changes-bc3982876002001 |
| A128 | Twitter | Information and messaging | Frame the debate on diet- and public health-related issues | Coca-Cola Australia retweeted Bicycle Network @bicycle_network · Sep 16 Bikes built - let's ride! #happinesscycle |  | https://twitter.com/CocaColaAU |
| A129 | Twitter | Information and messaging | Frame the debate on diet- and public health-related issues | Coca-Cola AustraliaVerified account ‏@CocaColaAU How do you travel to work? Some of us cycle: http://CokeURL.com/xclgs | Then link to the Coca Cola Journey website with story about Bicycle Network - http://www.coca-colajourney.com.au/stories/bikes-for-breakfast-why-riding-to-work-is-a-great-start-to-the-day | <https://twitter.com/CocaColaAU/status/542060957338718208> |
| A130 | Lobby | Information and messaging | Lobby Establish relationships with policy makers | Lobby Register Endeavour Consulting Group Pty Ltd and Hill and Knowlton Client: Coca Cola South Pacific |  | http://lobbyists.pmc.gov.au/export/export_client.cfm |
| A131 | Conference | Information and messaging | Shape the evidence base on diet- and public health-related issues the evidence base on diet- and public health-related issues evidence | Dietitians Association of Australia 31st National Conference - Exhibitors: Coca-Cola South Pacific |  | http://arinex.com.au/dietitians2014/sponsorship-exhibition/ |
| A132 | Conference | Information and messaging | Shape the evidence base on diet- and public health-related issues evidence | The Nutrition Society of Australia 2014 Annual Scientific Meeting - Major Sponsor and Breakfast sponsor: Coca Cola |  | http://www.nsa.asn.au/index.php/2014_ASM/general_information/ |
| A133 | Conference | Information and messaging | Shape the evidence base on diet- and public health-related issues evidence | Wednesday 26 November 2014 - 2014 Nutrition Society of Australia Annual Scientific Meeting Do small changes make a big difference? Insights into weight loss maintenance research. Presenter: Professor James Hill, Denver University, USA | Sponsored by Coca Cola | http://www.nsa.asn.au/index.php/2014_ASM/breakfast_sessions/ |
| A134 | Industry | Information and messaging | Shape the evidence base on diet- and public health-related issues evidence | While they contribute minimal kilojoules to the diet, people question the role of diet soft drinks when managing their weight. [...] A new study funded by the American Beverage Association and published in the journal Obesity may just have provided evidence to suggest otherwise. The study, conducted by researchers from the University of Colorado and Temple University, investigated the impact of low- and no-kilojoule beverages on weight loss among overweight and obese men and women. |  | http://www.coca-colajourney.com.au/stories/study-shows-diet-drinks-can-be-part-of-weight-loss-plans |
| A135 | Industry | Information and messaging | Shape the evidence base on diet- and public health-related issues evidence | As part of our commitment to providing Australians with more low-kilojoule beverage options we understand there are sometimes questions around sweeteners. Here’s a look at what the science says. | Then provides infographics with a mix of evidence from professionals bodies or international organisations (not all evidence is peer reviewed evidence) and at least 3/20 from the food industry or front groups (not exhaustive): - Ref 6 "The Use of Low-Calorie Sweeteners by Adults: Impact on Weight Management" Supported by the Committee on Low-Calorie Sweeteners of the North American Branch of the International Life Sciences Institute. G. H. Anderson and J. Foreyt received a modest honorarium for their participation in the workshop and development of the manuscript. All authors received travel funding to attend the workshop. http://jn.nutrition.org/content/142/6/1163s.full - Ref 8 The Calorie Control Council, established in 1966, is an international association representing the low- and reduced-calorie food and beverage industry. http://www.caloriecontrol.org/about-the-council - Ref 15: authors from the Burdock Group (working with food companies) http://informahealthcare.com/doi/pdf/10.1080/10408440701516184 - Ref 16: authors from The NutraSweet Company, Mt. Prospect, Illinois http://www.sciencedirect.com/science/article/pii/S0273230002915424 | http://www.coca-colajourney.com.au/stories/sweetener-questions-answered |
| A136 | Industry | Information and messaging | Shape the evidence base on diet- and public health-related issues evidence | "Concerns have been growing regarding the role of fructose-containing dietary sugars and their food and beverage sources in the development of obesity and cardio-metabolic disease. On the 2nd December, 2013, a Symposium held in Sydney, Australia brought together international and local experts to explore the current evidence on fructose-containing sugars and sugar-sweetened beverage trends, discussing these issues in the context of the changing landscape." |  | http://www.coca-colajourney.com.au/health-professionals/sweet-symposium-webinar-a-spotlight-on-fructose-sugar-sweetened-beverage-trends |
| A137 | Industry | Information and messaging | Shape the evidence base on diet- and public health-related issues evidence | "Quick Guide to Low and No Kilojoule Sweeteners - As part of our commitment to providing Australians with more low-kilojoule beverage options we understand there is some confusion surrounding the low-kilojoule sweeteners used in our drinks. That's why we have developed this summary brochure to assist healthcare professionals in answering common questions you may receive from your patients." |  | http://www.coca-colajourney.com.au/health-professionals/quick-guide-to-low-and-no-kilojoule-sweeteners |
| A138 | Industry | Information and messaging | Shape the evidence base on diet- and public health-related issues evidence | "The Prescription is Exercise Webinar What if there was one medicine so powerful in maintaining and improving health that it could help prevent or treat many diseases, such as diabetes, hypertension, heart disease and obesity? [1] Scientific studies show that regular exercise can reduce the risk of more than 20 illnesses and is a useful treatment for type 2 diabetes, hypertension, depression, and other conditions. [2] Exercise is Medicine Australia is an initiative designed to help improve the health and well-being of the nation through a regular physical activity prescription from doctors and other health care providers. Join us in this free webinar to hear from international guest speaker, Professor Steven Blair, one of the world's leading exercise scientists from the University of South Carolina, as he provides a global overview of Exercise is Medicine and its benefits to health and the prevention and treatment of many chronic diseases. Providing a local perspective including its current status in Australia will be Professor Robert Newton, from the Edith Cowan University Health and Wellness Institute." | "Exercice is Medicine" was funded by Coca Cola  Steven Blair received a grant from Coca Cola | http://www.coca-colajourney.com.au/webinar-the-prescription-is-exercise |
| A139 | Industry | Information and messaging | Shape the evidence base on diet- and public health-related issues evidence | "Coca-Cola Australia has an advisory council of experts in the area of obesity, public health and nutrition, who provide advice and counsel to the Company. In 2010 the Council reviewed the latest research on the sweeteners we use in our beverages. The review found that these sweeteners are acceptable and safe for use and that they can also play a beneficial role in the diet of people with diabetes or people interested in managing their weight" |  | http://www.coca-colajourney.com.au/position-statement-non-nutritive-sweeteners-aspartame-acesulphame-k-and-stevia http://assets.coca-colacompany.com/39/e7/dc387d3d47dfb6a17cf66844d102/position-statement-on-the-non-nutritive-sweeteners-aspartame-acesulphame-k-and-stevia.pdf |
| A140 | Government | Policy substitution | Policy substitution | Food and Health Dialogue - Industry roundtable participants include: SPC Ardmona |  | http://www.foodhealthdialogue.gov.au/internet/foodandhealth/publishing.nsf/Content/about-us |
| Non coded | Government |  | Free code | 44th Parliament: Members' Interest Statements MP D Coleman: Shareholding: Coca Cola Amatil Ltd |  | http://www.aph.gov.au/~/media/03%20Senators%20and%20Members/32%20Members/Register/44p/CF/ColemanD_44P.pdf |

## McDonald’s

| **Reference in manuscript** | **Source** | **Strategy** | **Practice (code used for analysis)** | **Data coded** | **Notes** | **Website URL** |
| --- | --- | --- | --- | --- | --- | --- |
| A141 | Charity | Constituency building | Seek involvement in the community | At Macca’s we’re proud to play an active role in neighbourhoods all over Australia. One of the ways we do this is by supporting groups and charities that are important to our customers or that make a difference to the lives of our fellow Australians. At the heart of our community commitment is Ronald McDonald House Charities (RMHC). RMHC is one of Australia’s major children’s charities and helps thousands of seriously ill children and their families every year. To raise funds for RMHC, McHappy Day is held annually and everyone in the McDonald’s business – franchisees, crew, managers, head office staff and suppliers – participates in some way. Celebrities and local personalities join in the fun and **two dollars from every Big Mac® sold is donated to RMHC**. Other products and merchandise are also sold to contribute to the fundraising. |  | http://www.rmhc.org.au/ |
| A142 | Facebook | Constituency building | Seek involvement in the community | McDonald's Australia Facebook post:  17 october We're proud to have Ryan Kwanten support Ronald McDonald House Charities Australia on #McHappyDay. You can catch him at McDonald's Darling Quarter between 12pm and 2pm today! "Today, at noon, I will be at the McDonalds in Darling Harbour. **Please come down and share a Big Mac** with the #McHappyDay team…" (original post from Ryan Kwanten) | Also changed its profile picture https://www.facebook.com/McDonaldsAU/photos/a.357069394336000.77558.179866158722992/796564243719844/?type=1 | https://www.facebook.com/McDonaldsAU/posts/797435676966034 |
| A143 | Industry | Constituency building | Seek involvement in the community | Macca's Grassroots is a new initiative in Western Australia which provides local **sporting clubs**, **community** groups and charitable organisations the opportunity to host a fundraising day at their local participating McDonald’s restaurant. (Western Australia) |  | https://mcdonalds.com.au/grassrootswa/about |
| A144 | Industry | Constituency building | Seek involvement in the community | Mac Pack Basketball Super Clinic | "Check out these pics to see how much fun participants had with NBA superstar Andrew Bogut at this year’s Clinic!" - Pictures with kids wearing red and yellow equipments - Ronald McDonald's playing with them | https://mcdonalds.com.au/mac-pack/champions-of-play/basketball |
| A145 | Industry | Constituency building | Seek involvement in the community | McDonald's **community** Cinemas: We're Perth's community outdoor cinema group screening blockbuster films, classics and all your favourite movies throughout Perth's Summer months. Run by over 700 volunteers, now with five outdoor movie venues, we proudly donate all profits to kids charities. To date we've raised $5.25 million for kids in a health or physical crisis. | McDonald's logo | https:// communitycinemas.com.au/UniqueContent/cinemaselect.htm |
| A146 | Industry | Constituency building | Seek involvement in the community | "We take seriously the responsibilities that come with being a leader. We help our customers build better **communities**, support RMHC®, and leverage our size, scope and resources to help make the world a better place. " |  | https://mcdonalds.com.au/learn/careers/maccas-core-values |
| A147 | Industry | Constituency building | Seek involvement in the community | "The Charlie Bell Scholarship for Future Leaders will reward successful candidates with a contribution of up to $15,000 towards tuition fees for their chosen undergraduate or post graduate study." |  | https://mcdonalds.com.au/charlie-bell-scholarship |
| A148 | Industry | Constituency building | Seek involvement in the community | Little Athletics WA partners: McDonald's |  | http://www.walittleathletics.com.au/site/index.cfm?fuseaction=display_main&OrgID=3670 |
| A149 | Industry | Constituency building | Seek involvement in the community | Swimming Queensland sponsors: McDonald's |  | http://qld.swimming.org.au/page.php?id=69  https://mcdonalds.com.au/learn/responsibility/maccas-community/the-next-steps |
| A150 | Industry | Constituency building | Seek involvement in the community | South Australian National Football League - Macca’s Cup – the SANFL’s Under 18s competition "**Giving kids every opportunity to have fun and succeed in sport** is a passion that both McDonald’s and the South Australian National Football League share" |  | http://www.sanfl.com.au/maccas_cup/ |
| A151 | Industry | Constituency building | Seek involvement in the community | McDonald's WA is partnering with the Association for the Blind of WA to assist in the training of four Guide Dogs and developing a library of audio described DVDs. |  | http://mgjst.com.au/macca-s-in-the-wa-community.html |
| A152 | Industry | Constituency building | Seek involvement in the community | McDonald's WA has been a proud supporter of Telethon for many years. Each year all 64 McDonald's family restaurants in WA support Telethon by holding promotions in store to boost donations. |  | http://mgjst.com.au/macca-s-in-the-wa-community.html |
| A153 | Industry | Constituency building | Seek involvement in the community | McDonald's WA sponsors the annual Salvation Army Youth Camp to ensure this valuable program can continue. The Youth Camps provide 50 young West Australians from disadvantaged backgrounds the chance to overcome life issues and open them up to a world of opportunity. |  | http://mgjst.com.au/macca-s-in-the-wa- community.html |
| A154 | Industry | Constituency building | Seek involvement in the community | McDonald's is a Founding Partner of Clean up Australia. |  | http://mgjst.com.au/macca-s-in-the-wa-community.html |
| A155 | Industry | Constituency building | Seek involvement in the community | McDonald's has been a supporter of Earth Hour since its inception in 2007. All of the 64 McDonald's family restaurants in WA support this initiative by turning off their famous Golden Arches. |  | http://mgjst.com.au/macca-s-in-the-wa- community.html |
| A156 | Industry | Constituency building | Seek involvement in the community | McDonald's WA Restaurants are supporting the Cystic Fibrosis fundraising event called "Great Strides". These fundraising events combined with corporate support ensure that Cystic Fibrosis WA can continue to provide a range of support and services. |  | http://mgjst.com.au/macca-s-in-the-wa- community.html |
| A157 | Industry | Constituency building | Seek involvement in the community | Lifeline WA delivers services that aim to prevent suicide, support people in crisis and create opportunities for emotional wellbeing. One such service is a 24/7 telephone crisis support service. To encourage people to keep the Lifeline contact details with them at all times, McDonald's have provided an exclusive ongoing offer that is printed on the back of the Lifeline cards. |  | http://mgjst.com.au/macca-s-in-the-wa- |
| A158 | Industry | Constituency building | Seek involvement in the community | Ronald McDonald learning program: "As teachers, it's important to consider the whole of the child's wellbeing when planning for the student - socially, emotionally, cognitively and physically. Our EDMed Professional Development provides teachers with information and educational strategies that can be applied to children with a diverse range of illnesses." "Please find below presentations and photographs from the recent 2011 Inaugural **H.E.L.P. Conference** (Health. Educators. Learners. Practitioners) held in Sydney on the 5th & 6th September." |  | https://learningprogram.rmhc.org.au/about/edmed-professional-development.php |
| A159 | Industry | Constituency building | Seek involvement in the community | The RMHC Cord Blood Bank Program funds the collection and storage of umbilical cord blood |  | https://learningprogram.rmhc.org.au/docs/rmhc-school-kit.pdf |
| A160 | Industry | Constituency building | Seek involvement in the community | The Ronald McDonald Learning Program provides educational support to help students with serious illness catch up on missed schooling. Silly Socks 4 Sick Kids is an easy and fun way to raise funds with a gold coin donation for events such as;  • Silly Socks Free Dress Day  • Silly Socks Fashion Parade  • Silly Socks Walkathon  • Silly Socks Disco  • Silly Socks Sports Day |  | https://learningprogram.rmhc.org.au/help-out/silly-socks.php |
| A161 | Industry | Constituency building | Seek involvement in the community | Our Ronald McDonald® Family Room program gives families visiting a sick child a comfortable haven where they can take a break and relax during their visit. Here, we are able to offer a welcoming environment for the family, with the space and facilities to make everyday life caring for a sick child just that little bit easier. We currently have six Family Rooms in Australia, and we have plans to open more than 15 in the coming years. |  | https://www.rmhc.org.au/sites/default/files/Family%20Room%20Brochure-2.pdf https://www.rmhc.org.au/our-programs/family-rooms |
| A162 | Industry | Constituency building | Seek involvement in the community | There are currently six Ronald McDonald Family Retreats in Australia. Whether a cottage, beach house or country retreat, each Family Retreat is a holiday home kitted out with everything a family needs to spend a relaxing week together. We are very fortunate to have the support of local volunteers who help with running, cleaning and maintenance of each Retreat |  | https://www.rmhc.org.au/our-programs/family-retreats https://www.rmhc.org.au/sites/default/files/Family%20Retreat%20Brochure_0.pdf |
| A163 | Industry | Constituency building | Seek involvement in the community | Living in rural or remote NSW can mean families have to make long, tiring and often expensive journeys so their child can get their health check-ups from city hospitals. To lessen the burden, Ronald McDonald House Charities has partnered with Royal Far West to launch the first Australian Ronald McDonald Care Mobile to bring healthcare to rural & remote areas of NSW. |  | https://www.rmhc.org.au/our-programs/care-mobile |
| A164 | News | Constituency building | Seek involvement in the community | As the McHappy Day countdown begins, Bundaberg residents are being encouraged to purchase a $1 Helping Hand at their local McDonald's restaurant to help raise much-needed funds for Ronald McDonald House Charities (RMHC). |  | http://www.news-mail.com.au/news/call-locals-make-donation-ronald-mcdonald-house/2391046/ |
| A165 | News | Constituency building | Seek involvement in the community | "The inclusion of a McDonalds at the new Monash Children’s Hospital would create a misleading “health halo” around the fast food chain, a leading health expert says. Melbourne University professor of public health Rob Moodie said he was fearful that the new hospital, which is set to open in 2017, would include a McDonalds. Monash Children’s benefits from the Ronald McDonald House charity, as does the Royal Children’s Hospital, which controversially opened a restaurant on its site in 2009." |  | http://www.heraldsun.com.au/leader/east/health-expert-concerned-monash-childrens-hospital-will-include-mcdonalds/story-fngnvlxu-1227169451293?nk=fafccdbcb7e6f0f67bd6f03ac038eb26 |
| A166 | PR | Constituency building | Seek involvement in the community | Mc Happy Day - One of the country’s biggest **national publicity campaigns** involving a high profile celebrity ambassadors program, a state media program to support every individual restaurant, and an internal communications program to boost crew engagement and local fundraising at over 850 McDonald’s restaurants. |  | http://ppr.com.au/portfolio-item/mchappy-day/ |
| A167 | Government | Constituency building | Establish relationships with policy makers | The following organisations form membership of the Dialogue: (Food and Health Dialogue): McDonald's Australia |  | http://www.foodhealthdialogue.gov.au/internet/foodandhealth/publishing.nsf/Content/about-us |
| A168 | Industry | Information and messaging | Stress the economic importance of the industry | "Sure we're here to make dollars, but that's what keeps 90,000 of us in a job." "Employer of choice for women" "A responsible employer of students" "Indigenous employment" |  | https://mcdonalds.com.au/learn/careers/maccas-core-values |
| A169 | Industry | Information and messaging | Frame the debate on diet- and public health-related issues the debate on diet- and public health-related issues | Mac Pack: a sporting movement for kids promoting healthy living through fun and play (Victoria) Weekly Mac Pack Wrap: champions of play on the Challenge Leader Board | A few dedicated webpages - McDonad's colours (red and yellow) | https://mcdonalds.com.au/mac-pack |
| A170 | Industry | Information and messaging | Frame the debate on diet- and public health-related issues the debate on diet- and public health-related issues | "**You are what you eat**, and you have a right to know what’s in your food.  To **help you make the best choices for you and your family** we’ve put together some easy-access nutrition information about our standard menu items." "We're committed to help our customers confidently **make healthier choices** when visiting our restaurants." "Happy Meal Choices menu, which enables **parents and children to select meal components to suit individual tastes and dietary requirements**" |  | https://mcdonalds.com.au/maccas-food/nutrition |
| A171 | Industry | Information and messaging | Frame the debate on diet- and public health-related issues the debate on diet- and public health-related issues | The McDonald's GWN7 Junior Sports Trust: "So, if your school or club is in regional Western Australia and requires equipment, uniforms or something else that **encourages participation in sport**, we are happy to help."  "Now in its sixth year, the MGJST gives clubs and schools the opportunity to share in $60,000 worth of grants each year. Over the past six years MGJST has granted over $270,000." | A dedicated website with McDonald's colours | http://www.mgjst.com.au/ |
| A172 | Industry | Information and messaging | Frame the debate on diet- and public health-related issues the debate on diet- and public health-related issues | "Today **we like to focus on activities that improve the health and wellbeing of Australian children**. We contribute to sporting organisations that develop kids’ sports skills and provide opportunities for them to play. Hundreds of sporting clubs across Australia benefit from the support of their local McDonald’s restaurant and in some states restaurants work together to support state-wide programs." "What we're looking for: We want to deliver valuable benefits to the wider **community.** We want to help children live a more **balanced, active and enriched lifestyle**. Our sponsorships must reflect a **grassroots and community focus**." ""Providing customers with **safe food** is our first priority and our most critical responsibility. " | A kid with a bathing cap with McDonald's colours | https://mcdonalds.com.au/learn/responsibility/maccas-community https://mcdonalds.com.au/about-maccas/quality-and-sustainability |
| A173 | Lobby | Information and messaging | Lobby Establish relationships with policy makers | Lobby Register Professional Public Relations Pty Ltd (PPR) & Barton Deakin Client: McDonald's |  | http://lobbyists.pmc.gov.au/export/export_client.cfm |
| A174 | Conference | Information and messaging | Shape the evidence base on diet- and public health-related issues evidence | Dietitians Association of Australia 31st National Conference - Exhibitors: McDonald’s Australia |  | http://arinex.com.au/dietitians2014/sponsorship-exhibition/ |
| A175 | Industry | Information and messaging | Shape the evidence base on diet- and public health-related issues evidence | McDonald’s junior development basketball programs in partnership with Basketball Victoria - "McDonald’s Hoop Time is a series of one-day basketball round robins held at local basketball stadiums around Victoria. With a 30-year history, the program offers children in Grade 3/4 (Junior) and Grade 5/6 (Senior) the opportunity to play in a fun inter-school competition with qualified referees and receive some great prizes along the way. "  "We had many great entries and we congratulate our lucky 5 winning schools that each took home the $1,000 prize in 2013"  School resources - lessons plan (Upper Primary): o Nutrition Students develop an understanding of everyday and sometimes foods and hydration for everyday activities. Through this they begin to link the importance of healthy eating with Basketball through activities and discussion encompassing:  The five food groups   The nutrient’s they contain   **Why they are good for our body**   How much of each food group we should eat   **How each food group helps us when we play basketball**    **The importance of hydration**  "Discuss the **benefits of eating with only a small number of “Sometimes” foods included each week**." " Comment on the mix of foods – were most healthy, were there any “different” foods, does it **balance out with their energy requirements**?" o Reading Nutrition Labels Students begin to understand the importance and complexity of food labelling. It addresses product ingredients, reading the nutrition information panel, and what the claims on the front of the pack really mean.  “A **fruit based product may have a higher sugar level** because of the naturally occurring sugar content" o Healthy Lifestyle Students develop an understanding of **everyday and sometimes foods and hydration**. Additionally, they are provided with information about the importance of sleep in relation to sport. Through this they begin to link the importance of healthy eating with Basketball through activities and classroom discussion encompassing:  The five food groups   The nutrient’s they contain   **Why they are good for our body   How much of each food group we should eat   How each food group helps us when we play basketball  " Water is separate to indicate how important it is" "Do any of them have drink bottles with them at their desk? Do they sip all day? "** | A full dedicated website | http://www.hooptimebasketball.com.au/ |
| A176 | Government | Policy substitution | Policy substitution | Food and Health Dialogue - meeting May 2013 - Attended: Member from food industry - Ms Tracey Monahan: Director of Quality Insurance, ANZ, McDonald's Australia, representing the Quick Service Restaurants Forum | 8 members and 4 observers |  |
| A177 | Industry | Policy substitution | Policy substitution | (...) "In 2009 McDonald’s Australia and other Quick Service Restaurants launched the **self-regulatory** **Quick Service Restaurant Initiative for Responsible Advertising and Marketing Communications to Children**. The main principle of the Initiative states that when we are promoting food and beverages to children under 14 years we will only use foods that are healthier options. For a meal to be considered a **healthier choice it should reflect general principles of healthy eating as defined by credible nutrition authorities and must comply with defined criteria** for energy, saturated fat, sugar and sodium. **These criteria were developed by a team of Accredited Practising Dietitians** in consultation with national guidelines and authorities on children’s nutrition. As part of the joint initiative, a complaints mechanism has been established whereby consumers can make a complaint directly to the Advertising Standards Bureau providing a single speedy source for complaint resolution." | Some TV programs not covered by the initiatives (e.g., The X Factor, Futurama)  Age under 14  In-house nutritional criteria If "healthier choices", can be marketed to anyone "McDonald’s does not advertise in schools or conduct marketing activity in schools without prior approval of the school authorities. Such activity is **usually** related to educational purposes or related to healthy lifestyle or physical activity" | https://mcdonalds.com.au/about-maccas/quality-and-sustainability |
| A178 | Industry | Policy substitution | Policy substitution | "A number of menu changes have been made to improve the nutritional value of our food. For example:  Reduced sugar content in our buns to just 5%  Reduced the sodium content of our signature cheese by 20%  Happy Meal Choices menu, which enables parents and children to select meal components to suit individual tastes and dietary requirements. Choices include, 3 or 6 pack of nuggets, apple slices, French fries, garden salad, Calci-Yum flavoured low fat milk, water or apple juice" | For specific details - see Sacks et al 2014 | https://mcdonalds.com.au/about-maccas/quality-and-sustainability |
| Free code | Government |  | Free code | 44th Parliament: Members' Interest Statements MP Tony Abbott: "For over 15 years, my annual "Pollie Pedal" cycling ride has raised funds for charity. To date, over $3,763,000 has been raised for good causes including: […] Ronald McDonald House." |  | http://www.aph.gov.au/~/media/03%20Senators%20and%20Members/32%20Members/Register/44p/AB/AbbottA_44P.pdf |

## Nestle

| **Reference in manuscript** | **Source** | **Strategy** | **Practice (code used for analysis)** | **Data coded** | **Notes** | **Website URL** |
| --- | --- | --- | --- | --- | --- | --- |
| A179 | Industry | Constituency building | Seek involvement in the community | "For almost 20 years, Nestlé Australia has partnered with Cricket Australia through our MILO brand to support junior cricket development (Milo Australia). We are proud of this long-term partnership and that the number of children participating in cricket activities is increasing each year." | Kids wearing T-shirts with Milo logo | http://www.nestle.com.au/creating-shared-value/community-programs/milo-programs |
| A180 | Industry | Constituency building | Seek involvement in the community | "The Nestlé GOLDEN CHEF'S HAT AWARD is dedicated to helping junior and apprentice chefs develop their cooking skills and broaden their culinary horizons. It’s the longest running culinary competition in Australia that offers young chefs the chance to really find out where they are as a chef, by pitting themselves against their peers and benchmarking their cooking skills. Taking part in the Nestlé GOLDEN CHEF'S HAT AWARD provides young chefs with the added opportunity of being mentored by culinary instructors and given access to a national network of professional chefs. This year, the national winning team will be off to France for the world’s most prestigious cooking competition, the Bocuse d’Or World Finals, to see the best chefs in the world cook for the ultimate title." | Nestlé products are used by chefs during the competition: https://www.nestleprofessional.com/australia/en/SiteArticles/Pages/GCHAProductList.aspx | https://www.nestleprofessional.com/australia/en/CulinaryExpertise/Pages/GoldenChefsCompetitionInformation.aspx?UrlReferrer=http%3a%2f%2fwww.nestle.com.au%2fcreating-shared-value |
| A181 | Industry | Constituency building | Seek involvement in the community | "Swim Kids Operation 10,000 was a fundraising campaign developed by Uncle Tobys to help the Royal Life Saving Society raise awareness and funds to provide FREE Swim and Survive lessons for 10,000 children who would otherwise miss out." |  | http://www.uncletobys.com.au/community/royal-life-saving-society-australia/ |
| A182 | Industry | Constituency building | Seek involvement in the community | "Nestlé has had a long-standing partnership with Foodbank Australia donating unsaleable or surplus food which is still safe and nutritious to eat. (...) In 2012, Nestlé Australia donated more than 331,000kg of food to the organisation, enough to make up 442,000 meals. On top of this, as part of a collaborative initiative with the charity, our Uncle Tobys factory at Wahgunyah on the Victorian and NSW border has donated an entire day’s production of Oats porridge over the past few years." |  | http://www.nestle.com.au/creating-shared-value/community-programs/foodbank |
| A183 | Twitter | Constituency building | Seek involvement in the community | Nestle Australia ‏@nestleaunews How we’re working with the WWF in Pakistan to reduce our water footprint: http://bddy.me/1vQL5Lo |  | https://twitter.com/nestleaunews/status/538116674629545985 |
| A184 | Twitter | Constituency building | Seek involvement in the community | Nestle Australia @nestleaunews · Feb 24 More than 10,000 cases of food are on their way to Qld to help people affected by Cyclone Marcia. http://bit.ly/1BaURca | Then link to their website: http://www.nestle.com.au/media **More than 10,000 cases of food will be donated by Nestlé Australia** to those affected by Cyclone Marcia which destroyed 350 homes and left 40,000 without power last week.  Cases of **Uncle Tobys cereal, Uncle Tobys Muesli bars, Maggi noodles, Nescafé coffee and Milo powder** are currently on their way to Foodbank in Queensland, one of the charities co-ordinating the relief efforts.  It’s expected the cases of food will begin arriving in Brisbane by Friday then distributed to those in Rockhampton, Yeppoon and surrounding areas.  When it hit land, Cyclone Marcia, was rated as a category five, one of the most severe as it cut through central Queensland with the coastal towns of Yeppoon and Rockhampton worst hit. In addition to the destruction caused by the wind, many homes have been affected by widespread flooding.  As Cyclone Marcia approached Australia, **Foodbank reached out to major manufacturers seeking donations of essential items such as food, nappies and personal hygiene items**.  Foodbank said it expects relief efforts to continue for the next few weeks when the flooding subsides and the full extent of Marcia’s devastation is revealed. | https://twitter.com/nestleaunews/status/570440392438468608 |
| A185 | News | Constituency building | Seek involvement in the community | Fun at the farm for Indigenous kids Sep 24, 2014 [...]  The highly successful Nestlé volunteer program, which has been operating for the past three years in Coonamble and Gulargambone in north-west NSW, has recently formed a partnership with the Tamworth Local Aboriginal Land Council. |  | http://www.nestle.com.au/media/newsandfeatures/fun-farm-indigenous-kids |
| A186 | Industry | Constituency building | Seek involvement in the community | School Canteen Association partners Nestlé believes in the importance of **providing better food choices for children**. We have approximately 60 products Healthy Kids registered (optional as Green or Amber). |  | http://www.nestle.com.au/nhw/our-nutrition-partners |
| A187 | News | Constituency building | Seek involvement in the community | Remember playing sport as a kid? Being a brave loser, being a humble winner, kicking the winning goal, or leading the pack…and tripping over just before the finish line! **MILO is searching for kids who are the best and fairest when it comes to sport** – those kids who are good sports both on and off the field. The **MILO Valuable Player program (MVP)** was launched last December and so far hundreds of children aged 5 to 12 years have been nominated by their family, friends and sporting coaches in the MILO Wall of Fame. The program is all about celebrating the lessons kids learn through sport – all sports, boys and girls, lessons big and small. MVP also gives parents the chance to celebrate their child’s success through social sharing. Earlier this year, some of the lucky MVP’s got the opportunity to hone their skills at special cricket clinic with legends such as Australian captain and MILO Ambassador, Michael Clarke along with Holly Ferling and James Faulkner. | Kids wearing Milo T-shirts | http://www.nestle.com.au/media/newsandfeatures/who-will-be-australias-best-and-fairest-young-sporting-stars |
| A188 | Industry | Constituency building | Establish relationships with key opinion leaders and health organisations | Visit Nestlé’s Choose Wellness Roadshow in the school holidays [...] Partnering with some of Australia’s leading health and physical education bodies – including the **Dietitians Association of Australia and the Glycemic Index Foundation** – the Nestlé Choose Wellness Roadshow will visit six centres |  | http://www.nestlechoosewellness.com.au/your-wellbeing/choose-wellness-choose-to-visit-the-nestle-choose-wellness-roadshow-in-the-school-holidays/ |
| A189 | Industry | Constituency building | Establish relationships with key opinion leaders and health organisations | Nestle Professional - Our Partners: Dietitians Association of Australia |  | https://www.nestleprofessional.com/australia/en/Insights/Our-NHW-Vision/Pages/OurPartners.aspx |
| A190 | Industry | Constituency building | Establish relationships with key opinion leaders and health organisations | Nestlé recognises the importance of the Heart Foundation Tick Program as a guide to helping people make healthier food choices. 100% of UNCLE TOBYS cereals meet the Heart Foundation Tick nutrient criteria. |  | http://www.nestle.com.au/nhw/our-nutrition-partners |
| A191 | Industry | Constituency building | Establish relationships with key opinion leaders and health organisations | Nestle Professional - Our Partners: Glycemic Index Foundation |  | https://www.nestleprofessional.com/australia/en/Insights/Our-NHW-Vision/Pages/OurPartners.aspx |
| A192 | Industry | Constituency building | Establish relationships with key opinion leaders and health organisations | Nestle Professional - Our Partners: Heart Foundation |  | https://www.nestleprofessional.com/australia/en/Insights/Our-NHW-Vision/Pages/OurPartners.aspx |
| A193 | News | Constituency building | Establish relationships with key opinion leaders and health organisations | Nestlé Chairman Peter Brabeck, the company’s CEO, Paul Bulcke and **UNCTAD’s Secretary-General Mukhisa Kituyi** will be joined by Arancha Gonzalez, the Executive Director of the International Trade Centre; Mark R. Kramer of the Harvard Kennedy School of Government; **Ruth Oniango, Professor of Nutrition at Kenya’s Great Lakes University as well as the Secretary General of the International Federation of the Red Cross and Red Crescent Societies, As Sy**. |  | http://www.nestle.com.au/media/newsandfeatures/new-role-business-nestle-creating-shared-value-forum |
| A194 | Twitter | Constituency building | Establish relationships with key opinion leaders and health organisations | Nestle Australia ‏@nestleaunews RT @Nestle: #ICYMI: How can we solve the problem of Hidden Hunger? http://bddy.me/1ACdh2H | And then link to Nestle's website with publications funded by Nestle and then solutions with their products:  "Most recently, we launched a joint study and communications campaign in the Philippines, in collaboration with the Food National Research Institute, to encourage parents to give schoolchildren iron-fortified milks every day, and to measure the impact over time. (...) As an industry, we’re showing we’re credible partners, going beyond using our scientific knowhow to put micronutrients safely in a product and ensure they’re preserved until the end of its shelf life. We’re investing more in research that demonstrates these products are making a difference. And we’re doing more to use our marketing knowhow to help educate people to choose more nutritious products, at a price they can afford." | http://www.nestle.com/media/newsandfeatures/insight-joerg-spieldenner-micronutrient-deficiency?app_data={%22pi%22:%2255653_1415977072_2088302348%22,%22pt%22:%22twitter%22} |
| A195 | PH | Constituency building | Establish relationships with key opinion leaders and health organisations | Dietitians Association Australia Major partners: Nestle |  | http://daa.asn.au/advertising-corporate-partners/major-partners/ |
| A196 | Industry | Constituency building | Establish relationships with key opinion leaders and health organisations | Glycemic Index Program Nestlé is a supporter of the GI Symbol Program, a public health initiative which provides **accurate and balanced information** on the benefits of low GI and **sound nutrition**. |  | http://www.nestle.com.au/nhw/our-nutrition-partners |
| A197 | Government | Constituency building | Establish relationships with policy makers | FSANZ Update of Register of Interests - July 2014: - Dr David Roberts: Member – Science and Nutrition Advisory Group for Nestlé |  | http://www.foodstandards.gov.au/about/board/Documents/Update%20of%20Register%20of%20Interests%20-%20July%202014.pdf |
| A198 | Government | Constituency building | Establish relationships with policy makers | FSANZ Update of Register of Interests - July 2014: - Professor Lynne Daniels: Research Funding past – 1999-2001 Nestle |  | http://www.foodstandards.gov.au/about/board/Documents/Update%20of%20Register%20of%20Interests%20-%20July%202014.pdf |
| A199 | Government | Constituency building | Establish relationships with policy makers | Dietary Guidelines Working Committee - Conflict of interest information: *Professor Peter Davies Has received financial support to attend meetings for Nestle, Bayer, Nutrica, Danone, Wyeth, Pfizer, Ipsen, Novo Nordisk, Merck Serono. Peter's research group will receive a non-specific grant of $25,000 from Nestle Nutrition Institute (NNI) in return for his time. NNI will also be paying for travel and any accommodation costs. Agreed to take part in 5 Nutrition Update Days jointly run by NNI and Nutrition Society of Australia in 2011.  Gave two talks at each venue 1) Infant Feeding and later outcomes 2) Nutrition in pregnancy, getting the best outcomes.  Gave talks to either paediatricians and/or other health professionals at NNI sponsored event on 5 occasions in 2011 entitled 'Infant feeding and later outcomes'. |  | http://www.nhmrc.gov.au/your-health/nutrition/dietary-guidelines-working-committee/declarations-conflict-interest-dietary |
| A200 | Government | Information and messaging | Promote deregulation | FSANZ Proposal 293 Nutrition Health and Related Claims Consultation Submission by Nestle: "Not permitting portion controlled snacks to communicate about these benefits **restricts the ability for consumers to receive this type of information** and a greater understanding of energy balance and ‘weight maintenance’.” [page 5] " Seeking Pre-approval to have new health claims […] [is] potentially **deny consumers within the community beneficial products, nutrition information and education**" [page 6] |  | http://www.foodstandards.gov.au/code/proposals/Documents/P293_consultation_2012.zip |
| A201 | Government | Information and messaging | Promote deregulation | FSANZ Proposal 293 Nutrition Health and Related Claims Consultation Submission by Nestle: " a co-regulatory approach […] would [be] […] cost effective overall, and **not more trade restrictive than necessary**" [page 4] On health claims: " Seeking Pre-approval to have new health claims […] [is] a **costly, resource intensive and a potentially slow process**. This will **discourage R & D investment** [...]. It is unlikely that small to medium size businesses will be able to engage easily in seeking health claim pre-approval as there would be a **significant business burden imposed**." [page 6] On Nutrient Profiling Scoring Criteria: "NPSC will ultimately **undermine the competitiveness of the Australian & New Zealand food manufacturers, thereby affecting their longer term viability**." [page 8] "creating **unfair competitive issues** for Australian and New Zealand manufacturers" [page 9] |  | http://www.foodstandards.gov.au/code/proposals/Documents/P293_consultation_2012.zip |
| A202 | Industry | Information and messaging | Stress the economic importance of the industry | "Nestlé Oceania employs more than 6,000 people, operates 12 factories, 5 distribution centres and 20 offices across the Oceania region - taking in Australia, New Zealand and the Pacific Islands." |  | http://www.nestle.com.au/aboutus |
| A203 | Government | Information and messaging | Frame the debate on diet- and public health-related issues the debate on diet- and public health-related issues | FSANZ Proposal 293 Nutrition Health and Related Claims Consultation Submission by Nestle: "This type of approach is also consistent with the Policy Guidance provided by: [...] supporting government, community **and industry** initiatives that promote healthy food choices by the population" "optimises potential to educate consumers on a balanced diet" "**consumer education and information activities** can be undertaken where claims will no longer be represented on food products." [page 4] "Nestlé suggests that ‘the concept of prohibiting the use of claims on certain foods on the basis of their nutritional profile is **contrary to the basic principle in nutrition that there are no ‘good’ and ‘bad’ foods but rather ‘good’ and ‘bad’ diets**" [page 8] On fat free and % fat-free claims: "The **business has decided to focus on enjoyment of these ‘sometimes’ treat products in the context of an overall balanced diet**." [page 10] On Nutrient Profiling Scoring Criteria: "The food industry needs to be able to communicate or claim the benefits of food to provide consumers with information to make appropriate food choices for their particular lifestyle." [page 8] |  | http://www.foodstandards.gov.au/code/proposals/Documents/P293_consultation_2012.zip |
| A204 | Government | Information and messaging | Frame the debate on diet- and public health-related issues the debate on diet- and public health-related issues | FSANZ Proposal 293 Nutrition Health and Related Claims Consultation Submission by Nestle:  On Nutrient Profiling Scoring Criteria (NPSC): "The selection of a **balanced diet** should be a **matter of choice based on the judicious and preferential selection of foods by consumers**.” [page 8] |  | http://www.foodstandards.gov.au/code/proposals/Documents/P293_consultation_2012.zip |
| A205 | Industry | Information and messaging | Frame the debate on diet- and public health-related issues the debate on diet- and public health-related issues | "Nestle Healthy Kids Program - Healthy Kids is a global program which focuses on improving basic knowledge of nutrition and physical activity levels in children around the world. The global program is aimed at improving the nutritional status of school children aged 6 – 16 years old and includes both nutrition education and physical activity programs at the national level. In 2001 Nestlé Australia developed, in partnership with the Australian Institute of Sport (AIS), the Healthy Active Kids resource. It has the common goal of helping Australian families get active and healthy." o "We realise how a busy life can make it challenging to **eat well and be active**. So we have put together, with the help of nutritionists, **healthy eating tips** for every part of your child’s day. We’ve got great articles on **breakfast, tips for packing a balanced lunchbox and also ideas on how to get your kids cooking**" o "A good balance", "Enjoy variety", "Keep hydrated" o "Whether it’s playing with friends or family, or taking part in sports and other physical activities, being active is good for everyone!" o "Playing for Life Resource Kit & Companion Book Kit" o Lot of information on physical activity:  https://www.healthyactivekids.com.au/families/some-to-read/ Health and Activity Articles:  10 Ways to get Kids Active  Eating your Fruit and Vegies  Getting Kids into Sport  Getting Kids to Eat their Vegies  Staying Active with Your Kids | A dedicated website for kids with a lot of information!  Only illustrative examples presented here Recipes promote Nestlé's products Booklet: 7 out of 20 pages on physical activity or sport (including cover and back) | https://www.healthyactivekids.com.au/about-the-resource/ais-nestle/ http://www.nestle.com.au/nhw/healthy-active-kids |
| A206 | Industry | Information and messaging | Frame the debate on diet- and public health-related issues the debate on diet- and public health-related issues | "Nestle Good Life Program - The Nestlé Good Life Program is a group of community initiatives covering a wide diversity of activity and interest in the areas of sport, health, nutrition, education, food and promoting active lifestyles We are also focused on improving the environmental sustainability within the communities we operate in. This includes our major nutrition education partnership with the Australian Institute of Sport, our Milo in2CRICKET program with over 600,000 kids participating in cricket initiatives in 2012, the Nestle community Environment Program, The Nestlé Golden Chef’s Hat Award, Meals on Wheels, UNCLE TOBYS Swim, Survive, Stay Alive Program and various other initiatives." "Good Food, Good Life in the community Our community partnerships, related programs and sponsorship activities here in Oceania all come together under the Nestlé Good Life Program. To have the greatest possible impact, we focus on areas where we believe we can add the most value: food, nutrition, and health and wellness. These are areas where we can best contribute our expertise, scientific insight and decades of experience. We also support a range of environmental initiatives in our communities.​" |  | http://www.nestle.com.au/csv/good-life-program http://www.nestle.com.au/creating-shared-value/community-programs |
| A207 | Industry | Information and messaging | Frame the debate on diet- and public health-related issues the debate on diet- and public health-related issues | "We believe that we have a **shared responsibility** – together with governments, other companies, non-government agencies, the healthcare profession, and other community stakeholders – to effect positive nutritional and lifestyle change through the business we do and the consumers we serve throughout life." "We recognise three dimensions of Nutrition, Health and Wellness.  **Pleasure**: Winning consumer preference on taste and pleasure while enhancing the nutritional value of our products.  **Balance**: Encouraging responsible nutrition and moderation and variety in food habits. **There is no such thing as bad food.**  **Understanding**: Providing clear nutritional advice and information to **help consumers make informed choices** about their diet and lifestyle." "A **balanced eating plan, along with some moderate intensity physical activity**, is the best and safest approach to losing weight and keeping your shape." - and then tips for physical activity and weight loss | Too many webpages - Only illustrative examples presented here | http://www.nestle.com.au/creating-shared-value/nutrition http://www.nestle.com.au/nhw/our-promise/our-commitment-to-nutrition-health-and-wellness http://www.nestle.com.au/nhw/staying-in-shape |
| A208 | Industry | Information and messaging | Frame the debate on diet- and public health-related issues the debate on diet- and public health-related issues | Nestle **Choose Wellness** website  Categories:  TAKE THE QUIZ  EATING WELL   STAYING HEALTHY - general  YOUR WELLBEING - general  **GETTING ACTIVE**    **Recipes.com.au -** All recipes contain at least one product from Nestle, some recipes qualified as "healthy" | Too much information - Only illustrative examples presented here - not only specific to nutrition (physical activity, mental health, smoking, alcohol, etc.) | http://www.nestlechoosewellness.com.au/get-active/ |
| A209 | News | Information and messaging | Frame the debate on diet- and public health-related issues the debate on diet- and public health-related issues | A new role for business: Nestlé’s Creating Shared Value Forum Participants will discuss how civil society and the private sector can work in partnership to strengthen and speed up sustainable development, focusing on the key areas of nutrition, water and rural development. |  | http://www.nestle.com.au/media/newsandfeatures/new-role-business-nestle-creating-shared-value-forum |
| A210 | Twitter | Information and messaging | Frame the debate on diet- and public health-related issues the debate on diet- and public health-related issues | Nestle Australia ‏@nestleaunews  Check out what we are doing to help kids lead healthy and active lives. http://bit.ly/1r5qCtU  Link to website: **Five schools across Australia will improve their nutrition and physical education facilities after each was awarded a $5000 grant from Nestlé Healthy Active Kids.** More than 1,000 grant applications were received with requests to fund a wide variety of projects such as upgrading playgrounds, new bubblers, shade cloths, breakfast programs and sports equipment. The winning schools in New South Wales, Victoria and Western Australia have welcomed the boost to their school projects around health and activity. [...] Since January more than 25,000 teachers have accessed the online lesson plans – a 194 per cent increase from early 2013. The new website has also had around 220,000 views over the same period, up 340 per cent. [...] The online resource builds on a 15 year partnership between the AIS and Nestlé and includes more than 80 lesson plans, videos, worksheets, handouts and online games and quizzes. **Head of Nestlé Nutrition, Health and Wellness, Katrina Koutoulas, said teaching children about nutrition and active living was an investment in their future and that of Australia.** **"The Healthy Active Kids Global Programme and all the initiatives that are part of this, are designed to help schools and their communities to provide the best healthy, active education that they can, as well as giving parents the latest nutrition information available,” Ms Koutoulas said.** |  | https://twitter.com/nestleaunews/status/507412783361585152 http://www.nestle.com.au/media/newsandfeatures/helping-kids-lead-healthy-active-lives |
| A211 | Twitter | Information and messaging | Frame the debate on diet- and public health-related issues the debate on diet- and public health-related issues | Choose Wellness events: -Nestle Australia ‏@nestleaunews Don't let your kids climb the walls these school holidays, come and climb ours at Nestle Choose Wellness events. http://bit.ly/1x3do8v  4:36 PM - 22 Sep 2014  -Nestle Australia ‏@nestleaunews Olympic swim stars the Campbell sisters at Nestle Choose Wellness event with @TheTodayShow pic.twitter.com/LZxtGjAnjf 2:35 PM - 24 Sep 2014  -Nestle Australia ‏@nestleaunews Cate and bronte Campbell give their fitness tips at Nestle Choose wellness event with @TheTodayShow pic.twitter.com/ovioKpNqML 2:44 PM - 24 Sep 2014  -Nestle Australia ‏@nestleaunews Visit the Nestle Choose wellness event at Westfield shopping centres. http://Www.nestlechoosewellness.com.au  2:56 PM - 24 Sep 2014  -Nina CurtisVerified account ‏@NinaEvaCurtis Had an awesome time putting some kids through the paces with the @nestleaunews #choosewellness program on the @TheTodayShow this morning. Sydney, New South Wales 3:41 PM - 24 Sep 2014  Margaret Stuart ‏@Marg_A_Stuart Sep 24  @NinaEvaCurtis @nestleaunews @TheTodayShow It was great to have you here - thank you!  Nina Curtis ‏@NinaEvaCurtis Sep 24  @Marg_A_Stuart @nestleaunews Thanks Margaret it was lovely to meet you!  Margaret Stuart ‏@Marg_A_Stuart Sep 24  @NinaEvaCurtis I've ordered roof bars. There's a kayak in my near future!  -Nestle Australia ‏@nestleaunews Thank you @NinaEvaCurtis for being part of the nestle choose wellness event! @TheTodayShow 3:43 PM - 24 Sep 2014  -Nestle Australia ‏@nestleaunews Olympic swimming stars Cate and Bronte Campbell at Westfield Carindale today from 10am @catecamp @Bronte_Campbell #nestlechoosewellness 3:48 PM - 25 Sep 2014 | During school holidays Promote physical activity (use the image of athletes) | https://twitter.com/nestleaunews/status/514196504534212608 https://twitter.com/nestleaunews/status/514890947398602752 https://twitter.com/nestleaunews/status/514893124556959746 https://twitter.com/nestleaunews/status/514896175514664960 https://twitter.com/NinaEvaCurtis/status/514907512227627008 https://twitter.com/nestleaunews/status/514907897608671232 https://twitter.com/nestleaunews/status/515271634706374656 |
| A212 | Twitter | Information and messaging | Frame the debate on diet- and public health-related issues the debate on diet- and public health-related issues | Retweeted by Nestle Australia Nestlé CSV ‏@nestlecsv Peter Brabeck: Today is not only about large companies, but smaller companies, NGOs, investment community & other stakeholders. #CSVForum |  | https://twitter.com/nestlecsv/status/520111492990238721 |
| A213 | Twitter | Information and messaging | Frame the debate on diet- and public health-related issues the debate on diet- and public health-related issues | Nestle Australia ‏@nestleaunews #DidYouKnow your kids are more likely to eat fruit & vegetables they've helped prepare? http://bddy.me/1zK4rze   And then link to their website and a paper funded by Nestle:  "Nestlé aims to **help parents and children make healthier choices**, running cookery schools and educational programmes around the world including in countries like Germany, Thailand, Venezuela and India." |  | https://twitter.com/nestleaunews/status/542446120484339712 http://www.nestle.com/media/newsandfeatures/children-vegetables |
| A214 | Twitter | Information and messaging | Frame the debate on diet- and public health-related issues the debate on diet- and public health-related issues | Nestle Australia retweeted cereal4brekkie @cereal4brekkie · Feb 16 What’s your pledge this Healthy Weight Week #AHWW? Check out these tips on how to make breakfast work for you http://bit.ly/1x7p9XI | Tweets are promoted (sponsored) and appeared on people's timelines | https://twitter.com/cereal4brekkie/status/567104051355996160 |
| A215 | FOI request | Information and messaging | Frame the debate on diet- and public health-related issues the debate on diet- and public health-related issues | February 29th, 2012 Nestle Australia Ltd Response to Australian Dietary Guidelines Incorporating the Australian Guide to Healthy Eating. Draft for Public Consultation National Health and Medical Research Council December 2011  Recommendation 3 : Dietary Guideline 2 section 3.3.4 “• Replace the dietary recommendation on added sugars with a more positive, behaviour change that focuses on the selection of a variety of nutrient dense foods.” [...] Recommendation 5; Dietary Guideline 3 section 4.2.2 “• An acknowledgement of effective types of weight management (e.g. behavioural treatments, surgery, pharmacotherapy and self-guided or commercial weight loss programs)”  “[...] we recommend the adoption of a more consumer-­‐friendly message to help guide consumers" | Nestle asked for this submission to be confidential |  |
| A216 | Lobby | Information and messaging | Lobby Establish relationships with policy makers | Lobby Register Parker & Partners Pty Ltd and Etched Communications Pty Ltd Client: Nestle and Nestle Australia |  | http://lobbyists.pmc.gov.au/export/export_client.cfm |
| A217 | Conference | Information and messaging | Shape the evidence base on diet- and public health-related issues evidence | Dietitians Association of Australia 31st National Conference - Sponsored Breakfast Seminars: Nestlé Corporate: Unlocking the facts on kid’s snack habits |  | http://arinex.com.au/dietitians2014/wp-content/uploads/2014/05/DAA-Conference-2014-Program-Only-PR-Version.pdf http://arinex.com.au/dietitians2014/sponsored-breakfast-seminars/ |
| A218 | Conference | Information and messaging | Shape the evidence base on diet- and public health-related issues evidence | Dietitians Association of Australia 31st National Conference - Sponsored Breakfast Seminars: Nestlé Health Science: What’s on the menu at the global village? Mid project update on the International Dysphagia Diet Standardisation Initiative |  | http://arinex.com.au/dietitians2014/wp-content/uploads/2014/05/DAA-Conference-2014-Program-Only-PR-Version.pdf http://arinex.com.au/dietitians2014/sponsored-breakfast-seminars/ |
| A219 | Conference | Information and messaging | Shape the evidence base on diet- and public health-related issues evidence | ANZOS 2014 Supporter - Exhibitors Nestle Healthscience |  | http://www.anzos2014.org.au/sponsorship/ |
| A220 | Conference | Information and messaging | Shape the evidence base on diet- and public health-related issues evidence | 6th Exercise & Sports Science Australia Conference and Sports Dietitians Australia Our Exhibitors - Musashi |  | https://www.essa.org.au/2014conference/sponsorship-exhibition/ |
| A221 | Conference | Information and messaging | Shape the evidence base on diet- and public health-related issues evidence | Dietitians Association of Australia 31st National Conference - Exhibitors: Nestlé Corporate |  | http://arinex.com.au/dietitians2014/sponsorship-exhibition/ |
| A222 | Conference | Information and messaging | Shape the evidence base on diet- and public health-related issues evidence | Dietitians Association of Australia 31st National Conference - Exhibitors: Nestlé Health Science |  | http://arinex.com.au/dietitians2014/sponsorship-exhibition/ |
| A223 | FOI | Information and messaging | Shape the evidence base on diet- and public health-related issues evidence | Nestle Nutrition Institute: Symposim 2014 - presentation on "Proteins" - a person from Curtin University presented |  |  |
| A224 | Industry | Information and messaging | Shape the evidence base on diet- and public health-related issues evidence | "Nestlé Healthy Active Kids is a global program which aims to improve the nutrition and health knowledge and promote physical activity among school-aged children by developing local programs and operating through multi-partnership approaches. In 2013 the program was operating in 68 countries, reaching 6.9 million children. In Australia we re-launched the website enhancing resources for teachers by adding new lesson plans relating to healthy eating and exercise. As part of the program we distributed 80,000 Kids Nutrition Plates, 50,000 Healthy Active Kids booklets and as a result was able to reach 5,000 teachers and 250,000 school children." o "Playing for Life Resource Kit & Companion Book Kit" o A curriculum on food and nutrition for each State in Australia - with a lot of units and material for teachers! 6 out of 8 units on other things than nutrition - and in the nutrition units, a lot of information on hydration rather than foods o Booklet for teachers: https://www.healthyactivekids.com.au/wp-content/uploads/2013/09/AIS040_HEALTHY_KIDS_2013_BOOKLET_WEB.pdf | A dedicated website with a lot of information - Only illustrative examples presented here Recipes promote Nestlé's products Booklet: 7 out of 20 pages on physical activity or sport (including cover and back) | https://www.healthyactivekids.com.au/about-the-resource/ais-nestle/ http://www.nestle.com.au/nhw/healthy-active-kids |
| A225 | Industry | Information and messaging | Shape the evidence base on diet- and public health-related issues evidence | "Survival for the Active Family' is the fourth official cookbook from the Australian Institute of Sport and Nestlé following on from the popular Survival cookbook series 'Survival for the Fittest', 'Survival from the Fittest' and 'Survival around the World'." "available from all major bookstores around Australia" | Promote Nestle's products | http://www.nestle.com.au/nhw/resources/survival-cookbooks |
| A226 | Industry | Information and messaging | Shape the evidence base on diet- and public health-related issues evidence | [Duplicate] Nestlé has partnered with the Nutrition Society of Australia to jointly host over 20 Nutrition Update Days for healthcare professionals. Nestlé also supports an annual Emerging Researcher Award for Nutrition Society members. |  | http://www.nestle.com.au/nhw/our-nutrition-partners |
| A227 | Industry | Information and messaging | Shape the evidence base on diet- and public health-related issues evidence | Australian Institute of Sport Nestlé has partnered with the AIS since 1999 and has helped bring over 32 years of sports nutrition and fitness expertise into the lives of everyday Australians. This includes free nutrition education resources for school teachers |  | http://www.nestle.com.au/nhw/our-nutrition-partners |
| A228 | Industry | Information and messaging | Shape the evidence base on diet- and public health-related issues evidence | Visit Nestlé’s Choose Wellness Roadshow in the school holidays  [...], **find out if you’re at risk of diabetes and speak to an accredited dietitian** at the Nestlé Choose Wellness event being held at a select number of Westfield shopping centres during the school holidays. [...] Highlights of the Nestlé Choose Wellness events include:   **Advice from local Accredited Practising Dietitians (APDs)  Free Diabetes testing conducted by the Australian Diabetes Council** (from 10am to 2pm each Friday at each location) [...] At all locations, you’ll also be able to **learn how Nestlé products are the ideal partners to help you invite more fresh food into your diet, such as:  Milk with MILO for nourishing energy  Vegies with MAGGI for fresh meal ideas; and  Wholegrains with UNCLE TOBYS to start your day with a nutritious breakfast** [...]Nestlé’s Head of Nutrition, Health and Wellness, Katrina Koutoulas, says it is all **part of Nestlé’s commitment to helping improve the lives of Australians**. “**As one of the leading Nutrition, Health and Wellness companies**, we want to help Australians to care for themselves and their families. | Promotion of their products | http://www.nestlechoosewellness.com.au/your-wellbeing/choose-wellness-choose-to-visit-the-nestle-choose-wellness-roadshow-in-the-school-holidays/ |
| A229 | Industry | Information and messaging | Shape the evidence base on diet- and public health-related issues evidence | Visit Nestlé’s Choose Wellness Roadshow in the school holidays **Learn how to read food labels** [...] Highlights of the Nestlé Choose Wellness events include:  Free adults and children **portion plates** to give away  A child friendly 2.2 metre high **climbing wall**  **Healthy cooking** demonstrations  Help on how to **understand food labels** [...]And visitors to the Nestlé Choose Wellness event in Brisbane (Westfield Carindale) will be in for an extra treat when Commonwealth **Games Gold Medalists** Cate and Bronte Campbell visit on Friday, 26 September from 10am to 12pm. [...] The Nestlé Choose Wellness Roadshow is designed to give people tips and answer questions that will **help them to live healthy active lives**,” Ms Koutoulas said. So **choose to do something for yourself and your family** and join us at a Nestlé Choose Wellness event near you. It’s **a choice** you’ll be glad you made! |  | http://www.nestlechoosewellness.com.au/your-wellbeing/choose-wellness-choose-to-visit-the-nestle-choose-wellness-roadshow-in-the-school-holidays/ |
| A230 | Industry | Constituency building | Seek involvement in the community | "Through consultations with Indigenous leaders and communities, Nestlé realised that Aboriginal girls and women play a pivotal role in the community, yet their needs are often overlooked. So with this focus, Nestlé developed a way to engage in early intervention programs to support and educate young people, in partnership with local Indigenous organisations and communities. As part of this, Nestlé partnered with Role Models and Leaders Australia (RMLA), a not-for-profit organisation which aims to develop and empower Indigenous girls through leadership, sport and education. The RMLA Girls program, based in high schools, now works with more than 1000 girls and has significantly increased the number of girls completing secondary school. **An important part of our involvement with RMLA is the Nestlé Mother Daughter Nutrition Program, which teaches Aboriginal girls and their mothers the value of good nutrition and healthy eating.** This program, developed with the expertise and hands-on involvement of the Nestlé Head Nutritionist and Head Chef, teaches girls and their mothers how to plan and shop for nutritious, balanced meals, and how to prepare simple, healthy meals. The program also incorporates foods that are part of a traditional diet." |  | http://www.nestle.com.au/creating-shared-value/community-programs/indigenous-community-contributions |
| A231 | Industry | Information and messaging | Shape the evidence base on diet- and public health-related issues evidence | "Oats are truly one of nature’s superfoods. (…) When grains contain awesome combinations of protein, fibre, vitamins and minerals, we call them Superfoods or supergrains.  And there are other benefits to oats, such as: • **Cholesterol lowering** – Eating oats daily, as part of a balanced, healthy diet, will help your heart health by lowering cholesterol re-absorption. Beta glucan, found in the bran and endosperm layer of the oat grain, helps to lower cholesterol re-absorption levels by inhibiting the re-absorption of cholesterol back into the bloodstream. Research shows that 3g of beta glucan every day will help to lower cholesterol re-absorption. • **Providing fibre for digestive health** – Oats are also high in insoluble fibre. Insoluble fibre passes through the digestive system without being absorbed, which bulks up stools and helps keep bowel movements regular. • **A natural source of energy** – Oats are a natural wholegrain that is high in fibre and also provides carbohydrates and protein for energy. As a minimally processed, high fibre, low sodium grain, oats with milk are a great way to fuel your mornings." | No references | http://www.nestlechoosewellness.com.au/featured/oats-the-super-food/ |
| A232 | Industry | Information and messaging | Shape the evidence base on diet- and public health-related issues evidence | "Nestlé is the l**argest employer of Dietitians and Nutritionists, with approximately 30 working across the business in Oceania alone**. **Nestlé invests over AUD$1.6 billion in Research and Development every year**, more than any other food company. Nestlé works with a network of over 5,000 scientists and technologists to advance the frontiers of nutritional science, by developing safer, healthier and better tasting products. Nestlé provides nutrition based training for 5,500 employees across Oceania. The training focuses on improving nutrition knowledge and aims to empower employees to make informed food and beverage choices for themselves and their families. |  | http://www.nestle.com.au/nhw/tastier-and-healthier-choices |
| A233 | Industry | Information and messaging | Shape the evidence base on diet- and public health-related issues evidence | Nutritional information and advantages for health of consuming Milo - but no references |  | http://www.nestle.com.au/asset-library/documents/milo%20infographic%20lifting%20lid%20final_v3.pdf |
| A234 | Industry | Information and messaging | Shape the evidence base on diet- and public health-related issues evidence | Oats for health: A research summary for health professionals | Authors from the food industry (third parties: Grains & Legumes Nutrition Council, Australia)  Too many references to verify if they have been funded by the industry - potential case study on Nestle ‘materials  Random examples:   Reference **unpublished**: Grains & Legumes Nutrition Council 2013  References **from the industry**:  o AEGIC (Australian Export Grains Innovation Centre) (2012)  o Primary Industries Standing Committee. Grains Industry National Research, Development and Extension Strategy. 2011 o Williams, P (2007). "The author planned and commissioned these analyses when previously employed as **Director of Scientific and Consumer Affairs at Kellogg (Aust) Pty Ltd**."  References funded by the industry:  o Saltzman, E, Das, SK, et al. (2001)."**Supported by in part by an unrestricted gift from the Quaker Oats Company** (Barrington, IL)"http://jn.nutrition.org/content/131/5/1465.long o Keshavarzian, S, Choudhary, E et al. (2001). "This study was **supported in part by a grant from The Quaker Oats Company,** Barrington, IL, a research grant from the American College of Gastroenterology, and a grant from Rush Medical School."http://jpet.aspetjournals.org/content/299/2/442.long | http://www.nestle.com.au/asset-library/documents/cpw017%20oats%20for%20health%20booklet%20spreads.pdf |
| A235 | Industry | Information and messaging | Shape the evidence base on diet- and public health-related issues evidence | Unlocking the facts on kids' snack habits: the first in-depth exploration of national data on snacking behaviours in Australian children "the research showed that higher total daily number of snacking occasions (so) was not associated with higher BMI z-score. children who were of normal weight had the same number of daily so as children who were overweight or obese." | Too many references to verify if they have been funded by the industry - potential case study on Nestle's materials  Random examples:  References **from the industry**: o Hartmann C, Siegrist M, van der Horst K. 2012 - On of the author's "Present address: Food Consumer Interaction, **Nestlé Research Center,** PO Box 44, 100 Lausanne 26, Switzerland."http://journals.cambridge.org/action/displayAbstract?fromPage=online&aid=8951213&fileId=S1368980012003771 o Berteus Forslund H et al (2005) - "This study was supported by the Swedish Research Council (grant 05239) and **F Hoffmann-La Roche.**" http://www.nature.com/ijo/journal/v29/n6/full/0802950a.html | http://www.nestle.com.au/asset-library/documents/unc058%20hcp%20snacking%2012pp%20%283%29.pdf |
| A236 | Industry | Information and messaging | Shape the evidence base on diet- and public health-related issues evidence | Nutritpro: Nestle Professional nutrition magazine:  NutriPro 7 June 2014: "Carbohydrates alone won’t cause weight gain. Carbohydrates have a similar energy (kilojoule) value to protein and only half the energy value of fat. It’s the total amount of energy (kilojoules) you eat that really counts when you are watching your weight." | References **from the food industry**: Grains & Legumes Nutrition Council, Nestle, CPW  Random example of reference **funded by the industry:**  Flight, I and Clifton P. Eur J Clin Nutr 2006 - "This study was **supported in part by BRI Australia Ltd**." (Bread Research Institute) http://www.nature.com/ejcn/journal/v60/n10/full/1602435a.html#ack  Only a few peer reviewed references | http://www.nestle.com.au/asset-library/documents/nfs197%20nutripro%20carbohydrates%202014_final110614.pdf |
| A237 | Industry | Information and messaging | Shape the evidence base on diet- and public health-related issues evidence | "Boosting children’s calcium intake How MILO and MILK can help - Information for Healthcare Professionals" | Too many references to verify if they have been funded by the industry - potential case study on Nestles 'materials  References **from the dairy industry and funded by the dairy industry** (Johnson RK et al. 2002 " This research **was funded by the National Dairy Council**" http://www.eatsmart.org/client_images/gd20052171415271.pdf) | http://www.nestle.com.au/asset-library/documents/dietitian-brochure-milo%20and%20milk.pdf |
| A238 | Industry | Information and messaging | Shape the evidence base on diet- and public health-related issues evidence | Nutrition fact sheets Nutrition for everyone Tips for kids | Too much information - cannot verify every statement No references for scientific statements | http://www.nestle.com.au/nhw/nutritionfactsheets http://www.nestle.com.au/nhw/nutrition-for-everyone http://www.nestle.com.au/nhw/tips-for-kids |
| A239 | Industry | Information and messaging | Shape the evidence base on diet- and public health-related issues evidence | Nestle Health Science - genetic approach to nutrition "We aim to bring a new dimension to the nutrition, health and wellness strategy of Nestlé Group by pioneering the development and application of evolving science to create a new role for nutrition in disease prevention and management. " "Nestlé Health Science aims to pioneer **a new industry between the traditional nutrition and pharmaceutical industries** through the development of **science-based** personalised nutritional solutions and **shaping a new approach to disease prevention and management**. Building on its founding core HealthCare Nutrition business, a leading player in the medical nutrition industry, the Nestlé Health Science has ambitions to address chronic conditions in the area of Gastrointestinal Health, Metabolic Health and Brain Health." "We aim to **elevate the role of nutrition across six distinct areas.** Our focus is in specialties in which we are equipped to lead; by bringing game changing nutritional solutions to market. **Ageing Medical Care, Critical Care and Surgery and Paediatric Medical Care** are part of our core HealthCare Nutrition business, upon which Nestlé Health Science was founded. Through innovation and renovation we continue to drive growth in our core established platforms that provide a complete range of enteral and oral nutritional products. To deepen and establish nutrition’s role in managing the increasingly prevalent chronic and lifestyle related illnesses, we have recently established platforms in **Brain Health, Metabolic Health and Gastrointestinal Health**. The acquisition of companies such as Prometheus Laboratories and CM&D Pharma, provides brands, and more importantly expertise and enhanced innovation potential, that support our pioneering efforts in these platforms." |  | http://www.nestlehealthscience.com.au/ |
| A240 | Industry | Information and messaging | Shape the evidence base on diet- and public health-related issues evidence | Nestle Professional - nutrition fact sheets |  | https://www.nestleprofessional.com/australia/en/Insights/Pages/NutritionFactSheets.aspx |
| A241 | Industry | Information and messaging | Shape the evidence base on diet- and public health-related issues evidence | Nestle Choose Wellness website  References include: **Australian Dairy Corporation., Grains & Legumes Nutrition Council, Meat and Livestock Australia** | Too much information - not only specific to nutrition (physical activity, mental health, smoking, alcohol, etc.) | http://www.nestlechoosewellness.com.au/eating-well/nutrition-links/ |
| A242 | Industry | Information and messaging | Shape the evidence base on diet- and public health-related issues evidence | "**Nestlé is a proud member of Grains & Legumes Nutrition CouncilTM**. As a member of the organisation Nestlé has access to independent data and scientifically valid research findings which can be used to assist in the development and improvement of our products. Grains & Legumes Nutrition CouncilTM is Australia’s leading independent voice for grain foods and legumes in health and nutrition. Grains & Legumes Nutrition CouncilTM reviews science-based evidence on the health benefits of grains, grain food products and legumes and communicates these benefits to food processors, food manufacturers, health professionals, educators, consumers and the media." | Not independent if Nestle is a member | http://www.nestlechoosewellness.com.au/eating-well/go-grains-health-nutrition/ |
| A243 | News | Information and messaging | Shape the evidence base on diet- and public health-related issues evidence | For the third time this year a group of Nestlé employees went to Tamworth this week to run school holiday programs focusing on nutrition and fitness for more than 60 Indigenous children. |  | http://www.nestle.com.au/media/newsandfeatures/fun-farm-indigenous-kids |
| A244 | News | Information and messaging | Shape the evidence base on diet- and public health-related issues evidence | Insight: why process food? INSIGHT: Johannes Baensch, Nestlé Global Head of Research and Development  "when the same process is performed industrially, there’s little recognition of the expertise involved. In fact, most industrial food processing techniques are the outcome of years of research and development. Many are modelled on artisanal and traditional methods, and are often a **delicate balance of science and art**." 'We know that the best way to understand traditional methods of processing ingredients is to work with the local cultures they originate from. That’s why **we partner with research institutes, universities and government agencies worldwide, to learn from local knowledge while sharing our own experience**." "In many parts of the world, urban centres are expanding rapidly and increasing the distance from farm to fork within local regions. This is why some food processors, including **Nestlé, do not only focus on preserving the nutritional value of raw materials. They also find ways to enhance it.** Fortifying foods with micronutrients that are lacking in specific populations’ diets is one technique. And as technology, and our knowledge, continue to advance, processing should enable us to do more to **target the right nutrition, to the right people, at the right time**. " "In the west, we’ve come to expect that the food we buy is safe to eat, but it wasn’t always the case." "It is not enough to grow and harvest raw materials. **You need the expert know-how to turn them into safe, tasty, nutritious and convenient ingredients**. **Processed products may make our lives easier, but the skills and talent required to produce them are harder to come by than you might think**." |  | http://www.nestle.com.au/media/newsandfeatures/insight-johannes-baensch-processed-food |
| A245 | News | Information and messaging | Shape the evidence base on diet- and public health-related issues evidence | **Nestlé and other Board members of the Consumer Goods Forum (CGF) have announced the results of a global survey showing that industry efforts are helping to empower consumers to adopt healthier lifestyles**. The survey is the only global measurement of its kind in the industry. It is aimed at measuring progress CGF members are making in implementing health and wellness resolutions the CGF adopted in 2011. The resolutions identify specific areas for retailers and manufacturers to work together to support healthier diets and lifestyles for consumers.  “We understand the complexity of the health and wellness challenges facing communities across the globe and how no single solution or organisation can stimulate the adoption of healthier lifestyles, but we hope this report will help galvanise the industry into taking collective action for a positive change,” CGF Co-Chairs Paul Bulcke, Nestlé CEO and Dick Boer, Royal Ahold CEO said in a joint CGF statement. |  | http://www.nestle.com.au/media/newsandfeatures/survey-shows-firms-like-nestl%C3%A9-helping-empower-consumers-on-health |
| A246 | PH | Information and messaging | Shape the evidence base on diet- and public health-related issues evidence | The Nutrition Society of Australia (Inc.): NSA gratefully acknowledges the generous financial support provided for this award by the Nestle Nutrition Institute. |  | http://www.nsa.asn.au/index.php/awards_prizes_scholarships/nsa-nestle_emerging_student_researcher_award/ |
| A247 | PH | Information and messaging | Shape the evidence base on diet- and public health-related issues evidence | DAA’s Emerging Researcher Award is proudly supported by the Nestlé Nutrition Institute. Ms Lambert will receive a cheque for $1,000 and a complimentary pass to the DAA National Conference |  | http://daa.asn.au/wp-content/uploads/2014/05/Emerging-researcher-award_FINAL.pdf |
| A248 | Twitter | Information and messaging | Shape the evidence base on diet- and public health-related issues evidence | Tweets for the Nestle Nutrition Institute Regional Symposium | It seems that the symposium was held in parallel (?) to a conference of the South Australian Health and Medical Research Institute "Protein in Pediatrics: Consensus and Controversies" http://www.adelaidenow.com.au/news/south-australia/expert-says-some-bottlefed-babies-are-on-a-fasttrack-to-obesity/story-fni6uo1m-1227093181578 | https://twitter.com/nestleaunews/status/522879762004324352 |
| A249 | Twitter | Information and messaging | Shape the evidence base on diet- and public health-related issues evidence | Nestle Australia retweeted ABCMF @cereal4brekkie · Oct 21 230 research papers vs 1 pilot study - is skipping breakfast really better for you than a morning bowl of cereal? http://on.fb.me/1s5GnAM   Nestle Australia retweeted ABCMF @cereal4brekkie · Oct 21 30 yrs of research says people who eat breakfast cereal are slimmer than those who skip brekkie @HFGAustralia @smh http://bit.ly/1tXwFae | Reference to a website created by the AFGC: http://www.cereal4brekkie.org.au/abcmf/  "The Australian Breakfast Cereal Manufacturers Forum (ABCMF) " Reference to a paper: "This review was commissioned and paid for by the Australian Breakfast Cereal Manufacturers Forum of the Australian Food and Grocery Council." http://advances.nutrition.org/content/5/5/636S.full | https://twitter.com/cereal4brekkie/status/524777994284318720 |
| A250 | Twitter | Information and messaging | Shape the evidence base on diet- and public health-related issues evidence | NestléVerified account ‏@Nestle #ICYMI: Our new research into an enzyme called AMPK could change lives: http://bddy.me/1xHG726   Then on the website: Scientists at the Nestlé Institute of Health Sciences examined how our metabolism is regulated by a ‘master molecule’ which controls the energy balance of the body. The findings, published in the Journal Chemistry & Biology, could lead to the development of products to help those suffering from metabolic problems like obesity and chronic metabolic diseases such as Type 2 diabetes. These problems can become more prevalent in older people who are less active. The next phase of the research is to identify natural substances that can influence this molecular mechanism.  [...] “This could lead to the development of new dietary approaches with targeted effects on the body that, like exercise, could help in addressing metabolic problems and maintaining a healthy energy balance,” | And a picture: "Could food have a similar effect to exercise?" | https://twitter.com/Nestle/status/536572401170255872 http://www.nestle.com/media/newsandfeatures/ampk-metabolic-master-switch?app_data={%22pi%22%3A%2255653_1416585003_578729464%22%2C%22pt%22%3A%22twitter%22} |
| A251 | Twitter | Information and messaging | Shape the evidence base on diet- and public health-related issues evidence | Nestle Australia ‏@nestleaunews Food lovers - looking for healthy recipes? Follow us on Pinterest: http://bit.ly/1qjgZxY | Then link to recipes with Nestle products - they define what is healthy | https://twitter.com/nestleaunews/status/536735729682878465 |
| A252 | Twitter | Information and messaging | Shape the evidence base on diet- and public health-related issues evidence | Nestle Australia ‏@nestleaunews Why there's more to your 'gut feeling' than you realise: http://youtu.be/bkeBjP_9ZR4 via @TEDTalks |  | https://twitter.com/nestleaunews/status/540260655836561409 |
| A253 | Twitter | Information and messaging | Shape the evidence base on diet- and public health-related issues evidence | Nestle Australia ‏@nestleaunews RT @Nestle: #DidYouKnow that dark chocolate reduces stress hormones, helping you to relax? http://bddy.me/1AjXdTj  And then on their website: "Eating a moderate amount of dark chocolate every day can help reduce the hormones in your body that make you feel stressed, **according to a Nestlé study**. [...] Nestlé scientist Dr Sunil Kochhar, who led the study, explained the findings as part of his presentation “Cocoa and chocolate: The science of delight”, at the national meeting of the American Chemical Society (ACS) in California."  Another tweet 14 Dec 2014:  NestléVerified account ‏@Nestle #ICYMI: Good news for chocoholics - studies show dark chocolate reduces stress hormones. http://bddy.me/1GCdDYE | Link to their website: http://www.nestle.com/media/newsandfeatures/dark_chocolate_low_stress  #ICYMI: "In Case You Missed It" | <http://www.nestle.com/media/newsandfeatures/dark_chocolate_low_stress> |
| A254 | Twitter | Information and messaging | Shape the evidence base on diet- and public health-related issues evidence | Nestle Australia ‏@nestleaunews #ICYMI: As you get older, your skin needs a little extra care. How can Nestlé help? http://bddy.me/1AwHbF2   And then link to Nestle's website: "“We are creating an eco-system in which solutions and information can be developed to facilitate better maintenance, prevention, diagnosis and treatment strategies that will help people today and in the future enjoy improved well-being through a life course of healthy skin” says Humberto C. Antunes, Chief Executive of Nestlé Skin Health. “This will enable healthy and active ageing, benefitting individuals and society as a whole.” The new facilities will be known as Nestlé Skin Health Investigation, Education and Longevity Development (SHIELD) centres. The first centre will be opened in New York in 2015 with others to follow around the globe." |  | https://twitter.com/nestleaunews/status/544318267691524097  http://www.nestle.com/media/newsandfeatures/nestle-skin-health-shield-centres?app_data={%22pi%22%3A%2255653_1418402031_1624366883%22%2C%22pt%22%3A%22twitter%22} |
| A255 | Twitter | Information and messaging | Shape the evidence base on diet- and public health-related issues evidence | Nestle Australia retweeted cereal4brekkie @cereal4brekkie · Dec 9 Do you eat the same #brekkie as some of the world's top health experts? http://greatist.com/eat/expert-healthy-breakfasts … | Link to a blog - with no references | https://twitter.com/nestleaunews |
| A256 | Twitter | Information and messaging | Shape the evidence base on diet- and public health-related issues evidence | Nestle Australia retweeted cereal4brekkie @cereal4brekkie · Dec 9 New review of over 300 papers found #wholegrains more protective against type2diabetes, CVD & cancer than fruit & veg http://bit.ly/1G8tKgm   And then link to a paper of the International Life Science Institute (a **front group**): http://onlinelibrary.wiley.com/doi/10.1111/nure.12153/pdf |  | https://twitter.com/nestleaunews |
| A257 | Twitter | Information and messaging | Shape the evidence base on diet- and public health-related issues evidence | Nestle Australia ‏@nestleaunews #DidYouKnow your kids are more likely to eat fruit & vegetables they've helped prepare? http://bddy.me/1zK4rze   And then link to their website and a paper funded by Nestle:  "Children who help to prepare their own meals eat significantly more vegetables than those who are not involved in cooking, a **Nestlé study** published in the journal Appetite suggests. (...) **Another recent study carried out by Zurich’s ETH University and Nestlé** and published in the journal Public Health Nutrition showed that serving school-age children a greater variety of vegetables increased the quantity they chose to consume. The most recent study was carried out at **the Nestlé Research Center** (NRC), one of the company’s 34 Research & Development and Product Technology Centres around the world. The NRC’s 250 scientists publish some 200 peer-reviewed scientific publications each year across areas including nutrition and health, public nutrition and food consumer interaction. (...) In Germany, the **Maggi Kochstudio (Cooking Centre) offers cooking classes to adults and children, teaching them how to best combine Maggi with fresh ingredients to prepare tasty and balanced meals**. In 2013 alone, more than 9,400 participants attended these sessions. Nestlé has also recently published a guide for children and their parents with helpful tips on how to grow their own organic vegetables as part of its global Healthy Kids Programme."  And also a tweet, just a day before this one:  Nestle Australia @nestleaunews · Dec 8 Get your kids cooking in the kitchen with our easy Xmas recipes. http://bit.ly/1udaDMt  With link to the Nestle's website and recipes based on Nestle's products as ingredients: http://www.nestlechoosewellness.com.au/recipes-com-au/recipe-collections/festive-other-occasions/christmas-festive/ |  | https://twitter.com/nestleaunews/status/542446120484339712 http://www.nestle.com/media/newsandfeatures/children-vegetables |
| A258 | Twitter | Information and messaging | Shape the evidence base on diet- and public health-related issues evidence | Nestle Australia ‏@nestleaunews RT @Nestle: #ICYMI: How can we solve the problem of Hidden Hunger? http://bddy.me/1ACdh2H   And then link to Nestle's website with publications funded by Nestle and then solutions with their products:  "A **study Nestlé commissioned, published last year, used a health economic model to calculate the total lifetime costs of iron, vitamin A and zinc deficiency in a group of children** aged six months to five years in the Philippines. The results were staggering. Direct medical costs were USD 30 million, while future production losses based on the group’s reduced capacity for work as adults were more than half a billion dollars. That’s not to mention the untold human cost of what added up to more than 122,000 ‘disability adjusted life years’, or years of healthy life lost to poor health.(...) In 2013 **we provided more than 167 billion servings of foods and beverages fortified with micronutrients** including iron, iodine and vitamin A. We’ve committed to increase that to 200 billion by 2016. (...) It’s something we’ve been discussing at the **III World Congress of Public Health Nutrition, where we’ve been presenting our research this week**. (...)" |  | https://twitter.com/nestleaunews/status/542143960483786752 |
| A259 | Twitter | Information and messaging | Shape the evidence base on diet- and public health-related issues evidence | Nestle Australia ‏@nestleaunews Could this theory change the way we see the long term effects of diet? http://bddy.me/1HSAu4B   With an infographic on epigenetics  Then link to a presentation  http://www.nestle.com/asset-library/documents/media/news-and-features/2014-november/nestle-epigenetics-infographic.pdf?app_data={%22pi%22%3A%2255653_1420710983_162358012%22%2C%22pt%22%3A%22twitter%22} "EPIGENETICS: How the experiences of previous generations can affect who we are (...) By studying the **potential epigenetic effects of people’s dietary habits**, we might be able to help future generations start healthier, and stay healthier for longer.(...) Nestlé’s epigenetics research is primarily focused on maternal and early life nutrition and health" | No references | https://twitter.com/nestleaunews/status/553304803862798336 |
| A260 | Twitter | Information and messaging | Shape the evidence base on diet- and public health-related issues evidence | Nestle Australia @nestleaunews · Jan 8  There are as many as 100 trillion microbes inside each of us. What are they doing? http://bddy.me/1Ki9ei7   Link to their website:  http://www.nestle.com/media/newsandfeatures/insight-annick-mercenier-bacteria-gut-health?app_data={%22pi%22%3A%2255653_1420558174_241013186%22%2C%22pt%22%3A%22twitter%22} "The gut’s ability to function properly depends on the interactions between its three main components: the microbes, the intestinal barrier and the immune system. By studying this ‘cross talk’ we can better understand how certain microbes in our gut can help to maintain our health, and why interfering with them may lead to problems. It’s a subject we’ll be discussing in depth at the Nestlé International Nutrition Symposium this week." | Information posted in October 2014 on Nestle's website | https://twitter.com/nestleaunews/status/553304727434174464 |
| A261 | Twitter | Information and messaging | Shape the evidence base on diet- and public health-related issues evidence | Nestle Australia @nestleaunews · 5h 5 hours ago  RT @Nestle: **How can what you ate as a child affect your health as an adult**? http://bit.ly/1Lpe4ed | Then link to their website: http://www.nestle.com/media/news/nihs-plymouth-university-study "The Nestlé Institute of Health Sciences, part of Nestlé’s global research and development network, has announced that it will continue **collaborating with the Plymouth University Peninsula Schools of Medicine and Dentistry in the UK** as part of a ground-breaking study into how childhood life, including diet, affects our health as adults. " | https://twitter.com/nestleaunews/status/562025261495877632 |
| A262 | Twitter | Information and messaging | Shape the evidence base on diet- and public health-related issues evidence | Nestle Australia ‏@nestleaunews  Kids are back to school. Tips on keeping their lunchbox healthy and interesting. http://bit.ly/16968Nb.#schoollunch … | Just after school holidays  Then link to their website: http://www.nestlechoosewellness.com.au/eating-well/for-kids-a-balanced-lunch/#schoollunch  "For more information on Healthy Kids Association click here" http://healthy-kids.com.au/ - which is Nestle's own association | https://twitter.com/nestleaunews/status/562030892856537088 |
| A263 | Twitter | Information and messaging | Shape the evidence base on diet- and public health-related issues evidence | Nestle Australia retweeted Healthy Bods @Healthy_Bods · 19h 19 hours ago Talking healthy lifestyles, perfect portions & aiming for 10,000 steps a day at @nestleaunews Choose Wellness stand | Promote physical activity  Person at the stand: Accredited Practising Dietitian on Sydney's Northern Beaches & North Shore passionate about good nutrition. Sydney healthybodsnutrition.com.au | https://twitter.com/Healthy_Bods/status/569791007551651840 |
| A264 | Twitter | Information and messaging | Shape the evidence base on diet- and public health-related issues evidence | Nestle Australia retweeted cereal4brekkie @cereal4brekkie · Feb 18 Talk to the experts on twitter chat tonight about how you can eat & feel better – 8-9pm AEDST. Follow on #AHWW @HealthyWtWeek @DAA_feed |  | https://twitter.com/cereal4brekkie/status/567901022326300672 |
| A265 | Uni | Information and messaging | Shape the evidence base on diet- and public health-related issues evidence | Sydney University School of Molecular Bioscience, the Faculty of Science: Nestle Australia - Uncle Tobys Prize in Nutrition Awarded annually to a student who is enrolled in senior Nutrition who demonstrates the greatest proficiency, provided the work is of sufficient merit. |  | http://sydney.edu.au/science/molecular_bioscience/current_students/prizes.php |
| A266 | Uni | Information and messaging | Shape the evidence base on diet- and public health-related issues evidence | Flinders University School of Health Science:  - The Uncle Tobys prize in Nutrition and Dietetics shall be awarded to the student enrolled in the Bachelor of Nutrition and Dietetics courses whose overall performance in the third year of the course is judged to be the best. - The Uncle Tobys prize in Nutrition and Dietetics shall be awarded to the student enrolled in the Master of Nutrition and Dietetics courses whose overall performance in the first year of the course is judged to be the best. |  | http://www.flinders.edu.au/sohs/sites/nutrition-and-dietetics/student-prizes.cfm |
| A267 | Uni | Information and messaging | Shape the evidence base on diet- and public health-related issues evidence | University of South Australia - Annual Food Industry Forum for Nutrition Research The forum is an annual event aimed at fostering dialogue between nutrition researchers and food manufacturers to exploit recent advances in nutritional science for the benefit of all stakeholders. [...] The 3rd Annual Food Industry Forum for Nutrition Research Healthy Food for a Healthier Australia was held on Monday 3 September 2012 [...]. Major sponsors of the Forum were the Australian Food and Grocery Council and Nestle Australia. Supporting sponsors were Simplot Australia, Grains & Legumes Nutrition Council, Unilever Australia, Mannatech Australasia, the Nutrition Society of Australia, Kellogg's, Meat & Lifestock Australia and Sanitarium. |  | http://www.unisa.edu.au/Research/Sansom-Institute-for-Health-Research/Research-at-the-Sansom/Research-Concentrations/Nutritional-Physiology/Annual-Food-Industry-Forum-for-Nutrition-Research/ |
| A268 | FOI request | Information and messaging | Shape the evidence base on diet- and public health-related issues evidence | February 29th, 2012 Nestle Australia Ltd Response to Australian Dietary Guidelines Incorporating the Australian Guide to Healthy Eating. Draft for Public Consultation National Health and Medical Research Council December 2011 | Nestle asked for this submission to be confidential  References contain mostly evidence funded by the industry: ° Fayet F, Ridges L, Sritharan N, Petocz P - unpublished - poster presentation  ° Gibson SA (1999) "This work was supported by a grant from the Kellogg Company (UK) Limited."  ° Maughan and Griffin (2003) "This review was supported by PG Tips tea from Brooke Bond, part of Unilever Bestfoods, UK." ° Frary CD, Johnson RK and Wang MQ (2004) "Funding was provided by the Northeast Dairy Foods Research Center." ° Albertson AM, Thompson DR, and Franko DL (2009) - first author: "Bell Institute of Health and Nutrition, General Mills, Inc.," ° Murphy et al. (2008) "Funding for this research was provided by the National Dairy Council." ° Johnson et al. (2002) - "This research was funded by the National Dairy Council." ° Hainer V, Toplak H, Mitrakou A, - This article is based on a presentation at the 1st World Congress of Controversies in Diabetes, Obesity and Hypertension (CODHy). The Congress and the publication of this article were made possible by unrestricted educational grants from MSD, Roche, sanofi-aventis, Novo Nordisk, Medtronic, LifeScan, World Wide, Eli Lilly, Keryx, Abbott, Novartis, Pfizer, Generx Biotechnology, Schering, and Johnson & Johnson" ° Jensen et al (2004) "The Kellogg Company provided unrestricted funding of the development of the whole-grain database" ° Giacco R et al (2009)"This study was supported in part by funds from R&D Barilla G&R F.lli. SpA, Parma, Italy" ° Harris K, Kris-­‐Etherton P. (2010) - "PM Kris-Etherton’ s and KA Harris’ employer has received a grant from General Mills to conduct clinical trials assessing the effect of whole grains on metabolic syndrome. KA Harris is supported by the Nestle PhD, RD Training Fellowship, which is a competitive award funded by Nestle Research Center for a nutritional science graduate student pursuing both degrees at the Pennsylvania State University" ° Jonnalagadda S, Harnack L, Liu R, et al. (2010): unpublished - symposium ° Brownlee I, Moore C, Chatfield M, et al. (2010) - one of the author: Peter Ashb - Cereal Partners Worldwide ° Kristensen (2012) - authors: Department of Bioanalytical Sciences, Nestle Research Centre, Lausanne, Switzerland and Barilla, Parma, Italy ° Tosh S et al (2010)- one author from PepsiCo R&D Nutrition ° Maki et al (2010)- " This trial was funded by General Mills Bell Institute of Health and Nutrition, Minneapolis, MN" |  |
| A269 | Industry | Policy substitution | Policy substitution | Nestle Professional "We firmly believe that healthy food can also taste delicious. We recently took the lead when **we reduced the salt and fat content** from most of our MAGGI sauces and bouillons. And Nestlé's 100 professional chefs are continuing to work towards skilfully r**educing sodium, sugar, trans fatty acids and saturated fat in some of our other food and beverages** without compromising on the taste your customers love. " |  | https://www.nestleprofessional.com/australia/en/OurCompany/Our_Responsibility/Pages/Nutrition_Health_and_Wellness.aspx |
| A270 | Government | Policy substitution | Policy substitution | FSANZ Proposal 293 Nutrition Health and Related Claims Consultation Submission by Nestle: On fat-free and % fat-free claims: "Nestlé considers that the **education element is already being addressed** through the industry developed % Daily Intake Guide (DIG) front of pack nutrition communication. The program has been in place for several years with the industry uptake continually growing. The next evolution of the DIG along with the **wider government and industry collaboration** to support the scheme will ensure that the program infiltrates further capturing a wider consumer base and helping them to remain informed about their food choices." [page 10] |  | http://www.foodstandards.gov.au/code/proposals/Documents/P293_consultation_2012.zip |
| A271 | Twitter | Policy substitution | Policy substitution | NestléVerified account ‏@Nestle We're reducing the salt in our products, but how can you reduce your intake at home? http://bddy.me/1td3MSN   Then on the website:  At Nestlé, we have a wide variety of low salt recipes to help you reduce salt in your diet. See what works for you, add your own twists, and then spread the word. [...] We offer a range of low salt options and we're working to reduce salt even further across our savoury food products, including Maggi soups and noodles, and Buitoni pizzas. [...] We’ve been gradually reducing the salt levels in our food since 2005. Why? To give customers time to get used to changing flavour, helping them to adapt their taste preferences over the long term  [...] At Nestlé, our chefs are always looking for different ways to enhance flavour, by using different spices and herbs instead of salt. | Personal responsibility and good traits of the industry Link to their website with promotion of products | https://twitter.com/Nestle/status/536504454351425537 http://www.nestle.com/nutrition-health-wellness/health-wellness-tips/ways-to-eat-less-salt?app_data={%22pi%22%3A%2255653_1415629660_2130004621%22%2C%22pt%22%3A%22twitter%22} |
| A272 | Industry | Policy substitution | Policy substitution | "Serving up new approaches to Portion Guidance Introducing latest research and new initiatives to help people understand portions and balance their diets. INFORMATION FOR HEALTHCARE PROFESSIONALS" "Nestlé encourages **responsible nutrition, moderation and variety** in food habits (...) For this reason we are developing portion guidance at a product level with clear illustrations, product form and pack design that you will learn about here. Additional education materials and resources will accompany these new initiatives, including tools specifically designed to support healthcare professionals." |  | http://www.nestle.com.au/asset-library/documents/nco134confectionery%20portion%20guidance%2012pp%20080514-3.pdf |
| A273 | Government | Policy substitution | Policy substitution | FSANZ Proposal 293 Nutrition Health and Related Claims Consultation Submission by Nestle: "**Co-regulation** will also assist in reducing the burden of enforcement for jurisdictions" [page 4] On fat-free and % fat-free claims: "Nestlé would support Option 2 - a **voluntary industry Code of Practice** in relation to high sugar, high energy confectionery products (only) that do not normally contain significant levels of fat." [page 10] |  | http://www.foodstandards.gov.au/code/proposals/Documents/P293_consultation_2012.zip |
| A274 | Government | Policy substitution | Policy substitution | Food and Health Dialogue - Industry roundtable participants include: Nestle Australia Ltd |  | http://www.foodhealthdialogue.gov.au/internet/foodandhealth/publishing.nsf/Content/about-us |
| A275 | Industry | Policy substitution | Policy substitution | "Nestlé has a process in place to better ensure global compliance with our Consumer Communication Principles and Policy on Marketing Communication to Children. This includes a set of Implementation Guidelines, support and a monitoring system. " | For specific details - see Sacks et al 2014 | http://www.nestle.com.au/aboutus/home/responsible-advertising--marketing |
| A276 | Industry | Policy substitution | Policy substitution | "As part of our continuous improvement **we are renovating our products and packaging so that they provide clear portion guidance**." "**Nestlé Nutrition Profiling System** (NNPS)  NNPS is a rigorous system used to benchmark our products annually against set criteria. It assesses a product's nutritional contribution, considering its role in a balanced diet, its ingredients (including fat, added sugar, calcium and wholegrain) and the serving size usually consumed, either by adults or children. Since implementation, we have renovated the Nestlé portfolio representing 70% of net sales to meet the Nutrition Foundation criteria. As part of our continuous improvement our ambition is to reach 80% by 2016." | No independent criteria | http://www.nestle.com.au/nhw/nestle-portion-plates |
| A277 | Twitter | Policy substitution | Policy substitution | NestléVerified account ‏@Nestle Cutting down on salt doesn't have to be difficult: http://bddy.me/1srvUQQ   Then on the website: At Nestlé, we have a wide variety of low salt recipes to help you reduce salt in your diet. See what works for you, add your own twists, and then spread the word. [...] We offer a range of low salt options and we're working to reduce salt even further across our savoury food products, including Maggi soups and noodles, and Buitoni pizzas. [...] We’ve been gradually reducing the salt levels in our food since 2005. Why? To give customers time to get used to changing flavour, helping them to adapt their taste preferences over the long term [...] At Nestlé, our chefs are always looking for different ways to enhance flavour, by using different spices and herbs instead of salt. | Link to their website with promotion of products | https://twitter.com/Nestle/status/531815553762332672 http://www.nestle.com/nutrition-health-wellness/health-wellness-tips/ways-to-eat-less-salt?app_data={%22pi%22%3A%2255653_1415629660_2130004621%22%2C%22pt%22%3A%22twitter%22} |
| A278 | Twitter | Policy substitution | Policy substitution | Nestle Australia ‏@nestleaunews Check out our new on-pack Portion Device to help avoid 'portion distortion' http://bit.ly/1pVscEl  And on the website:  Nestlé has launched a new tool to help avoid ‘portion distortion’. The new on-pack Portion Device aims to **educate consumers** on appropriate food portions. Research shows that over the years, people’s perception of what constitutes a serve, or a portion of food, has slowly been increasing. Studies suggest that consumers select substantially larger portions due to a phenomenon known as **‘portion distortion**’ – perceiving large portion sizes as appropriate amounts to eat in a single eating occasion1. | Link to their website: http://www.nestlechoosewellness.com.au/your-wellbeing/nestle-launches-innovative-a-new-portion-device/ | <https://twitter.com/nestleaunews/status/540264244483076097> |

## Woolworths

| **Reference in manuscript** | **Source** | **Strategy** | **Practice (code used for analysis)** | **Data coded** | **Notes** | **Website URL** |
| --- | --- | --- | --- | --- | --- | --- |
| A279 | Charity | Constituency building | Seek involvement in the community | The Love Food Hate Waste campaign is managed by the EPA partnering with corporate, government and not-for-profit organisations. Our inaugural partners include:  Woolworths Ltd. |  | http://www.lovefoodhatewaste.nsw.gov.au/about/frequently-asked-questions.aspx |
| A280 | CSR | Constituency building | Seek involvement in the community | o A Great Partner in the community: $47.6m Invested in community partners $7.2m Worth of educational equipment delivered to schools through Earn & Learn $5.5m Raised in response to bushfires and drought NZ$1.2m Raised in the Countdown Kids Hospital Appeal 1.8m Meals made from food donations" o Partnerships and programs: The Salvation Army, Avner Nahmani Pancreatic Cancer Foundation, Food Bank, Earn and Learn" o Woolworths supermarkets and petrol:  "State Charity Contribution NS W/ACT Variety, the children’s charity $598,000 QLD Children’s Hospital Foundation $3,950,000 VIC/TAS Royal Children’s Hospital Foundation $500,000 SA /NT Royal Flying Doctor Service $104,000 WA Telethon $250,000" | Specific examples and details in the report [online] | http://woolworthslimited2014.csr-report.com.au/ |
| A281 | Industry | Constituency building | Seek involvement in the community | Woolworths National Charity Partners include: The Salvation Army Jamie’s Ministry of Food Foodbank  Woolworths State Charity Partners include: Children’s Hospital Foundation (QLD) Variety –the Children’s Charity (NSW & ACT) Good Friday Appeal (VIC) Give Me 5 For Kids (TAS) Starlight Children’s Foundation (SA & NT) Channel 7 Telethon Trust (WA) |  | http://www.woolworths.com.au/wps/wcm/connect/website/woolworths/about+us/ community/charity+partners/charity+partners |
| A282 | Industry | Constituency building | Seek involvement in the community | Fundraising BBQs - Raise money for your local community |  | http://www.woolworths.com.au/wps/wcm/connect/Website/Woolworths/About+Us/ community/fundraisingbbqs/ |
| A283 | Industry | Constituency building | Seek involvement in the community | "Building additional capacity through a major grants scheme. In 2011, Woolworths contributed $2 million to help charity groups expand their operations and ensure thousands more people can access healthy, nutritious food. More than 100 food relief charities benefited from infrastructure grants for vital items, such as vans, refrigerators, freezers and kitchen equipment. Prior to Christmas 2011 Woolworths donated $50,000, shared between 11 of our key partners, to provide important support during a particularly busy time for these partners." |  | http://www.woolworths.com.au/wps/wcm/connect/website/woolworths/about+us/ community/05other-projects/fresh+food+rescue |
| A284 | Industry | Constituency building | Seek involvement in the community | "More than 13,148 schools and early learning centres redeemed their Woolworths Earn & Learn Points for new educational equipment – in fact more than 400,000 items of new educational equipment are being delivered to schools right around the country. [...] Equipment categories that were popular with Primary Schools and High Schools were Mathematics, English and Science, Arts & Crafts, Robotics, Sport, Music and Dance. Early learning centres redeemed their Woolworths Earn & Learn Points for equipment better suited to younger children, including resources for art & crafts, sand and water play, construction and sports equipment." |  | http://www.woolworths.com.au/wps/wcm/connect/website/woolworths/about+us/ community/woolworths+earn+and+learn/woolworthsearnandlearn |
| A285 | News | Constituency building | Seek involvement in the community | Channel 9 Children’s Hospital Telethon brought together celebrities, sports heroes, entertainers and everyday Queenslanders in support of the Children’s Hospital Foundation, as phones ran hot all night with badly-needed donations to the charity. The total raised by 11.30pm was more than $8.7 million.[...] Massive contributions from Australian companies also boosted the total, with Woolworths donating $3.7 million |  | http://www.couriermail.com.au/news/queensland/telethon-raises-millions-of-dollars-for-sick-children-and-their-families/story-fnihsrf2-1227087671363?nk=6478f989f25376080e0eee435fd3a79e |
| A286 | News | Constituency building | Seek involvement in the community | Campaign helps sick little girl get help  THE face of little Emmy-Claire Clarke, 5, was seen covering the walls of Toowoomba Woolworths stores this month. She also starred on the Woolworths carnival float as part of a local fundraiser for the Children's Hospital Foundation. |  | http://www.thechronicle.com.au/news/woolworths-raise-funds-for-life-saving-equipment/2399351/ |
| A287 | News | Constituency building | Seek involvement in the community | THE face of local four-year-old patient Jack Rethamel covered the walls of Bundaberg Woolworths stores this month as part of a fundraiser for the Children's Hospital Foundation which raised more than $7900 for regional children's wards. |  | http://www.news-mail.com.au/news/woolworths-tokens-raise-funds-for-sick-kids/2410922/ |
| A288 | News | Constituency building | Seek involvement in the community | "WOOLWORTHS supermarkets on the Fraser Coast have raised a total of $74,698 this year, with funds going to the Children's Hospital Foundation.” |  | http://www.frasercoastchronicle.com.au/news/woolies-still-committed/2479776/ |
| A289 | News | Constituency building | Seek involvement in the community | Woolworths has kicked off a new campaign promoting its sponsorship of the ballkids at a number of Australian tennis events as part of its partnership with Tennis Australia. The supermarket will sponsor the ballkids at The Australian Open, the Australian Open Series, the Pro Tour and the ballkid training program and will work with Tennis Australia to enhance the program with the aim to increase registrations |  | http://mumbrella.com.au/woolworths-unveils-ad-promoting-sponsorship-tennis-ballkids-271001 |
| A290 | News | Constituency building | Seek involvement in the community | WOOLWORTHS has donated almost $8000 to the Bundaberg Health Services Foundation with funds raised from its two-week wall token campaign. All proceeds from the sale of $2 wall tokens at Bundaberg's Woolworths stores was donated to the Bundaberg Hospital children's ward to fund the purchase of equipment and resources.  [...] Woolworths stores across Queensland will continue to support the Children's Hospital Foundation through various in-store fundraising activities in 2015.  **Woolworths has partnered with the Children's Hospital Foundation for more than 28 years, with $31.2 million raised for children's hospitals and ongoing research.** |  | http://www.news-mail.com.au/news/kids-helped-by-wall-tokens/2541560/ |
| A291 | Government | Constituency building | Establish relationships with key opinion leaders and health organisations | Dietary Guidelines Working Committee - Conflict of interest information: *Dr Rosemary Stanton Received payment from Woolworths less than five years ago to develop their Fresh Foods advertising campaign, which promoted fruits and vegetables for children. |  | http://www.nhmrc.gov.au/your-health/nutrition/dietary-guidelines-working-committee/declarations-conflict-interest-dietary |
| A292 | CSR | Constituency building | Establish relationships with key opinion leaders and health organisations | "The ‘Jamie’s Garden’ sticker album and collectable stickers (...) are introducing kids to **information** on healthy nutrition, fresh ingredients and the origin of food." "We have partnered with **Nutrition Australia** to **make it easier for people to buy nutritious snacks** in the Healthier Bites section of our food stores." "This star rating allows customers to **make quick and easy informed choices about the nutrition of a packaged product**." | Specific examples and details in the report [online] | http://woolworthslimited2014.csr-report.com.au/ |
| A293 | PH | Constituency building | Establish relationships with key opinion leaders and health organisations | Nutrition Australia corporate partners: Woolworths |  | http://www.nutritionaustralia.org/national/our-partners/nutrition-australia-corporate-partners#.U88kY0DpfXI |
| A294 | Government | Constituency building | Establish relationships with policy makers | The following organisations form membership of the Dialogue: (Food and Health Dialogue) Woolworths Ltd  Membership of the Reformulation Working Group comprises: Woolworths Ltd  Industry roundtable participants include: Woolworths Ltd |  | http://www.foodhealthdialogue.gov.au/internet/foodandhealth/publishing.nsf/Content/about-us |
| A295 | Industry | Constituency building | Establish relationships with policy makers | "Health and Wellbeing - Woolworths is a member of the Australian Government's Food and Health Dialogue, which works with industry on a range of voluntary reformulation of commonly consumed foods, including bread and cereals." |  | http://www.woolworthslimited.com.au/page/A_Trusted_Company/Responsible_Service/ |
| A296 | Government | Constituency building | Establish relationships with policy makers | Front-of-Pack Labelling Committee and Working Group meetings Implementation Working Group – Meeting 1 - 5 June 2012 The IWG members have been selected on the basis of their expertise and experience in the following fields and do not represent their organisations as working group members (9 members in total): Joelle Day - Industry (Woolworths) Roger Bektash - Industry (Mars) Jodi Dixon - Industry (Coles) |  | http://www.health.gov.au/internet/main/publishing.nsf/Content/frontofpackcommittee |
| A297 | CSR | Financial incentive | Financial incentives | "This year, political contributions in Australia totalled $35,120." |  | http://woolworthslimited2014.csr-report.com.au/ |
| A298 | Government | Financial incentive | Financial incentives | 44th Parliament: Members' Interest Statements MP Anthony Albanese: "On 11 December 2013, I received a hamper from Woolworths Limited" |  | http://www.aph.gov.au/~/media/03%20Senators%20and%20Members/32%20Members/Register/44p/AB/AlbaneseA_44P.pdf |
| A299 | Political party | Financial incentive | Financial incentives | Political Party Disclosure Return FINANCIAL YEAR 2011-12 Australian Labor Party Name: Woolworths Limited $ 1,500 $3,000 $12,000 |  | http://periodicdisclosures.aec.gov.au/Returns/49/PNGX9.pdf |
| A300 | Political party | Financial incentive | Financial incentives | Political Party Disclosure Return FINANCIAL YEAR 2011-12 Liberal Party of Australia (Victorian Division) Name: Woolworths Limited $25,000 |  | http://periodicdisclosures.aec.gov.au/Returns/49/PQRZ7.pdf |
| A301 | Political party | Financial incentive | Financial incentives | Political Party Disclosure Return FINANCIAL YEAR 2012-13 Australian Labor Party Name: Woolworths Limited $27,500 $8,250 $12,000 |  | http://periodicdisclosures.aec.gov.au/Returns/51/RANF1.pdf |
| A302 | CSR | Information and messaging | Stress the economic importance of the industry | "A Great Place to Work:  198,642 Employees  75,923 Young employees  2,156 Indigenous Australian employees […] A Great Place to invest:  $1.7bn Dividends paid to investors, many of whom are mum and dad investors $114bn Indirect contribution to Australian economy $2.4bn Paid in taxes" | Specific examples and details in the report [online] | http://woolworthslimited2014.csr-report.com.au/ |
| A303 | Industry | Information and messaging | Stress the economic importance of the industry | " As a proud Australian company, we are extensively involved in every community we serve through our commitment to:  our staff by employing more than 110,000 Australians;  our suppliers by supporting thousands of farmers and producers and indirectly contributing about $600 billion into the economy every year" |  | http://www.woolworthslimited.com.au/page/A_Trusted_Company/Australias_Fresh_Food_People/ |
| A304 | Government | Information and messaging | Frame the debate on diet- and public health-related issues the debate on diet- and public health-related issues | FSANZ Proposal 293 Nutrition Health and Related Claims Consultation Submission by Woolworth: "Woolworths supports consumer, health profession and government initiatives to **help consumers make informed food choices**." [page 1] "we have an **important role to play** in **promoting balanced and healthy eating habits that support a healthy lifestyle**" [page 1] "**We have already made significant steps to promote healthy diets to Australian shoppers** by **adapting our store format, improving the availability and affordability of fresh food, and educating parents and children about the importance of a balanced diet**." [page 1] "**We have also adopted the Australian Food and Grocery Council’s (AFGC) Daily Intake Guide (DIG)**, which provides consumers with **easy to digest information** about their food choices." [page 1] "**Woolworths is also part of the Australian Government’s Food and Health Dialogue**, which is a joint government-industry-public health initiative aimed at addressing poor dietary habits and making healthier food choices easier and more accessible for all Australians. Woolworths is involved in all levels of the Dialogue including the Executive Group, a Reformulation Working Group and industry roundtables. Woolworths participates in a voluntary reformulation program across a range of commonly consumed foods for key nutrients including sodium, saturated fat, sugar, fibre, fruit and vegetables." [page 1] "**Woolworths continues to develop and improve a range of products across our brands** which have been carefully selected to care for our customers' health and wellbeing. Developments include improving salt, sugar and fat levels and minimising the use of additives. This includes our Macro brand which aims to offer alternatives to satisfy dietary or lifestyle choices." [page 2] |  | http://www.foodstandards.gov.au/code/proposals/Documents/P293_consultation_2012.zip |
| A305 | Industry | Information and messaging | Frame the debate on diet- and public health-related issues the debate on diet- and public health-related issues | "Not sure what your daily energy requirements should be? View our tips on maintaining a **balanced diet**. You will also learn how to read Daily Intake Guide thumbnails. […] Food labels help you **make safe and healthy product choices**" |  | http://www.woolworths.com.au/wps/wcm/connect/Website/Woolworths/Fresh+Living/Health+and+Wellbeing/Healthy+Shopping/Product-Labelling/ |
| A306 | Government | Information and messaging | Frame the debate on diet- and public health-related issues the debate on diet- and public health-related issues | FSANZ Proposal 293 Nutrition Health and Related Claims Consultation Submission by Woolworth: "Woolworths is committed to empowering our **customers to make informed choices** when selecting grocery products." [page 1] "Helps **customers understand** whether a food is providing a little or a lot of key nutrients and how it fits into their daily requirements." [page 1] "We strongly believe **our customers should have access to a full suite of nutritional Information to enable them to make informed decisions when selecting groceries**." [page 2] |  | http://www.foodstandards.gov.au/code/proposals/Documents/P293_consultation_2012.zip |
| A307 | Lobby | Information and messaging | Lobby Establish relationships with policy makers | Lobby register Endeavour Consulting Group Pty Ltd & Newgate Communications Pty Limited Client: Woolworths |  | http://lobbyists.pmc.gov.au/export/export_client.cfm |
| A308 | Government | Policy substitution | Promote deregulation | FSANZ Proposal 293 Nutrition Health and Related Claims Consultation Submission by Woolworth: "The NPSC does not therefore fully assess the complete nutrient profile of the food. As a result, an unintended consequence of this method could be to **discourage nutritional improvement and innovation** in products which are ineligible for health claims" "reduce consumer choice." [page 2] |  | http://www.foodstandards.gov.au/code/proposals/Documents/P293_consultation_2012.zip |
| A309 | CSR | Policy substitution | Policy substitution | "In Australia, we are working with the Federal Government and public health groups through the **Food & Health Dialogue** to provide healthier choices for consumers. In New Zealand, we are working with **HeartSafe NZ**." | Specific examples and details in the report [online] | http://woolworthslimited2014.csr-report.com.au/ |
| A310 | CSR | Policy substitution | Policy substitution | "Since the launch last year, we have worked with Nutrition Australia to **cut portion sizes, limit added sugars and increase the amount of wholegrain**, dietary fibre and core food content in snacks" "In June, we were the first Australian retailer to adopt the Federal Government’s **voluntary Health Star Rating Scheme**." | Specific examples and details in the report [online] | http://woolworthslimited2014.csr-report.com.au/ |
| Free code^[[1]](#footnote-1)^ | Government |  | Free code | 44th Parliament: Members' Interest Statements MP Gillespie: Shareholdings: Woolworths |  | http://www.aph.gov.au/~/media/03%20Senators%20and%20Members/32%20Members/Register/44p/GK/GillespieD_44P.pdf |
| Free code | Government |  | Free code | 44th Parliament: Members' Interest Statements MP Hendy's wife: Shareholdings: Woolworths |  | http://www.aph.gov.au/~/media/03%20Senators%20and%20Members/32%20Members/Register/44p/GK/HendyP_44P.pdf |

1. Practices coded as “free code” are practices where the food industry is not proactively using the CPA [↑](#footnote-ref-1)
