# Supplementary material for: Systematic examination of publicly-available information reveals the diverse and extensive corporate political activity of the food industry in Australia
Source: BMC Public Health. 2016 Mar 22;16:283. doi: 10.1186/s12889-016-2955-7 (PMC4804618; doi:10.1186/s12889-016-2955-7)
Supplement: Additional file 4: — Examples of third parties funded or affiliated with the sample of food industry actors in Australia. (DOCX 19 kb) [file 12889_2016_2955_MOESM4_ESM.docx]

Additional file 4: Examples of third parties funded or affiliated with the sample of food industry actors in Australia

|  |  | **Australian Food and Grocery Council** | **Coca-Cola** | **Mc Donald’s** | **Nestle** | **Woolworths** |
| --- | --- | --- | --- | --- | --- | --- |
| **Name of group** | **Affiliation with the food industry (webpage)** | **Food industry actors affiliated** | | | | |
| Australian Beverages Council | Members (http://australianbeverages.org/about-us/member-directory/) |  | X |  | X |  |
| Australian Breakfast Cereal Manufacturers Forum | Founder/members (http://www.cereal4brekkie.org.au/abcmf/) | X |  |  | X |  |
| Business Council of Australia | Members (http://www.bca.com.au/about-us/our-members) |  | X | X |  | X |
| Centre for Independent Studies | Board of directors (http://www.cis.org.au/about-cis/board-of-directors) |  | X |  |  |  |
| Daily Intake Guide | Founder (http://www.mydailyintake.net/) | X |  |  |  |  |
| Dairy Australia | Members (http://www.dairyaustralia.com.au/Industry-information/About-Dairy-Australia/Who-we-are/Members.aspx  http://www.adpf.org.au/members.asp) |  |  |  | X |  |
| Enactus | Board of directors (http://enactusaustralia.org.au/business_involvement/board_of_directors/) | X | X |  |  | X |
| Fruit Juice Australia | Members (http://fruitjuiceaustralia.org/fja_members/) |  | X |  |  |  |
| Grains & Legumes Nutrition Council | Contributors (http://www.glnc.org.au/about-us/contributors/) | X |  |  | X |  |
| International Life Science Institute Southeast Asia region | Members (http://www.ilsi.org/SEA_Region/Pages/Membership.aspx) |  | X |  | X |  |
| Infant Nutrition Council | Members (http://www.infantnutritioncouncil.com/membership/current-members/) |  |  |  | X |  |
| Quick Service Restaurant Forum | Members (http://www.afgc.org.au/our-expertise/afgc-supported-forums/quick-service-restaurant-qsr-forum/) | X |  | X |  |  |
| Together Counts | Partners (http://www.togethercounts.com.au/) |  | X |  | X |  |
